# Supplementary material for: Using a pragmatically adapted, low-cost contingency management intervention to promote heroin abstinence in individuals undergoing treatment for heroin use disorder in UK drug services (PRAISE): a cluster randomised trial
Source: BMJ Open. 2021 Jul 1;11(7):e046371. doi: 10.1136/bmjopen-2020-046371 (PMC8252884; doi:10.1136/bmjopen-2020-046371)
Supplement: Supplementary data [file bmjopen-2020-046371supp001.pdf]

Metrebian, Weaver & Goldsmith et al. Using a pragmatically adapted, low-cost, contingency management intervention to promote heroin abstinence in individuals undergoing treatment for heroin-use disorder in UK drug services (PRAISE): a cluster randomised trial

Supplementary material

Table 1. Clinic characteristics by Arm

		CM Abstinence			CM Attendance			TAU			TOTAL		
		N	Mean / or median %	SD or IQR	N	Mean / or median %	SD or IQR	N	Mean / or median %	SD or IQR	N	Mean / or median %	SD or IQR
Clinic type<br>N (%) NHS		6	55		7	58		8	73		21	62	
Clinic location<br>N (%) London		7	63		3	25		6	55		16	47	
Birmingham		2	18		2	17		1	9		5	15	
Dudley & Walsall		0	0		1	8		0	0		1	3	
Herts		1	9		4	33		1	9		6	18	
South Essex		1	9		1	8		0	0		2	6	
Sussex		0	0		1	8		2	18		3	9	
Avon & Wiltshire		0	0		0	0		1	9		1	3	
Clinic total		11	100		12	100		11	100		34	100	

Table 2 Opiate Treatment Index (OPI) measures of substance use in last 30 days. Use in last 30 days (binary) and N of days used. (Missing shown but n% proportions calculated disregarding missing

	**CM Abstinence n=205**	**CM Attendance n = 174**	**TAU n= 173**	**Total n = 552**
**CRACK**				
**Baseline**				
Used crack in last 30 days	161 (79)	128 (74)	130 (75)	419 (76)
Did not use crack in last 30 days	44 (22)	46 (26)	43 (25)	133 (24)
Missing	*0*	*0*	*0*	*0*
**12 weeks**				
Used crack in last 30 days	75 (57)	73 (52)	62 (53)	210 (54)
Did not use crack in last 30 days	57 (43)	68 (48)	54 (47)	179 (46)
Missing	*73*	*33*	*57*	*163*
**24 weeks**				
Used crack in last 30 days	78 (63)	56 (51)	52 (58)	186 (58)
Did not use crack in last 30 days	45 (37)	53 (49)	37 (42)	135 (42)
Missing	*82*	*65*	*84*	*231*
Number of days taking crack in last 30 days				
**Baseline**				
n	203	174	172	549
Number missing	2	0	1	3
Proportion missing	0.98	0	0.58	0.54
Median (25th, 75th percentile)	10.00 (1.00, 26.00)	7.00 (0.00, 20.00)	5.00 (0.50, 24.50)	7.00 (1.00, 24.00)
**12 weeks**				
n	132	140	116	388
Number missing	73	34	57	164
Proportion missing	35.61	19.54	32.95	29.71

Median (25th, 75th percentile)	1.00 (0.00, 10.00)	1.00 (0.00, 4.00)	1.00 (0.00, 4.00)	1.00 (0.00, 5.00)
**24 weeks**				
n	123	109	89	321
Number missing	82	65	84	231
Proportion missing	40	37.36	48.55	41.85
Median (25th, 75th percentile)	2.00 (0.00, 6.00)	1.00 (0.00, 4.00)	1.00 (0.00, 4.00)	1.00 (0.00, 5.00)
**BENZODIAZEPENES**				
**Baseline**				
Used benzos in last 30 days	47 (23)	35 (20)	47 (27)	129 (23)
Did not use benzos in last 30 days	158 (77)	138 (80)	126 (73)	422 (77)
Missing	*0*	*1*	*0*	*1*
**12 weeks**				
Used benzos in last 30 days	20 (15)	16 (11)	26 (22)	62 (16)
Did not use benzos in last 30 days	111 (85)	125 (89)	90 (78)	326 (84)
Missing	*74*	*33*	*57*	*164*
**24 weeks**				
Used benzos in last 30 days	20 (16)	9 (8)	16 (18)	45 (14)
Did not use benzos in last 30 days	103 (84)	100 (92)	73 (82)	276 (86)
Missing	*82*	*65*	*84*	*231*
Number of days taking benzodiazepines in last 30 days				
**Baseline**				
n	205	172	173	550
Number missing	0	2	0	2
Proportion missing	0	1.15	0	0.36
Median (25th, 75th percentile)	0.00 (0.00, 0.00)	0.00 (0.00, 0.00)	0.00 (0.00, 1.00)	0.00 (0.00, 0.00)
**12 weeks**				
n	131	139	116	386
Number missing	74	35	57	166
Proportion missing	36.10	20.11	32.95	30.07

Median (25th, 75th percentile)	0.00 (0.00, 0.00)	0.00 (0.00, 0.00)	0.00 (0.00, 0.00)	0.00 (0.00, 0.00)
**24 weeks**				
n	123	109	89	321
Number missing	82	65	84	231
Proportion missing	40.00	37.36	48.55	41.85
Median (25th, 75th percentile)	0.00 (0.00, 0.00)	0.00 (0.00, 0.00)	0.00 (0.00, 0.00)	0.00 (0.00, 0.00)
**ALCOHOL**				
**Baseline**				
Used alcohol in last 30 days	117 (57)	93 (53)	100 (58)	310 (56)
No alcohol use in last 30 days	88 (43)	81 (47)	73 (42)	242 (44)
Missing	*0*	*0*	*0*	*0*
**12 weeks**				
Used alcohol in last 30 days	77 (58)	83 (59)	76 (66)	236 (61)
No alcohol use in last 30 days	55 (42)	59 (42)	40 (35)	154 (40)
Missing	*73*	*32*	*57*	*162*
**24 weeks**				
Used alcohol in last 30 days	70 (57)	67 (62)	51 (57)	188 (59)
No alcohol use in last 30 days	53 (43)	42 (39)	38 (43)	133 (41)
Missing	*82*	*65*	*84*	*231*
Number of days taking alcohol in last 30 days				
**Baseline**				
n	204	172	172	548
Number missing	1	2	1	4
Proportion missing	0.49	1.15	0.58	0.72
Median (25th, 75th percentile)	1.00 (0.00, 10.00)	1.00 (0.00, 10.00)	1.00 (0.00, 8.00)	1.00 (0.00, 10.00)
**12 weeks**				
n	132	142	115	389
Number missing	73	32	58	163
Proportion missing	35.61	18.39	33.53	29.53

Median (25th, 75th percentile)	1.00 (0.00, 9.50)	2.00 (0.00, 10.00)	2.00 (0.00, 8.00)	2.00 (0.00, 10.00)
**24 weeks**				
n	123	107	89	319
Number missing	82	67	84	233
Proportion missing	40.00	38.51	48.55	42.21
Median (25th, 75th percentile)	1.00 (0.00, 9.00)	2.00 (0.00, 10.00)	2.00 (0.00, 12.00)	2.00 (0.00, 10.00)
**TOBACCO**				
**Baseline**				
Used tobacco in last 30 days	198 (97)	167 (96)	166 (96)	531 (96)
No tobacco use in last 30 days	7 (3)	7 (4)	7 (4)	21 (4)
Missing	*0*	*0*	*0*	*0*
**12 weeks**				
Used tobacco in last 30 days	129 (98)	134 (94)	107 (93)	370 (95)
No tobacco use in last 30 days	3 (2)	8 (6)	8 (7)	19 (5)
Missing	*73*	*32*	*58*	*163*
**24 weeks**				
Used tobacco in last 30 days	119 (97)	101 (93)	79 (89)	299 (93)
No tobacco use in last 30 days	4 (3)	8 (7)	10 (11)	22 (7)
Missing	*82*	*65*	*84*	*231*
**Number of days taking tobacco in last 30 days**				
**Baseline**				
n	198	171	171	540
Number missing	7	3	2	12
Proportion missing	3.41	1.72	1.16	2.17
Median (25th, 75th percentile)	30.00 (30.00, 30.00)	30.00 (30.00, 30.00)	30.00 (30.00, 30.00)	30.00 (30.00, 30.00)
**12 weeks**				
n	131	139	113	383
Number missing	74	35	60	169
Proportion missing	36.10	20.11	34.68	30.62

Median (25th, 75th percentile)	30.00 (30.00, 30.00)	30.00 (30.00, 30.00)	30.00 (30.00, 30.00)	30.00 (30.00, 30.00)
**24 weeks**				
n	123	109	87	319
Number missing	82	65	86	233
Proportion missing	40.00	37.36	49.71	42.21
Median (25th, 75th percentile)	30.00 (30.00, 30.00)	30.00 (30.00, 30.00)	30.00 (30.00, 30.00)	30.00 (30.00, 30.00)

Table 3 Odds ratios for Opiate Treatment Index (OPI) measures of substance use in the last 30 days assessed at 12 and 24 weeks: Use in last 30 days (binary) and N of days used.

	Treatment group difference estimate	Lower confidence limit	Upper confidence limit	p-value
**CRACK**<br>**Use in last 30 days Y/N across groups – GEE model results**				
12 weeks CM Attendance v CM Abstinence	0.83	0.37	1.85	0.654
12 weeks TAU v CM Abstinence	0.90	0.46	1.78	0.768
12 weeks TAU v CM Attendance	1.08	0.59	1.99	0.795
24 weeks CM Attendance v CM Abstinence	0.71	0.37	1.37	0.307
24 weeks TAU v CM Abstinence	0.75	0.35	1.64	0.477
24 weeks TAU v CM Attendance	1.06	0.50	2.27	0.878
**Mean differences N of days used in last 30 days across groups**				
12 weeks CM Attendance v CM Abstinence	-1.80	-3.90	0.30	0.093
12 weeks TAU v CM Abstinence	-1.67	-3.84	0.50	0.132
12 weeks TAU v CM Attendance	0.13	-2.02	2.29	0.903
24 weeks CM Attendance v CM Abstinence	0.06	-2.13	2.25	0.956
24 weeks TAU v CM Abstinence	-0.30	-2.58	1.99	0.800
24 weeks TAU v CM Attendance	-0.36	-2.67	1.96	0.762

**BENZODIAZEPENES**<br>**Use in last 30 days Y/N across groups – GEE model results**				
12 weeks CM Attendance v CM Abstinence	0.65	0.20	2.13	0.480
12 weeks TAU v CM Abstinence	1.31	0.51	3.41	0.573
12 weeks TAU v CM Attendance	2.01	0.57	7.09	0.276
24 weeks CM Attendance v CM Abstinence	0.46	0.15	1.39	0.167
24 weeks TAU v CM Abstinence	0.88	0.33	2.32	0.794
24 weeks TAU v CM Attendance	1.92	0.70	5.28	0.204
**Mean differences N of days used in last 30 days across groups**				
12 weeks CM Attendance v CM Abstinence	-0.16	-0.94	0.62	0.688
12 weeks TAU v CM Abstinence	0.32	-0.49	1.14	0.436
12 weeks TAU v CM Attendance	0.48	-0.32	1.29	0.239
24 weeks CM Attendance v CM Abstinence	0.23	-0.61	1.06	0.591
24 weeks TAU v CM Abstinence	0.01	-0.87	0.90	0.976
24 weeks TAU v CM Attendance	-0.22	-1.12	0.69	0.642
**ALCOHOL**<br>**Use in last 30 days Y/N across groups – GEE model results**				
12 weeks CM Attendance v CM Abstinence	1.34	0.81	2.21	0.255
12 weeks TAU v CM Abstinence	1.55	0.78	3.07	0.208
12 weeks TAU v CM Attendance	1.16	0.59	2.29	0.674
24 weeks CM Attendance v CM Abstinence	1.86	1.01	3.45	0.048
24 weeks TAU v CM Abstinence	1.08	0.66	1.77	0.762
24 weeks TAU v CM Attendance	0.58	0.29	1.17	0.126
**Mean differences N of days used in last 30 days across groups**				
12 weeks CM Attendance v CM Abstinence	1.07	-0.59	2.72	0.206
12 weeks TAU v CM Abstinence	1.00	-0.74	2.75	0.259
12 weeks TAU v CM Attendance	-0.06	-1.78	1.65	0.943
24 weeks CM Attendance v CM Abstinence	0.20	-1.56	1.96	0.825
24 weeks TAU v CM Abstinence	0.87	-0.98	2.72	0.357

24 weeks TAU v CM Attendance	0.67	-1.21	2.56	0.485
**TOBACCO**<br>**Mean differences N of days used in last 30 days across groups**				
12 weeks CM Attendance v CM Abstinence	-0.50	-2.10	1.09	0.536
12 weeks TAU v CM Abstinence	-0.25	-1.93	1.42	0.769
12 weeks TAU v CM Attendance	0.25	-1.40	1.90	0.765
24 weeks CM Attendance v CM Abstinence	-0.95	-2.64	0.75	0.273
24 weeks TAU v CM Abstinence	-2.07	-3.87	-0.27	0.024
24 weeks TAU v CM Attendance	-1.13	-2.96	0.71	0.228

Table 4. Secondary Outcome Measures Summary Statistics: Alcohol Use Disorders (AUDIT), Social Functioning (OTI), physical and mental wellbeing (SF-36), depression and anxiety (HADS), crime (AD-SUS).

	CM Abstinence n=205	CM Attendance n = 174	TAU n= 173	Total n = 552
**AUDIT (Alcohol Use)**				
**Baseline**				
n	205	174	173	552
Number missing	0	0	0	0
Proportion missing	0	0	0	0
Mean (SD)	9.69 (10.42)	9.37 (9.71)	9.18 (9.88)	9.43 (10.02)
**12 weeks**				
n	131	142	116	389
Number missing	74	32	57	163
Proportion missing	36.10	18.39	32.95	29.53
Mean (SD)	8.79 (9.47)	8.39 (9.72)	8.13 (8.22)	8.45 (9.19)
**24 weeks**				
n	123	108	89	320
Number missing	82	66	84	232
Proportion missing	40.00	37.93	48.55	42.03
Mean (SD)	9.67 (9.46)	7.94 (9.01)	8.67 (8.62)	8.81 (9.08)
**SOCIAL FUNCTIONING**				
**Baseline**				
n	205	174	173	552
Number missing	0	0	0	0
Proportion missing	0	0	0	0
Mean (SD)	19.59 (6.36)	17.80 (7.41)	18.29 (6.41)	18.62 (6.75)
**12 weeks**				

n	132	143	115	390
Number missing	73	31	58	162
Proportion missing	35.61	17.82	33.53	29.35
Mean (SD)	17.45 (7.36)	16.31 (6.25)	16.37 (6.39)	16.71 (6.69)
**24 weeks**				
n	123	109	89	321
Number missing	82	65	84	231
Proportion missing	40	37.36	48.55	41.85
Mean (SD)	16.46 (7.07)	15.63 (6.69)	15.91 (5.96)	16.02 (6.64)
**MENTAL WELLBEING (SF36)**				
**Baseline**				
n	204	173	173	550
Number missing	1	1	0	2
Proportion missing	0.49	0.57	0	0.36
Mean (SD)	30.29 (14.02)	31.88 (13.98)	30.15 (15.14)	30.75 (14.36)
**12 weeks**				
n	132	140	115	387
Number missing	73	33	58	164
Proportion missing	35.61	18.97	33.53	29.71
Mean (SD)	37.44 (14.95)	37.56 (14.02)	34.12 (15.65)	36.50 (14.88)
**24 weeks**				
n	121	109	88	318
Number missing	84	65	85	234
Proportion missing	40.98	37.36	49.13	42.39
Mean (SD)	35.97 (13.68)	36.38 (14.22)	38.43 (15.19)	36.79 (14.29)
**PHYSICAL WELLBEING (SF36)**				
**Baseline**				
n	204	173	173	550
Number missing	1	1	0	2

Proportion missing	0.49	0.57	0	0.36
Mean (SD)	47.06 (8.84)	46.69 (10.62)	48.02 (11.15)	47.25 (10.17)
**12 weeks**				
n	132	141	115	388
Number missing	73	33	58	164
Proportion missing	35.61	18.97	33.53	29.71
Mean (SD)	48.37 (10.02)	47.72 (11.08)	47.06 (11.14)	47.74 (10.73)
**24 weeks**				
n	121	109	88	318
Number missing	84	65	85	234
Proportion missing	40.98	37.36	49.13	42.39
Mean (SD)	49.20 (10.11)	48.09 (11.59)	48.72 (10.96)	48.69 (10.85)
**DEPRESSION (HADS)**				
**Baseline**				
n	205	173	173	551
Number missing	0	1	0	1
Proportion missing	0	0.57	0	0.18
Mean (SD)	9.51 (4.42)	9.42 (5.00)	9.46 (4.84)	9.47 (4.73)
**12 weeks**				
n	132	142	116	390
Number missing	73	32	57	162
Proportion missing	35.61	18.39	32.95	29.35
Mean (SD)	7.58 (5.07)	7.59 (4.68)	8.28 (4.75)	7.79 (4.83)
**24 weeks**				
n	123	109	89	321
Number missing	82	65	84	231
Proportion missing	40	37.36	48.55	41.85
Mean (SD)	7.72 (4.44)	7.46 (4.72)	7.02 (5.46)	7.44 (4.83)
**ANXIETY (HADS)**				

**Baseline**				
n	205	173	173	551
Number missing	0	1	0	1
Proportion missing	0	0.57	0	0.18
Mean (SD)	10.58 (5.12)	9.75 (5.20)	10.31 (5.44)	10.23 (5.25)
**12 weeks**				
n	132	142	116	390
Number missing	73	32	57	162
Proportion missing	35.61	18.39	32.95	29.35
Mean (SD)	8.79 (5.21)	8.77 (5.18)	9.59 (5.55)	9.02 (5.30)
**24 weeks**				
n	123	109	89	321
Number missing	82	65	84	231
Proportion missing	40	37.36	48.55	41.85
Mean (SD)	9.10 (5.13)	8.71 (5.11)	7.83 (5.78)	8.61 (5.32)

Table 5. Odds ratios for Secondary Outcome Measures: Social Functioning (OPI), Alcohol Use Disorders (AUDIT) physical and mental wellbeing (SF-36), quality of life (EQ-5D) depression and anxiety (HADS).

	Treatment group difference estimate	Lower confidence limit	Upper confidence limit	p-value
**Mean differences in AUDIT across groups**				
12 weeks CM Attendance v CM Abstinence	0.42	-1.17	2.02	0.605
12 weeks TAU v CM Abstinence	0.45	-1.22	2.11	0.600
12 weeks TAU v CM Attendance	0.02	-1.62	1.67	0.977
24 weeks CM Attendance v CM Abstinence	-0.99	-2.68	0.69	0.247
24 weeks TAU v CM Abstinence	-0.17	-1.93	1.60	0.854
24 weeks TAU v CM Attendance	0.83	-0.97	2.63	0.366
**Mean differences in OPIATE TREATMENT INDEX Social Functioning across groups**				
12 weeks CM Attendance v CM Abstinence	0.24	-1.06	1.54	0.715
12 weeks TAU v CM Abstinence	-0.08	-1.44	1.28	0.912
12 weeks TAU v CM Attendance	-0.32	-1.65	1.02	0.640
24 weeks CM Attendance v CM Abstinence	0.32	-1.07	1.70	0.655
24 weeks TAU v CM Abstinence	0.10	-1.36	1.56	0.889
24 weeks TAU v CM Attendance	-0.21	-1.70	1.28	0.781
**Mean differences in SF36 MCS across groups**				
12 weeks CM Attendance v CM Abstinence	-1.08	-4.45	2.29	0.529
12 weeks TAU v CM Abstinence	-2.49	-5.98	1.00	0.162
12 weeks TAU v CM Attendance	-1.41	-4.89	2.07	0.427
24 weeks CM Attendance v CM Abstinence	-1.76	-5.29	1.77	0.330
24 weeks TAU v CM Abstinence	1.19	-2.49	4.87	0.526
24 weeks TAU v CM Attendance	2.95	-0.80	6.69	0.123
**Mean differences in SF36 PCS across groups**				
12 weeks CM Attendance v CM Abstinence	-0.35	-2.33	1.62	0.727

12 weeks TAU v CM Abstinence	-2.06	-4.13	0.02	0.052
12 weeks TAU v CM Attendance	-1.70	-3.75	0.35	0.103
24 weeks CM Attendance v CM Abstinence	-0.90	-3.01	1.21	0.402
24 weeks TAU v CM Abstinence	-0.75	-2.98	1.48	0.511
24 weeks TAU v CM Attendance	0.15	-2.11	2.42	0.895
**Mean differences in HADS depression across groups**				
12 weeks CM Attendance v CM Abstinence	0.08	-1.05	1.22	0.885
12 weeks TAU v CM Abstinence	0.69	-0.48	1.87	0.249
12 weeks TAU v CM Attendance	0.61	-0.56	1.78	0.309
24 weeks CM Attendance v CM Abstinence	-0.17	-1.36	1.03	0.784
24 weeks TAU v CM Abstinence	-0.62	-1.86	0.63	0.331
24 weeks TAU v CM Attendance	-0.45	-1.72	0.82	0.487
**Mean differences in HADS anxiety across groups**				
12 weeks CM Attendance v CM Abstinence	0.46	-0.84	1.76	0.485
12 weeks TAU v CM Abstinence	0.33	-1.00	1.67	0.624
12 weeks TAU v CM Attendance	-0.13	-1.46	1.21	0.851
24 weeks CM Attendance v CM Abstinence	0.48	-0.87	1.83	0.482
24 weeks TAU v CM Abstinence	-0.81	-2.21	0.58	0.252
24 weeks TAU v CM Attendance	-1.30	-2.72	0.12	0.073

Table 6. ARM-5 – Therapeutic Alliance

	CM Abstinence n=205	CM Attendance n = 174	TAU n= 173	Total n = 552
**ARM-5 mean score**				
**Week 4**				
n	91	84	36	211
Number missing	114	90	137	341
Proportion missing	55.61	51.72	79.19	61.78
Mean (SD)	6.54 (0.73)	6.71 (0.63)	6.26 (1.09)	6.56 (0.78)
**Week 8**				
n	63	69	28	160
Number missing	142	105	145	392
Proportion missing	69.27	60.34	83.82	71.01
Mean (SD)	6.60 (0.78)	6.59 (0.69)	6.16 (1.01)	6.52 (0.80)
**Week 12**				
n	123	126	91	340
Number missing	82	48	82	212
Proportion missing	40.00	27.59	47.40	38.41
Mean (SD)	6.43 (0.96)	6.27 (1.27)	5.88 (1.30)	6.23 (1.19)
