# Supplementary material for: Using a pragmatically adapted, low-cost contingency management intervention to promote heroin abstinence in individuals undergoing treatment for heroin use disorder in UK drug services (PRAISE): a cluster randomised trial
Source: BMJ Open. 2021 Jul 1;11(7):e046371. doi: 10.1136/bmjopen-2020-046371 (PMC8252884; doi:10.1136/bmjopen-2020-046371)
Supplement: Author's manuscript [file bmjopen-2020-046371.draft_revisions.pdf]

BMJ Open is committed to open peer review. As part of this commitment we make the peer review history of every article we publish publicly available.

When an article is published we post the peer reviewers' comments and the authors' responses online. We also post the versions of the paper that were used during peer review. These are the versions that the peer review comments apply to.

The versions of the paper that follow are the versions that were submitted during the peer review process. They are not the versions of record or the final published versions. They should not be cited or distributed as the published version of this manuscript.

BMJ Open is an open access journal and the full, final, typeset and author-corrected version of record of the manuscript is available on our site with no access controls, subscription charges or pay-per-view fees (http://bmjopen.bmj.com).

If you have any questions on BMJ Open's open peer review process please email info.bmjopen@bmj.com

BMJ Open

Using a pragmatically adapted, low-cost, contingency management intervention to promote heroin abstinence in individuals undergoing treatment for opioid substance use disorder in UK drug services (PRAISE): a cluster randomised trial

Journal:	*BMJ Open*
Manuscript ID	bmjopen-2020-046371
Article Type:	Original research
Date Submitted by the Author:	29-Oct-2020
Complete List of Authors:	Metrebian, Nicola; King's College London Institute of Psychiatry Psychology and Neuroscience, Addiction<br>Weaver, Timothy; Middlesex University, Department of Mental Health & Social Work<br>Goldsmith, Kimberley; King's College London, Biostatistics; Institute of Psychiatry, King's College London<br>Pilling, Stephen; University College London, Centre for Outcomes, Research & Effectiveness<br>Hellier, Jennifer; King's College London Institute of Psychiatry Psychology and Neuroscience, Biostatistics and Health Informatics<br>Pickles, Andrew; King's College London Institute of Psychiatry Psychology and Neuroscience, Biostatistics and Health Informatics<br>Shearer, James; King's College London Institute of Psychiatry Psychology and Neuroscience, Health Services and Population Research<br>Byford, Sarah; King's College London Institute of Psychiatry Psychology and Neuroscience, Health Service & Population Research Department.<br>Mitcheson, Luke; South London and Maudsley NHS Foundation Trust<br>Bijral, Prun; Change, Grow, Live, Management Offices<br>Bogdan, Nadine; Essex Partnership University NHS Foundation Trust<br>Bowden-Jones, Owen; Central and North West London NHS Foundation Trust<br>Day, Edward; Birmingham and Solihull Mental Health NHS Foundation Trust<br>Dunn, John; Camden and Islington NHS Foundation Trust<br>Glasper, Anthony; Sussex Partnership NHS Foundation Trust, Hastings Substance Misuse Service<br>Finch, Emily; South London and Maudsley NHS Foundation Trust<br>Forshall, Sam; Avon and Wiltshire Mental Health Partnership NHS Trust<br>Akhtar, Shabana; Birmingham and Solihull Mental Health NHS Foundation Trust<br>Bajaria, Jalpa; Essex Partnership University NHS Foundation Trust, R&D; National Institute for Health Research, CRN North West London<br>Bennett, Carmel; Birmingham and Solihull Mental Health NHS Foundation Trust<br>Bishop, Elizabeth; University College London<br>Charles, Vikki; King's College London Institute of Psychiatry Psychology and Neuroscience, Addiction

Davey, Clare; Avon and Wiltshire Mental Health Partnership NHS Trust
Desai, Roopal; King's College London Institute of Psychiatry Psychology and Neuroscience, Addiction
Goodfellow, Claire; University of Glasgow MRC/CSO Social and Public Health Sciences Unit, ; University of Glasgow, MRC / CSO Social and Public Health Sciences Unit
Haque, Farjana; King's College London Institute of Psychiatry Psychology and Neuroscience, Addiction
Little, Nicholas; University College London, Outcomes, Effectiveness
McKechnie, Hortencia; King's College London Institute of Psychiatry Psychology and Neuroscience, Addiction; Imperial College London Faculty of Medicine, Mental Health
Mosler, Franziska ; King's College London Institute of Psychiatry Psychology and Neuroscience, Addictions
Morris, Jo; Avon and Wiltshire Mental Health Partnership NHS Trust, Research & Development
Mutz, J; Imperial College London, Epidemiology and Biostatistics
Pauli, Ruth; University of Birmingham
Poovendran, Dilkushi; Imperial College London Faculty of Medicine
Phillips, Elizabeth; Essex Partnership University NHS Foundation Trust
Strang, John; King's College London Institute of Psychiatry Psychology and Neuroscience, Addictions

Keywords: Substance misuse < PSYCHIATRY, Clinical trials < THERAPEUTICS, Adult psychiatry < PSYCHIATRY

SCHOLARONE™ Manuscripts

I, the Submitting Author has the right to grant and does grant on behalf of all authors of the Work (as defined in the below author licence), an exclusive licence and/or a non-exclusive licence for contributions from authors who are: i) UK Crown employees; ii) where BMJ has agreed a CC-BY licence shall apply, and/or iii) in accordance with the terms applicable for US Federal Government officers or employees acting as part of their official duties; on a worldwide, perpetual, irrevocable, royalty-free basis to BMJ Publishing Group Ltd ("BMJ") its licensees and where the relevant Journal is co-owned by BMJ to the co-owners of the Journal, to publish the Work in this journal and any other BMJ products and to exploit all rights, as set out in our licence.

The Submitting Author accepts and understands that any supply made under these terms is made by BMJ to the Submitting Author unless you are acting as an employee on behalf of your employer or a postgraduate student of an affiliated institution which is paying any applicable article publishing charge ("APC") for Open Access articles. Where the Submitting Author wishes to make the Work available on an Open Access basis (and intends to pay the relevant APC), the terms of reuse of such Open Access shall be governed by a Creative Commons licence – details of these licences and which Creative Commons licence will apply to this Work are set out in our licence referred to above.

Other than as permitted in any relevant BMJ Author's Self Archiving Policies, I confirm this Work has not been accepted for publication elsewhere, is not being considered for publication elsewhere and does not duplicate material already published. I confirm all authors consent to publication of this Work and authorise the granting of this licence.

For peer review only

Using a pragmatically adapted, low-cost, contingency management intervention to promote heroin abstinence in individuals undergoing treatment for opioid substance use disorder in UK drug services (PRAISE): a cluster randomised trial

Nicola Metrebian [a*], Timothy Weaver [b,c*], Kimberley Goldsmith [a], Prof Stephen Pilling [d], Jennifer Hellier [a], Prof Andrew Pickles [a], James Shearer [a], Prof Sarah Byford [a], Luke Mitcheson [f], Prun H. Bijral [h], Nadine Bogdan [i], Owen Bowden-Jones [j], Edward Day [k], John Dunn [l], Anthony Glasper n, Emily Finch, Sam Forshall[m], Shabana Akhtar[k], Jalpa Bajaria[j], Carmel Bennett[k], Ellie Bishop[d], Vikki Charles[a], Clare Davey[m], Roopal Desai[a], Claire Goodfellow[d], Farjana Haque[a], Nicholas Little[d], Hortencia McKechnie[b,a], Franziska Mosler[a], Jo Morris[m], Julian Mutz[a], Ruth Pauli[k], Dilkushi Poovendran [b], Elizabeth Phillips [l] and Prof Sir John Strang [a,f]

* Joint first authors.

on behalf of the Contingency Management Programme Team

a King's College London, National Addiction Centre, Institute of Psychiatry, Psychology & Neuroscience, London, UK
b Imperial College London, London, UK
c Middlesex University, London, UK
d University College London, London, UK
e King's College London, Institute of Psychiatry, Psychology & Neuroscience, London, UK
f South London and Maudsley NHS Foundation Trust, London, UK
g Dudley & Walsall Mental Health Partnership Trust, Dudley, UK
h Change, Grow, Live Charity, Management Offices, London, UK
i South Essex Partnership NHS Foundation Trust, Essex, UK
j Central and North West London NHS Foundation Trust, London, UK
k Birmingham & Solihull Mental Health NHS Foundation Trust, Birmingham, UK
l Camden & Islington NHS Foundation Trust, London, UK
m Avon & Wiltshire Mental Health Partnership NHS Trust, Bristol, UK
n Sussex Partnership NHS Foundation Trust, Brighton, UK

Corresponding author: Dr Nicola Metrebian, Addictions Department, Institute of Psychiatry, Psychology and Neuroscience, King's College London, 4 Windsor Walk, Denmark Hill, London SE5 8AA. Nicola.metrebian@kcl.ac.uk

Trial registration ISRCTN 01591254

For peer review only

For peer review only - http://bmjopen.bmj.com/site/about/guidelines.xhtml

Abstract

Introduction: Most individuals treated for opioid use disorder receive opioid agonist treatment (OAT) (methadone or buprenorphine). However, OAT is associated with high attrition and persistent, occasional heroin use. There is some evidence for the effectiveness of contingency management (CM), a behavioural intervention involving modest financial incentives, in encouraging drug abstinence when applied adjunctively with OAT. UK drug treatment services have a minimal track record of applying CM and limited resources to implement it. We aimed to assess a CM intervention pragmatically adapted for ease of implementation in UK drug treatment services to achieve heroin-abstinence among individuals receiving OAT.

Design: Cluster randomised controlled trial

Setting and participants: 552 participants (of target 660) were enrolled from 34 clusters (drug treatment clinics) in England between November 2012 and October 2015.

Interventions: Clusters were randomly allocated 1:1:1 to OAT plus 12 x weekly appointments with either: i) CM targeted at opiate-abstinence at appointments (CM Abstinence); ii) CM targeted at on-time attendance at appointments (CM Attendance); or, iii) no CM (Treatment As Usual; TAU). Modifications included monitoring behaviour weekly and fixed-incentives schedule.

Measurements: Primary outcome was heroin-abstinence measured by heroin-free urines (weeks 9-12). Heroin-abstinence was measured again 12 weeks after discontinuation of CM (21-24 weeks).

Results: CM Attendance had 2.1 times the odds of a heroin-negative urine in weeks 9-12 (95% CI:1.1 to 3.9,p=0.030) compared to TAU. CM Abstinence had 1.6 times the odds of a heroin-negative urine compared to TAU (95% CI:0.85 to 3.01,p=1.46). CM Abstinence was similar to CM Attendance with 1.3 times the odds of a heroin-negative urine (95% CI:0.68 -2.41,p=0.438). Reductions in heroin use were not sustained in any group at 21-24 weeks.

Conclusions: Using a pragmatically adapted CM intervention for routine use in UK drug treatment services was moderately effective in encouraging heroin-abstinence compared with no CM.

The trial is prospectively registered, number ISRCTN 01591254.

Funding: NIHR (RP-PG-0707-10149)

Strengths and Limitations

- To our knowledge, this is the first clinical trial to examine the effectiveness of contingency management in promoting heroin-abstinence among those in opioid agonist treatment in the UK.
- This study is a large, cluster randomised trial conducted at 34 drug treatment services in England
- Contingency management, as developed in the US, was pragmatically adapted for ease of implementation in resource poor UK substance use treatment settings

For peer review only

- Adaptions included using drug service staff to deliver all aspects of the CM intervention (as opposed to CM specialists); staff were trained, supported by a CM Handbook and regular supervision
- Illicit drug use was monitored through urine drug screens (UDS) once a week rather than more frequently using a fixed rather than escalating schedule,

Introduction

Opioid agonist treatment (OAT) (methadone and buprenorphine) is recognised globally as a clinically[1,2] and cost-effective[2,3] treatment for opiate use disorder. However, its effectiveness is often undermined by high attrition associated with relapse into illicit drug use.[4] In the United Kingdom (UK), the National Institute for Health and Care Excellence (NICE) recommends psychological therapies (including contingency management (CM)) be offered alongside OAT[2] to support behaviour change.

CM is a behavioural intervention based on the principles of operant conditioning[5], delivered as a time-limited adjunct to existing evidence-based treatments (such as OAT) to amplify patient benefit. It involves providing positive reinforcement (usually monetary vouchers or prizes) contingent on achieving pre-specified behaviour consistent with treatment goals.[6,7]

Evidence, primarily from US trials, shows that CM is an effective adjunct to substance use treatment in encouraging abstinence from drug use.[8,9,10,11,12] However, there is weaker evidence for the effectiveness of CM in encouraging abstinence from illicit opiates among those receiving OAT.[9,12, 13,14]

UK guidance based on the evidence at the time,[8,9] recommended CM should be utilised in UK drug treatment to target the reduction of illicit drug use and encourage attendance at appointments.[2,15] However, UK drug treatment services had little experience of applying CM, reduced treatment budgets and limited staff capacity; conditions likely to impede implementation of CM.[6] We aimed to assess a CM intervention pragmatically adapted for ease of implementation in routine UK practice. We have previously demonstrated that low-cost CM can affect short-term behaviour change of improved adherence to a hepatitis B vaccination schedule[16] achieving clear long-term health economic benefit.[17] The trial reported here focused on more complex and longer-term behaviour change. Specifically, we assessed the effectiveness of two different 12-week CM schedules targeting (i) opiate-abstinence or (ii) attendance at clinical appointments, providing immediate positive reinforcement through vouchers delivered by trained staff, in achieving heroin-abstinence among individuals receiving OAT in community drug-treatment settings.

Methods

Study design and setting

We employed a pragmatic cluster randomised controlled trial design described elsewhere.[18] Thirty-four drug treatment clinics (clusters) in England (NHS: London, Birmingham, Sussex, Essex, Bath & Bristol, Dudley & Walsall; Non-NHS London, Hertfordshire and Birmingham) were randomly

For peer review only - http://bmjopen.bmj.com/site/about/guidelines.xhtml

allocated to one of three conditions (described below) and tasked with recruiting a cluster-sample of 22 participants. Clinics were eligible if they provided OAT (methadone or buprenorphine), weekly clinical appointments and received enough OAT referrals to meet recruitment targets.

Within each cluster, participants received the same allocated condition thus minimising the risk of contamination between intervention and control arms.

The trial tested the following research hypotheses:

- CM (positive reinforcement targeted at treatment attendance) will increase abstinence from street-heroin when compared to treatment as usual (TAU) in which no positive reinforcement is offered.
- CM (positive reinforcement targeted at the provision of opiate-negative urine samples at treatment appointments) will increase abstinence from street-heroin when compared to TAU in which no positive reinforcement is offered.
- Differences in the type of CM schedules will be associated with differences in heroin-abstinence.

Having both CM interventions allowed us to assess whether any benefit (abstinence) derives from a direct effect of CM on abstinence as the behavioural target, or is a general benefit resulting from CM-stimulated improved attendance at clinical appointments (and possibly consequent improvements in treatment retention).

Findings from parallel economic and process evaluations are to be reported separately.

Participants

Eligible patients were aged 18 and above; seeking a new episode of OAT; regular users of street heroin (ie. self-reported use 15/preceding 30 days [at least 3 days a week], and all [minimum 1] urine drug screens (UDS) in previous month positive for opiates); meeting ICD-10 criteria for opiate dependence; willing to receive 12 week CM intervention; at liberty to participate in the study for 24 weeks; and willing and able to provide informed consent. We excluded patients if they: could not read English and required an interpreter to understand a brief oral description of the study; were pregnant or breastfeeding; and/or were referred through the criminal justice pathway and were receiving a community sentence on condition of attending drug treatment.

Randomisation and masking

Each cluster (drug clinic) was randomly assigned to OAT plus 12 x weekly clinical appointments with either:

i) CM Abstinence: positive reinforcement contingent upon opiate-abstinence monitored through UDS undertaken at each weekly appointment;

ii) CM Attendance: positive reinforcement contingent upon attendance on time; or,

iii) no CM (TAU; control condition).

For peer review only - http://bmjopen.bmj.com/site/about/guidelines.xhtml

Randomisation was undertaken independently by the King's Clinical Trials Unit. Clusters were assigned to treatments using random permuted blocks within type of service provider strata (NHS or non-NHS) using a block length of 3 in a 1:1:1 allocation ratio. Laboratory personnel who completed the urinalysis and the statistician analysing primary outcome data were all blinded to treatment allocation.

Interventions

OAT was delivered in line with existing service protocols at all clinics and included weekly clinical appointments of up to 50 minutes with a named drug worker. Attending the service to obtain a prescription did not constitute attendance. The CM interventions (described below) were delivered adjunctively.

In our trial, CM, as developed in the US, was pragmatically adapted for use in resource poor UK drug treatment services to ease future implementation. Key CM principles were retained; targeting a clearly defined behaviour, regular monitoring of that behaviour and providing an immediate reinforcer ensured that a clear contingent relationship was made between the positive behaviour and the reinforcer. The reinforcer was withheld if the target behaviour was not achieved. Adaptions included: (a) training drug workers to deliver CM (as opposed to CM specialists); b) monitoring illicit drug use through weekly UDS (rather than more frequently) to fit routine UK practice of taking UDS once a week at the start of treatment and less frequently thereafter; and c) using a fixed rather than escalating schedule, as drug service staff had previously reported the use of escalating schedules to be too difficult.[19] CM consisted of positive reinforcement - verbal praise and fixed value £10 supermarket voucher at each appointment. Reasoning that anything lower would be unlikely to encourage participants to attend while escalation of the value would increase the costs of the intervention beyond most services budget.

'*CM Abstinence*': Reinforcement was conditional on attendance at weekly appointments and (a) during weeks 1-4 (priming weeks) providing a UDS whatever the result and (b) during weeks 5-12 providing an opiate-negative UDS. Instant UDS tests were completed at the start of each appointment. Staff provided the reinforcer immediately the target behaviour was achieved. Priming ensured participants gained experience of the reinforcer, while also addressing practitioner concerns about the difficulty in achieving abstinence during contemporaneous titration up to a stable dose.
'*CM Attendance':* Eligibility to receive the reinforcement was conditional solely on on-time attendance at the appointment (within 15 minutes of the scheduled appointment time).
Control: Participants received treatment as usual (TAU) (OAT plus 12 x weekly appointments) with no CM.

The CM interventions ceased after week 12, at which point the frequency of appointments reverted to usual care for each service (ie. Varied depending on drug service and patient needs).

Training & supervision

All staff providing clinical appointments received training on trial procedures. At CM sites, staff received bespoke one-day training on the principles and practice of CM (including simulation, role play). Training was supported by a CM Handbook designed for UK services but which drew on international evidence and practice models.[20,21]

For peer review only - http://bmjopen.bmj.com/site/about/guidelines.xhtml

CM was delivered in the first 5-10 minutes of the appointment and audio recorded. A CM adherence measure was developed from measures available at the time[22] but adapted to assess key CM competencies in UK services settings. Audio recordings were used to rate the scale which comprised ten 4-point Likert scale items. Adherence was scored as poor (<33%), adequate (33-66%), or good (>66%). The scale achieved good interrater reliability (k=0.63;p<0.001).

Evidence suggests training alone is unlikely to change health professional behaviour unless supported by effective supervision.[23] Group supervision sessions were provided to staff at intervention sites throughout the trial. The drug service psychologist/senior practitioner provided local supervision after receiving training in CM supervision. Audio recordings of CM delivery were available to local supervisors to provide feedback to staff. In turn, research team psychologists used recordings of supervision sessions to support their supervision of local supervisors.

Research Assessments

Consenting participants completed a research interview (and were reimbursed £20 for time and travel) before enrolment into the trial and again at 12-and 24-weeks post enrolment. All participants were asked to provide (research) UDS weekly between weeks 9-12 and 21-24. Weekly "urine collection clinics" were established by the research team at each clinic (participants were reimbursed for travel) to enable participants to provide UDS outside of appointments. Participants' attendance at appointments, receipt of CM vouchers and provision of urines for CM and research purposes were recorded.

Outcomes

The primary outcome measure of heroin-abstinence was a once-weekly binary positive/negative heroin-UDS collected at weeks 9-12 for laboratory drug testing.

Secondary outcome measures included retention in treatment, self-reported illicit-drug use in the last 30 days,[24] alcohol use (Alcohol Use Disorders (AUDIT)),[25] social functioning (Opiate Treatment Index (OTI)),[26] physical and mental health status (Short Form-36 (SF-36)),[27] and depression and anxiety (Hospital Anxiety and Depression Scale (HADS)),[28] obtained by researchers in face to face interviews with patients at baseline, 12 and 24 weeks. Confidential patient ratings of therapeutic alliance with their health-professional were recorded at 4, 8 and 12-weeks post-enrolment (ARM5).[29] A single item measure of delay-discounting[33] and a motivation measure for drug abstinence[35] were ancillary outcome measures and reported elsewhere.

The Adult Service Use Schedule (AD-SUS) adapted for drug users,[29] health-related quality of life (EQ-5D-3L),[31] and the WHO Health and Work Performance Questionnaire[32] were measured for health economic analysis. Findings from the health economic analysis and parallel process evaluation are to be reported separately.

Sample size calculation

More detail is provided in the protocol.[18] Lussier et al [8] estimated a mean weighted effect size of 0.39 from three studies with an opiate-use outcome measure (CM v control). For a two-sided test, alpha = 0.05, 5% attrition, 80% power to detect an effect size of 0.39, 111 participants per group were required. Accounting for clustering by clinic, this was inflated to 220 participants per arm

For peer review only - http://bmjopen.bmj.com/site/about/guidelines.xhtml

(assuming an intra-class correlation of 0.05, 20 participants per cluster and 11 clusters per intervention leading to a design effect of 1.95.) Clinics were initially advised to recruit >20 participants each (33 x 20 = 660 in total). As the trial progressed the calculation was adjusted to account for 10% attrition. Clusters still recruiting were asked to increase recruitment to 22 participants.

Statistical analysis

Statistical analysis was carried out using Stata v14/15 according to the intention-to-treat principle. Variables were summarised using mean/standard deviation, median/quartiles, or frequencies/proportions as appropriate.

Refusal and non-attendance UDS were coded as positive (positive assumption) but results missing for other reasons (e.g. discharged from treatment, in hospital or in prison) were initially left missing. This positive assumption is commonly used in clinical practice and in studies of CM and was used in our previous trial.[35]

These responses were the dependent variables in a mixed effects logistic regression model (9-12 weeks and 21-24 weeks in separate models), with trial arm, the clinic type stratification factor and week dummy variables as fixed-effects, and random intercepts for participants nested within clinic. Results are presented both from fitting a model fitted to data where 1) the positive assumption has been applied, but no further imputation has taken place, 2) further missing data were imputed (100 datasets) and estimates obtained using Rubin's rules. The imputation model included clinic, trial arm, NHS stratification factor, gender, heroin use at 12 and 24 weeks, and baseline measures including frequency of heroin use, AUDIT score, OTI social functioning score anxiety and depression, SF36 physical and mental component scores, and delay discounting, in addition to the four binary weekly urine sample measures. Moderation by type of opioid replacement (methadone or buprenorphine), NHS clinic versus non-NHS and the Charlson index of multiple drug use was tested using the non-imputed data by adding treatment by moderator interaction terms each separately to the model described.

Differences in secondary continuous outcomes between the groups were estimated using linear mixed effects models with the 12 and 24 week outcomes as dependent variables, the same fixed and random effects as for the primary outcome, plus time point, treatment by time interaction, and baseline values of the outcome where applicable as predictors. For the self-report binary drug use variables, marginal logistic generalised estimating equation (GEE) regression models were fitted (to estimate marginal instead of potentially inflated conditional effects from mixed models, which were also fitted to compare),[36] with the same variables as the linear mixed models and with clinic as a clustering variable. Missing data were not imputed.

Retention in the 12-week treatment programme was coded as weeks attended up to the point of continuous future non-attendance. Retention was compared between the groups using: 1) Fisher's exact test for retention in the trial overall, 2) a logistic GEE model for binary retention in opioid treatment at 12 weeks (with exchangeable correlation structure and 1000 repetition bootstrap standard errors), and 3) Kaplan-Meier and Cox regression analysis for time until continuous

For peer review only - http://bmjopen.bmj.com/site/about/guidelines.xhtml

treatment non-attendance, with trial group and NHS stratification factor as predictors and clinic as a clustering variable.

Patient and Public Involvement.

A Service User Research Advisory Group (SURAG) was established before the trial commenced, comprising 14 service users with direct experience of receiving OAT. The group met once every six months to provide advice on the design, conduct and progress of the research. The SURAG helped significantly to make the trial procedures acceptable and aided implementation.

Results

Sample

Between September 2012 and September 2015, we randomly allocated 34 clinics (clusters) (of 61 considered) to three treatments (Figure 1; and see Supplementary material). Of the 34 clinics, all provided OAT, the majority were NHS (62%) and just under one half were within London (47%). Figure 1 shows that across 34 clusters, 789 patients were screened for eligibility, of whom 552 (31.8%) were consented and enrolled (between November 2012 and October 2015), a figure that fell short of our target of 660. One cluster did not recruit any participants.

Retention in the trial was high and comparable across trial arms. Eight participants actively withdrew from the trial (CM Abstinence = 4(2%); CM Attendance = 1(1%); and TAU = 3(2%);p=0.50).

Figure 1 - CONSORT diagram

Characteristics

Study participants were broadly representative of patients entering OAT in England (Table 1). They were mostly male (404;73%), white (435;79%) with a mean age of 38.2 years [SD 8.8]. They had first used opiates at the mean age of 23 years [SD 8], first injected at a mean age of 26 years [SD 8] and first received treatment at a mean age of 30 years [SD 8]. They had previously been in opiate treatment a median of 2 times [25th, 75th percentile 1, 4]. Over half had been in prison (291;53%). Fifty-seven per cent were prescribed methadone (315), 43% buprenorphine (237).

Table 1 - Baseline characteristics

Almost all (550;99.6%) self-reported using heroin in the month prior to interview. (Two reporting using non-prescribed pharmaceutical heroin). Three quarters reported using crack (419;76%) while 23% used benzodiazepines (n=129). Half drank alcohol (53%;n=310) and nearly all used tobacco (531;96%).(Tables 4&5)

There was reasonable balance across the three trial arms in terms of baseline socio-demographic, drug use, health, and treatment experience variables. However, there were more women in the CM

For peer review only - http://bmjopen.bmj.com/site/about/guidelines.xhtml

Attendance group, fewer white individuals in the CM Abstinence group and a smaller proportion with insecure housing status in the CM Attendance group.

Attendance

Figure 2 shows that 60% of those receiving CM Abstinence and 59% receiving CM Attendance attended their first appointment on time, compared with 46% of the TAU group. Eleven per cent of participants did not attend any weekly appointments (CM Abstinence = 11%; CM Attendance=7% and TAU=14%).

Figure 2 - Attendance at appointments

Attrition at each week was highest in the TAU group, with the proportion of participants attending appointments falling from 46% to 24% between weeks 1-12. In the CM Abstinence group there was a decline in attendance from 53% (week 4) down to 45% (week 5) with a steady decline thereafter to 33% attending at week 12. Attendance in the CM Attendance group remained consistent with a slight decline from 59% (week 1) to 51% (week 12). Most attendance was on-time (within 15 minutes)(Figure 2).

Retention in 12 weekly appointments

The proportions attending all of the 12-week appointments differed across the groups, with a higher proportion attending in CM Attendance as compared to CM Abstinence and TAU groups (56% vs 39% and 30%). CM Attendance had 3.1 times the odds of full attendance as compared to TAU (CI 2.0 to 4.6;p<0.001). CM Abstinence had 1.5 times the odds of attending fully as compared to TAU (CI 0.9 to 2.4;p=0.099).

Participants in the CM Attendance group attended for longer than those in the CM Abstinence group or TAU group. Compared to participants in the CM Attendance group, the CM Abstinence group had 1.7 times the risk of dropping out of the appointments before week 12 (95% hazard ratio CI 1.2 to 2.3;p=0.002), while participants in the TAU group had 1.9 times the risk (95% hazard ratio CI 1.5 to 2.5;p<0.001)(Figure 3).

Figure 3 - Time to continuous appointment non-attendance

Abstinence from heroin at 12 weeks

Of the 2208 scheduled UDS (552 participants x 4 urines for weeks 9-12), 601 (27%) were not obtained due to non-compliance (refusal or DNA) and were imputed as positive (positive assumption), with the greatest number of these being in the TAU group. Additionally, 454 (21%) were treated as missing, including: 260 (12%) not obtained due to unable to contact, 77 (3%) not obtained due to clinic error, 15 (1%) unable to provide a sample, 55 (3%) in prison (47) in hospital

For peer review only - http://bmjopen.bmj.com/site/about/guidelines.xhtml

(8), and for 10 (0.5%) the reason was missing. The percentage of UDS results missing by week and trial arm are shown in Table 2.

Table 2 Heroin – negative & positive UDS results

Results from the analyses with and without imputation were similar (Table 3), so results from imputed data are discussed. Participants in the CM Attendance group had 2.1 times the odds of a heroin-negative urine (95% CI 1.1 to 3.9;p=0.030) as compared to those in the TAU group. The CM Abstinence group had 1.6 times the odds of a heroin-negative urine compared to the TAU group (CI 0.9 to 3.0;p=0.146). There was little difference between the CM abstinence and CM attendance groups (OR 1.29; CI:0.68 to 2.41;p=0.438). Figure 4 shows the observed (dashed) and model predicted (solid) expected probabilities of a heroin-negative urine over the four weeks of the primary outcome period. Based on estimated proportions from model (1) in Table 3, a higher proportion of those in CM Attendance (62%) or CM Abstinence (57%) provided a negative-heroin urine sample at week 12 compared to TAU (48%). The intraclass correlations from the non-imputed data were 0.03 (95% CI 0.01 to 0.11) at the clinic level.

Table 3 - Primary outcome: odds of heroin-negative urine over weeks 9 to 12 & 21-24

Figure 4 - Probability of heroin-negative urine applying positive assumption over weeks 9-12 & 21-24.

There was no moderation of trial intervention outcome by medication or treatment setting (Buprenorphine v Methadone (χ^2(2)=1.89;p=.389); NHS or non NHS service (χ^2 (2)=4.96;p=0.084)) or by other or multiple drug use (Charlson Index of multiple drug use (χ^2 (2)=4.93;p=0.085)).

Abstinence from heroin at 24 weeks

There were no differences among groups in heroin-abstinence for weeks 21-24 (Table 4). Figure 4 shows the observed (dashed) and (Model (1) in Figure 4) expected probabilities (solid) of a heroin-negative UDS over the four weeks of the follow-up period (weeks 21-24). The estimated proportions of those providing a negative-heroin UDS at 24 weeks from Model 1 in Figure 4 were similar across groups: CM Abstinence (44%); CM Attendance (45%) and TAU (37%).

Clinical outcomes – Self-reported drug use

Self-reported heroin use reduced in all groups from nearly 100% (99.6%; 550) to 74% (291) at week 12, while the median number of days used reduced from 28 (21,30) to 4 (0,15). Self-reported use of crack and benzodiazepines reduced in the trial population from 76% to 54% and 23% to 16% respectively at 12 weeks. Self-reported alcohol and tobacco use remained similar (56% to 61% and

For peer review only - http://bmjopen.bmj.com/site/about/guidelines.xhtml

96% to 95% respectively). There were no significant differences in these outcomes between groups at 12 weeks (Tables 4 & 5; and see Supplementary material).

Tables 4 & 5 Self-reported drug use

At 24-week follow-up, we found no difference in self-reported illicit drug use between groups. However, there was weak evidence of increased odds of alcohol use in the last 30 days in CM Attendance as compared to CM Abstinence (odds ratio and 95% CI 1.9, 1.0 to 3.5, p = 0.048), and evidence of fewer days using tobacco in the last 30 days in TAU as compared to CM Abstinence (mean difference and 95% CI2.1, -0.3 to -3.9;p=0.024)(Supplementary material). Mixed-effects models gave similar results.

Clinical outcomes – health and social

There were no differences between the groups in clinical outcomes or crime at weeks 12 or 24. A similar therapeutic alliance score was reported by all groups at 4, 8 and 12 weeks (see Supplementary material).

Adherence to CM

Both CM Abstinence and CM Attendance achieved a score of "adequate" adherence to the CM manual. It is important to note these averages were on the lower end of the range of scores in this category (CM Abstinence=38.33%, SD=9.49 (range 24.6% to 56.3%); CM Attendance=44.15%, SD=17.07 (range 16.7% to 68.2%)).

Serious adverse events

There were 25 serious adverse events in 22 participants: CM Abstinence=10 in 9 participants; CM Attendance=8 in 7 participants; TAU=7 in 6 participants. There were 3 deaths (CM Abstinence=1; CM Attendance=2). None were related to the trial intervention.

Discussion

We found the adapted CM intervention either targeted at abstinence or attendance was moderately effective in encouraging heroin-abstinence compared with TAU. CM Attendance was more effective than TAU and CM Abstinence, while not significantly better than TAU, was similar in effect to CM Attendance

The UK's clinical guidance[2,15] which promotes the use of CM, was influenced by systematic reviews and meta-analysis conducted at least ten years ago which showed CM was effective in encouraging abstinence from a range of drugs.[9] Previous reviews examining the effectiveness of CM in promoting opioid-abstinence have been mixed [9,10,11,12,14] with the most recent systematic review and meta-analysis concluding there was no evidence of CM working better than control in encouraging abstinence from non-prescribed opiates during OAT.[14] While our findings contrast with the most recent meta-analysis, the effect size is small and adapting CM for ease of implementation in resource-poor settings may have caused it to lose some of its potential effectiveness.

For peer review only - http://bmjopen.bmj.com/site/about/guidelines.xhtml

When our adapted CM intervention was targeted only at attendance, it had a similar (although slightly larger) effect in encouraging heroin-abstinence compared with CM targeted at abstinence. It has been argued that clinical benefit arises from regular attendance at clinical appointments and overall retention in treatment.[36] Our findings suggest CM can improve attendance (when targeted at it) and that the clinical benefit we observed supports such arguments.

The CM interventions we evaluated retained the main principles of CM but modifications were made to ensure ease of implementation in UK settings with limited treatment budgets.

First, in the UK, resources are limited. Patients receiving OAT attend their service and receive UDS once a week at the start of treatment and less frequently thereafter. Previous studies exploring views of CM among UK drug treatment staff and patients found that staff viewed the US frequency of urine testing to be too resource heavy for the UK settings and service users felt strongly that such a regime would act as a disincentive.[37] Hence, opiate-abstinence was monitored through once-weekly UDS rather than multiple tests per week.

Second, while the CM evidence base demonstrates the efficacy of escalating-value incentives,[8,9,10,11,12,13] we used a fixed-value incentive. In a previous UK study of CM targeted at hepatitis B vaccination, undertaken by the authors, we found escalating schedule was more difficult to implement and for staff to adhere to.[16] Employing escalating schedules might have risked unacceptable levels of protocol violations while also increasing the costs of the intervention, and limiting potential for translation into routine clinical practice. We used £10 vouchers – a figure our SURAG regarded as sufficient to encourage participants to attend and provide a UDS.

Third, it was critical that the CM schedule was acceptable to participants and supported by staff. Our SURAG argued that having to provide opiate-negative UDS would be challenging during titration and staff felt monitoring at this point may be undermining. In response, we introduced an initial 4-week priming phase in the CM Abstinence arm, during which participants received reinforcement for on-time provision of a UDS, irrespective of the result. The use of priming in CM ensures that patients experience receipt of the reinforcer early in treatment.[6] As participants were entering OAT and undergoing dose titration for their methadone/buprenorphine medication, priming provided an opportunity for participants to receive exposure to the incentive without the expectation that they needed to be heroin-abstinent during this period.

Fourth, vouchers were chosen as reinforcers as there is good evidence for their effectiveness[11,12] and costs were relatively low given the once-a-week frequency of reinforcement.

Adherence to CM as set out in the training manual was somewhat better in the CM Attendance group than CM Abstinence group but not significantly so. Movement of trained staff and supervisors away from participating services may well have impacted on adherence. Further exploration of the relationship between adherence and outcomes and the factors which may affect this (such as individual competence or service organisational factors) will help to clarify this.

Although CM has a strong evidence-base in achieving behaviour change, there is weaker evidence for behaviours sustained once the incentives are removed. Previous studies have found differing results with only a small treatment effect following discontinuation of incentives.[11] In this study, the reduction in heroin use was not sustained after discontinuation of CM. We are unable to conclude whether this was due to the cessation of CM or weekly appointments. These appointments were not

For peer review only - http://bmjopen.bmj.com/site/about/guidelines.xhtml

routine practice in most drug services after 12 weeks but considered good clinical practice in the first 12 weeks of treatment.[38] Further research is needed to better understand how to maintain positive behaviour change, including whether the reinforcer needs to be maintained, given intermittently, or tapered. We also need to explore the impact of the cessation, or reduced frequency of appointments after 12 weeks.

While there is a growing body of evidence for CM, it has not been widely implemented in UK drug treatment services. Similarly, the actual adoption and implementation of CM programmes in US community-based treatment programs (including those where most research on CM has been undertaken) is rare,[40] possibly due to limited staff capacity, resources and treatment budgets[6] though there are a some reports of successful implementation.[41]

Adapting CM for ease of implementation in resource-poor settings may have led to a loss of some of its potential effectiveness. Therefore, future research should consider how best to implement CM in routine practice both in UK and international contexts and should consider different models of delivery (including the use of mobile telephone technology).

References

[1] World Health Organization. Guidelines for the Psychosocially Assisted Pharmacological Treatment of Opioid Dependence. 2009 WHO Library

[2] National Institute for Care Excellence (NICE) Drug Misuse 2006

[3] Godfrey C, Stewart D, Gossop M. Economic analysis of costs and consequences of the treatment of drug misuse: 2-year outcome data from the National Treatment Outcome Research Study (NTORS). Addiction 2004; 99;6:697-707NTA 2012

[4] National Treatment Agency for Substance Misuse (NTA) From Access to Recovery: analysing six years of drug treatment data. NTA 2012.

[5] Skinner B.F. The Behavior of Organisms: An Experimental Analysis 1966, Skinner Foundation. Skinner, B. F. (1938). The behavior of organisms: an experimental analysis. Oxford, England: Appleton-Century.

[6] Nancy Petry's Contingency Management for substance abuse treatment: A Guide to Implementing This Evidence- Based practice (2012)

[7] Petry N.M., Alessi S,M., Marx J., Austin M., and Tardif M. Vouchers versus prizes: contingency management treatment of substance abuse in community settings. Journal of Consulting Clinical Psychology, 2005; 73: 1005-14.

[8] Lussier J.P, Heil S.H, Mongeon J.A, Badger G.J, Higgins S.T. A meta-analysis of voucher-based reinforcement therapy for substance use disorders. Addiction 2006; 101: 192-203.

[9] Prendergast M, Podus D, Finney J, Greenwall L, Roll J. Contingency management for treatment of substance use disorders: a meta-analysis. Addiction, 2006; 101: 1546-60.

[10] Higgins S.T, Sigmon S.C. Heil S.H, 2011 Contingency management in the treatment of substance use disorders: trends in the literature. In Ruiz P, Strain E (Eds.) Lowinson and Ruiz's Substance Abuse: A comprehensive Textbook. Lippincott Williams and Wilkins, Philadelphia PA, pp 603-621.

For peer review only - http://bmjopen.bmj.com/site/about/guidelines.xhtml

For peer review only

[11 Davis D.R, Kurti A.N, Skelly J.M. Redner R, White T.J. and Higgins S.T. A review of the literature on contingency management in the treatment of substance misuse disorders 2009-2014. Preventive Medicine. 2016.

[12] European Monitoring Centre for Drugs and Drug Addiction (2016), How can contingency management support treatment for substance use disorders? A systematic review, EMCDDA Papers, Publications Office of the European Union, Luxembourg.

[13] Amato L, Minozzi S, Davoli M, Vecchiet S (2011) Cochrane Database of Systematic Reviews: Psychosocial combined with agonist maintenance treatments versus agonist maintenance treatments alone for treatment of opioid dependence

[14] Ainscough TS, McNeill A, Strang J, Calder R, Brose L... Contingency Management interventions for non-prescribed drug use during treatment for opiate addiction: A systematic review and meta-analysis. 2017. Drug Alcohol Depend. 2017 Sep 1;178:318-339.

[15] National Collaborating Centre for Mental Health. Drug Misuse: psychosocial Interventions. London: British Psychological Society and The Royal College of psychiatrists: (2008).

[16] Weaver T, Metrebian N, Hellier J, Pilling S, Charles V, Little N, Poovendran D, Mitcheson L, Ryan F, Bowden-Jones O, Dunn J, Glasper A, Finch E and Strang J. Use of contingency management incentives to improve completion of hepatitis B vaccination in people undergoing treatment for heroin dependence : a cluster randomised trial. The Lancet 2014; 384: 153-63.

[17] Rafia R, Dodd P.J, Brennan A, Meier P.S, Hope V.D, Ncube F, Byford S, Tie H, Metrebian N, Hellier J, Weaver T, and Strang J. An economic evaluation of contingency management for completion of hepatitis B vaccination in those on treatment for opiate dependence Addiction.2016:111(9):1616-1627.

[18] Metrebian N, Weaver T, Pilling S, Goldmith K, Hellier J, Pickles A, Byford S, Shearer J et al, Positive Reinforcement targeting Abstinence In Substance misuse (PRAISe): Cluster RCT & Process Evaluation of Contingency Management. NIHR Library. Contemporary Clinical Trials 2018; 71 :124-132

[19] Metrebian N, Weaver T, Pilling S, Goldsmith K, Hellier J, Pickles A, Shearer J, Byford S, Rafia R, Dodd P, and Strang J. Developing a UK Evidence Base for Contingency Management in Addiction Treatment: Incentive-based interventions to improve treatments to reduce drug use and associated harms NIHR Programme Grants for Applied Research Programme. Final Report.

[20] Pilling S, Mitcheson L, Little N, Metrebian N, Weaver T and Strang J. 2012 Clinical Manual for delivering Contingency Management Abstinence & Attendance. University College London, Unpublished.

[21] NTA (2006) Models of Care for the treatment of drug misusers: Update 2006. London: NTA.

[22] Carroll KM, Nich C, Sifry RL, Nuro KF, Frankforter TL, Ball SA, Fenton L & Rounsaville BJ. A General system for evaluating therapist adherence and competence in psychotherapy research in the addictions, Drug and Alcohol Dependence 2000; 1; 57(3):225-38.)

For peer review only - http://bmjopen.bmj.com/site/about/guidelines.xhtml

[23] Herscell et al The role of the therapist training in the implementation of psychosocial treatments: a review and critique with recommendi+ons . Clinical psychology Review 2010; 30(4), 448-466

[24]. Darke, S., Heather, N., Hall, W., Ward, J. & Wodak, A. Estimating drug consumption in opioid users: Reliability and validity of a "recent use" episodes method. British Journal of Addiction, 1991; 86, 1311-1316.

[25] Saunders, J.B., Aasland, O.G., Babor, T.F., de la Fuente, J.R. and Grant, M. Development of the Alcohol Use Disorders Identification Test (AUDIT): WHO collaborative project on early detection of persons with harmful alcohol consumption. II. Addiction, 1993; 88:791-804.

[26]. Darke S, Hall W, Wodak A, Heather N, Ward J. Development and validation of a multidimensional instrument for assessing outcome of treatment among opiate users: the Opiate Treatment Index Addiction; 1992; 87;5:733–74217.

[27]. SF-36 version II. QualityMetric Incorporated

[28]. Zigmond AS, Snaith P. The hospital anxiety and depression scale. Acta Psychiatr Scand. 1983; 67:361–370.

[29] Cahill, T. J, & Stiles, W, Barkham, M, Hardy, G Stone, G & Agnew-Davies, R & Unsworth, G. Two short forms of the Agnew Relationship Measure: the ARM-5 and ARM-12. Psychotherapy Research : Journal of the Society for Psychotherapy Research; 2011; 22: 241-55.

[30] Byford, S., et al., Cost-effectiveness of injectable opioid treatment v. oral methadone for chronic heroin addiction. British Journal of Psychiatry, 2013. 203: p. 341-349.

[31] EuroQol Group. EuroQol—a new facility for the measurement of health-related quality of life. Health Policy. 1990; 16: 199–208

[32] Kessler R, et al The World Health Organisation Health and Work Performance Questionnaire. (HPQ), J. Occupat. Environ. Med. 2003, 45 (2): 156-174.

[33] Madden, G. J., Petry, N. M., Badger, G. J., & Bickel, W. K. (1997). Impulsive and self-control choices in opioid-dependent patients and non-drug-using control participants: Drug and monetary rewards. Experimental and Clinical Psychopharmacology, 1997; 5, 256-262.

[34] Levesque C.S., Williams G.C., Elliot D, Pickering M.A., Bodenhamer B. and Finley P.J. Validating the theoretical structure of the Treatment Self-Regulation Questionnaire (TSRQ) across three different health behaviours Health Education Research 2007; 22(5): 691–702

[35] Strang J, Metrebian N, Lintzeris N, Potts L, Carnwath T, Mayet S, et al. Supervised injectable heroin or injectable methadone versus optimised oral methadone as treatment for chronic heroin addicts in England after persistent failure in orthodox treatment (RIOTT) : a randomised trial. Lancet 2010, 375: 1885-95

[36] F B Hu, J Goldberg, D Hedeker, B R Flay, M A Pentz. Comparison of Population-Averaged and Subject-Specific Approaches for Analyzing Repeated Binary Outcomes, Am J Epidemiol. 1998 Apr 1;147(7):694-703. doi: 10.1093/oxfordjournals.aje.a009511

For peer review only - http://bmjopen.bmj.com/site/about/guidelines.xhtml

[37] Sinclair, J. M. A., Burton, A., Ashcroft, R. & Priebe, S. 2011. Clinician and service user perceptions of implementing contingency management: A focus group study. Drug and Alcohol Dependence., 119, 56-63.

[38] Department of Health and devolved administrations: Drug misuse and dependence: UK Guidelines on clinical management Drug misuse and dependence 2007. Department of Health.

[39] Hartzler B, Lash SJ, Roll JM. Contingency management in substance abuse treatment: A structured review of the evidence for its transportability. Drug and Alcohol Dependence. 2012;122(1):1-10.

[40] Kropp F., Lewis D., Winhusen T. The effectiveness of ultra-low magnitude reinforcers: Findings from a "real-world" application of contingency management. Journal of Substance Abuse Treatment, 2017;72: 111-116.

[41] DePhilippis D, Petry N, Bonn-Miller M, Rosenbach S, Mckay J.R. University of PennsylThe national implementation of Contingency Management (CM) in the Department of Veterans Affairs: Attendance at CM sessions and substance use outcomes, Drug and Alcohol Dependence 2018.185:367-373

Acknowledgements

We would like to thank all the drug service staff and service users who participated in this trial. We also gratefully acknowledge the support and guidance of members of our Trial Steering Committee (Simon Coulton [Chair], Christopher Whiteley, and Soraya Mayet); our Data Management and Ethics Committee (Louise Sell [Chair], Anne R Lingford-Hughes, and Zoe Hoare) and our Service User Research Advisory Group and Anthea Martin for facilitating this group. Also, we would like to acknowledge the guidance and support we received from the late Prof Nancy Petry who collaborated on this trial.

Funding statement

This paper presents independent research funded by the National Institute for Health Research (NIHR) under its Programme Grants for Applied Research Programme (grant reference number RP-PG-0707-10149). The views expressed are those of the authors and not necessarily those of the National Health Service, the NIHR, or the Department of Health. The research was also part funded by the National Institute for Health Research (NIHR) Biomedical Research Centre at South London and Maudsley NHS Foundation Trust and King's College London.

Ethical Review

The PRAISE (Positive Reinforcement targeting Abstinence In Substance misusE) trial was reviewed at the NRES Committee South East Coast-Surrey 12/LO/0910 and received a favourable opinion on the 25/07/2012.

The trial is prospectively registered, ISRCTN 01591254.

Competing interests' statement

For peer review only - http://bmjopen.bmj.com/site/about/guidelines.xhtml

JS, SP and LM have contributed to UK guidelines on the potential role of contingency management in the management of opioid addiction (NICE, 2007; convened by SP, chaired by JS), SP receives funding from NICE for the production of clinical guidelines. JS has chaired the broader-scope pan-UK working group preparing the 2017 and 2007 Orange Guidelines for the UK Departments of Health and Social Care, providing guidance on management and treatment of drug dependence and misuse, including guidance on possible inclusion of contingency management. LM and ED contributed to these guidelines.

JS is a researcher and clinician who, through his university, has worked with various pharmaceutical companies to identify new or improved treatments and his employer (King's College London) has received grants, travel costs and/or consultancy payments from companies including, past 3 years, Indivior, Mundipharma, Camurus, Molteni Farma, Accord. JS has also worked with various drug policy organisations and advisory bodies including the Society for the Study of Addiction (SSA), and the European Monitoring Centre for Drugs and Drug Addiction (EMCDDA).

JS and KG are supported by the National Institute for Health Research (NIHR) Biomedical Research Centre for Mental Health at South London and Maudsley NHS Foundation Trust and King's College London. JS is an NIHR Senior Investigator.

For a fuller account, see JS's web-page at http://www.kcl.ac.uk/ioppn/depts/addictions/people/hod.aspx

NM is involved in research project funded by Pharmaceutical company MundiPharma.

LM has been in receipt of an untied educational grant from Indivior for the ARC study which incorporated the use of CM in the intervention arm. LM is currently involved in Pharmaceutical company (Indivior) funded study – the EXPO trial which will incorporate the use of CM as part of the psychosocial intervention. LM has a paid secondment to PHE to advise on best practice psychological interventions in drug and alcohol treatment. ED has recently been appointed as the government's Drug Recovery Champion.

Authors contribution

Contributors: As the corresponding author – I have had full access to all the data in the study and had final responsibility for the decision to submit for publication.

Concept, original design (JSt, TW, SP, SB); trial administration and management (NM, TW); treatment development, training and supervision (SP, LM); Statistical analysis (KG, JH, AP, JS, SB);, implementation lead medical clinicians (PB, NB, OBJ, ED, JD, AG, EF, SF, GM); data management (NM & FH), data handling and entry (SB, JB, CB, EB, VC, RD, CG, FH, NL, HMK, FM, JM, JM, RP, DP, EP); data interpretation(NM, TW, KG, JSt); First draft manuscript (NM, KG, TW); further work on manuscript (NM, TW, KG, SP, JSt, SB, JS). All authors read and approved the final manuscript.

Data sharing statement

Data collected for the study, including individual participant data and a data dictionary defining each field in the set, will be made available to others. These data will include: deidentified participant data, data dictionary, study protocol, statistical analysis plan, and informed consent form. These data will be available with publication and on application to Dr Nicola Metrebian.

For peer review only - http://bmjopen.bmj.com/site/about/guidelines.xhtml

Access criteria for obtaining data will include approval by Chief Investigator (Prof Sir John Strang), approval of study proposal, and with a signed data access agreement.

For peer review only

For peer review only - http://bmjopen.bmj.com/site/about/guidelines.xhtml

TABLES

For peer review only

For peer review only - http://bmjopen.bmj.com/site/about/guidelines.xhtml

Table 1: Baseline characteristics by treatment group.

		CM Abstinence			CM Attendance			TAU			TOTAL		
		N	Mean / or median %	SD or IQR	N	Mean / or median %	SD or IQR	N	Mean / or median %	SD or IQR	N	Mean / or median %	SD or IQR
**DEMOGRAPHICS**													
Age at Baseline	(years) (mean)	205	38.39	8.76	174	38.02	8.48	173	38.08	9.30	552	38.18	8.83
Gender	male	160	78.05		118	67.82		126	72.83		404	73.19	
	female	45	21.95		56	32.18		47	27.17		148	26.81	
Ethnicity	White	145	70.73		145	83.33		145	83.82		435	78.80	
	Black	15	7.32		5	2.87		13	7.51		33	5.98	
	Asian	23	11.22		11	6.32		8	4.62		42	7.61	
	mixed	18	8.78		13	7.47		6	3.47		37	6.70	
	not done	4	1.95		0	0		1	0.58		5	0.91	
Employment	Employed	13	6.34		27	15.52		22	12.72		62	11.23	
	Unemployed/sickness	190	92.68		146	83.91		145	83.82		481	87.14	
	Student	0	0		0	0		1	0.58		1	0.18	
	House wife/husband	2	0.98		0	0		1	0.58		3	0.54	
	Retired	0	0		0	0		2	1.16		2	0.36	
	Other	0	0		1	0.57		2	1.16		3	0.54	
Normally live with	Partner/spouse	33	16.10		43	24.71		27	15.61		103	18.66	
	Friends	16	7.80		11	6.32		14	8.09		41	7.43	

For peer review only - http://bmjopen.bmj.com/site/about/guidelines.xhtml

	Alone	89	43.41		75	43.10		82	47.40		246	44.57	
	Family	45	21.95		31	17.82		31	17.92		107	19.38	
	Other	21	10.24		14	8.05		19	10.98		54	9.78	
	Unknown	1	0.49		0	0.00		0	0.00		1	0.18	
Accommodation	Owner occupier	22	10.73		13	7.47		8	4.62		43	7.79	
	Rented private	33	16.10		44	25.29		34	19.65		111	20.11	
	Rented (LA, HA)	75	36.59		75	43.10		73	42.20		223	40.40	
	Living with parent	23	11.22		15	8.62		14	8.09		52	9.42	
	B&B/hotel	2	0.98		3	1.72		2	1.16		7	1.27	
	Hostel	28	13.66		12	6.90		10	5.78		50	9.06	
	NFA (living on the	21	10.24		12	6.90		31	17.92		64	11.59	
	Other	0	0		0	0		1	0.58		1	0.18	
	Unknown	1	0.49		0	0		0	0		1	0.18	
Prison	No	95	47.03		80	46.24		81	47.09		256	46.80	
	Yes	107	52.97		93	53.76		91	52.91		291	53.20	
Times on remand		105	6.28	8.69	92	5.27	5.59	88	5.10	7.59	285	5.59	7.45
**DRUG USE HISTORY**													
Age first used opiates	(years)	200	23.39	8.01	169	23.67	7.65	173	23.35	7.48	542	23.46	7.72
Age first regular use	(years)	200	25.30	8.18	173	26.02	7.90	173	26.28	7.92	546	25.84	8.01
Age first injected	(years)	121	26.10	7.85	92	26.51	8.48	108	26.32	7.46	321	26.29	7.88
Age first received help	(years)	193	30.23	8.36	170	29.45	7.56	167	30.00	8.54	530	29.91	8.16
**DRUG TREATMENT**													
Treatment	Methadone	119	58.05		106	60.92		90	52.02		315	57.07	
	Buprenorphine (Sub)	86	41.95		68	39.08		83	47.98		237	42.93	

For peer review only

For peer review only - http://bmjopen.bmj.com/site/about/guidelines.xhtml

Treatment dose	Methadone	119	34.45	13.05	103	31.41	7.49	90	34.32	11.90	312	33.41	11.20
(mean mg)	Buprenorphine (Sub)	85	6.87	4.67	68	5.90	3.15	81	6.56	3.20	234	6.48	3.80
Median times in opiate treatment		198	2	1 to 4	172	2	1 to 3	171	2	1 to 5	541	2	1 to 4

For peer review only

Table 2 Urine Results - Heroin

(using opiates and 6MAM variables in combination + recoding missing urines to positive if participant DNA or refused to provide = positive assumption)

	Week 9				Week 10				Week 11				Week 12			
	CM Abstinence	CM Attendance	TAU	TOTAL	CM Abstinence	CM Attendanc	TAU	TOTAL	CM Abstinence	CM Attendanc	TAU	TOTAL	CM Abstinence	CM Attendanc	TAU	TOTAL
Neg	73	58	44	175	59	67	44	170	70	78	51	199	82	95	62	239
	35.61	33.33	25.43	31.70	28.78	38.51	25.43	30.80	34.15	44.83	29.48	36.05	40.00	54.60	35.84	43.30
Pos	77	78	86	241	96	71	85	252	80	67	83	230	69	56	73	198
	37.56	44.83	49.71	43.66	46.83	40.80	49.13	45.65	39.02	38.51	47.98	41.67	33.66	32.18	42.20	35.87
MISSING	55	38	43	136	50	36	44	130	55	29	39	123	54	23	38	115
	26.83	21.84	24.86	24.64	24.39	20.69	25.43	23.55	26.83	16.67	22.54	22.28	26.34	13.22	21.97	20.83
Total	205	174	173	552	205	174	173	552	205	174	173	552	205	174	173	552

For peer review only - http://bmjopen.bmj.com/site/about/guidelines.xhtml

	**Week 21**				**Week 22**				**Week 23**				**Week 24**			
	CM Abstinence	CM Attendanc	TAU	TOTAL	CM Abstinence	CM Attendanc	TAU	TOTAL	CM Abstinence	CM Attendanc	TAU	TOTAL	CM Abstinence	CM Attendanc	TAU	TOTAL
Neg	63	68	45	176	51	36	30	117	47	40	36	123	56	52	49	157
	30.73	39.08	26.01	31.88	24.88	20.69	17.34	21.20	22.93	22.99	20.81	22.28	27.32	29.89	28.32	28.44
Pos	75	56	73	204	87	80	87	254	86	81	78	245	75	72	66	213
	36.59	32.18	42.20	36.96	42.44	45.98	50.29	46.01	41.95	46.55	45.09	44.38	36.59	41.38	38.15	38.59
MISSING	67	50	55	172	67	58	56	181	72	53	59	184	74	50	58	182
	32.68	28.74	31.79	31.16	32.68	33.33	32.37	32.79	35.12	30.46	34.10	33.33	36.10	28.74	33.53	32.97
Total	205	174	173	552	205	174	173	552	205	174	173	552	205	174	173	552

For peer review only

For peer review only - http://bmjopen.bmj.com/site/about/guidelines.xhtml

Table 3: Primary outcome analysis: Odds of subjects providing a heroin-negative urine over weeks 9 to 12 and 21 to 24

	Contrast	Odds Ratio	95% CI		P-Value
**Weeks 9 to 12**					
(1) Outcome: repeated binary heroin result with refused & DNA=Positive sample (N= 483, no multiple imputation)	CM Abstinence vs TAU	1.67	0.89	3.14	.114
	CM Attendance vs TAU*	2.19	1.15	4.15	.017
	CM Attendance vs CM Abstinence	1.31	0.71	2.44	.391
(2) Outcome: repeated binary heroin result with refused & DNA=positive sample (i.e. Analysis 1) and remaining missing multiply imputed (N=552, using multiple imputation)	CM Abstinence vs TAU	1.59	0.85	3.01	.146
	CM Attendance vs TAU*	2.05	1.07	3.91	.030
	CM Attendance vs CM Abstinence	1.29	0.68	2.41	.438
**Weeks 21 to 24**					
(1) Repeated binary analysis for weeks 21, 22, 23, 24 with refused & DNA=0 (N= 409, no multiple imputation)	CM Abstinence vs TAU	1.71	0.72	4.09	.225
	CM Attendance vs TAU	1.66	0.69	4.00	.261
	CM Attendance vs CM Abstinence	0.97	0.41	2.28	.939
(2) Analysis (1) with remaining missing multiply imputed (N=552, using multiple imputation)	CM Abstinence vs TAU	1.46	0.66	3.26	.352
	CM Attendance vs TAU	1.29	0.57	2.92	.543
	CM Attendance vs CM Abstinence	0.89	0.40	1.93	.751

**significant at 0.05*

For peer review only - http://bmjopen.bmj.com/site/about/guidelines.xhtml

Table 4 **Opiate Treatment Index (OPI) measures of Heroin use in last 30 days measured at Baseline, 12 and 24 weeks.**

	CM Abstinence n=205	CM Attendance n = 174	TAU n= 173	Total n = 552
**Summary statistics for binary Yes/No to 'use in the past 30 days'?** (Missing shown but n% proportions calculated disregarding missing.)	N (%)	N (%)	N (%)	N (%)
**Baseline**				
Used heroin in last 30 days	205 (100)	173 (99)	172 (99)	550 (99.6)
Did not use heroin in last 30 days	0 (0)	1 (1)	1 (1)	2 (0.4)
Missing	*0*	*0*	*0*	*0*
**12 weeks**				
Used heroin in last 30 days	96 (73)	111 (78)	84 (72)	291 (74)
Did not use heroin in last 30 days	36 (27)	32 (22)	32 (28)	100 (26)
Missing	*73*	*31*	*57*	*161*
**24 weeks**				
Used heroin in last 30 days	97 (79)	85 (78)	66 (75)	248 (78)
Did not use heroin in last 30 days	26 (21)	24 (22)	22 (25)	72 (23)
Missing	*82*	*65*	*85*	*232*
**Number of days taking heroin in last 30 days**				
**Baseline**				
n	204	173	173	550
Number missing	1	1	0	2
Proportion missing	0.49	0.57	0	0.36
Median (25th, 75th percentile)	28.00 (22.00, 30.00)	27.00 (21.00, 30.00)	28.00 (21.00, 30.00)	28.00 (21.00, 30.00)
**12 weeks**				
n	132	143	116	391
Number missing	73	31	57	161
Proportion missing	35.61	17.82	32.95	29.17
Median (25th, 75th percentile)	4.00 (0.00, 15.00)	5.00 (1.00, 13.00)	4.00 (0.00, 15.00)	4.00 (0.00, 15.00)
**24 weeks**				
n	123	108	88	319
Number missing	82	66	85	233
Proportion missing	40.00	37.93	49.13	42.21
Median (25th, 75th percentile)	5.00 (1.00, 15.00)	4.50 (1.00, 15.00)	3.50 (0.50, 20.00)	4.00 (1.00, 15.00)

For peer review only

Table 5 **Opiate Treatment Index (OPI) measures of non-opioid drug use in last 30 days . Use in last 30 days (binary) and N of days used.** (Missing shown but n% proportions calculated disregarding missing

	**CM Abstinence n=205**	**CM Attendance n = 174**	**TAU n= 173**	**Total n = 552**
**CRACK**				
**Baseline**				
Used crack in last 30 days	161 (79)	128 (74)	130 (75)	419 (76)
Did not use crack in last 30 days	44 (22)	46 (26)	43 (25)	133 (24)
Missing	*0*	*0*	*0*	*0*
**12 weeks**				
Used crack in last 30 days	75 (57)	73 (52)	62 (53)	210 (54)
Did not use crack in last 30 days	57 (43)	68 (48)	54 (47)	179 (46)
Missing	*73*	*33*	*57*	*163*
**24 weeks**				
Used crack in last 30 days	78 (63)	56 (51)	52 (58)	186 (58)
Did not use crack in last 30 days	45 (37)	53 (49)	37 (42)	135 (42)
Missing	*82*	*65*	*84*	*231*
Number of days taking crack in last 30 days				
**Baseline**				
n	203	174	172	549
Number missing	2	0	1	3
Proportion missing	0.98	0	0.58	0.54
Median (25th, 75th percentile)	10.00 (1.00, 26.00)	7.00 (0.00, 20.00)	5.00 (0.50, 24.50)	7.00 (1.00, 24.00)
**12 weeks**				
n	132	140	116	388
Number missing	73	34	57	164
Proportion missing	35.61	19.54	32.95	29.71
Median (25th, 75th percentile)	1.00 (0.00, 10.00)	1.00 (0.00, 4.00)	1.00 (0.00, 4.00)	1.00 (0.00, 5.00)
**24 weeks**				
n	123	109	89	321
Number missing	82	65	84	231
Proportion missing	40	37.36	48.55	41.85
Median (25th, 75th percentile)	2.00 (0.00, 6.00)	1.00 (0.00, 4.00)	1.00 (0.00, 4.00)	1.00 (0.00, 5.00)
**BENZODIAZEPENES**				
**Baseline**				
Used benzos in last 30 days	47 (23)	35 (20)	47 (27)	129 (23)
Did not use benzos in last 30 days	158 (77)	138 (80)	126 (73)	422 (77)
Missing	*0*	*1*	*0*	*1*
**12 weeks**				

For peer review only - http://bmjopen.bmj.com/site/about/guidelines.xhtml

For peer review only

Used benzos in last 30 days	20 (15)	16 (11)	26 (22)	62 (16)
Did not use benzos in last 30 days	111 (85)	125 (89)	90 (78)	326 (84)
Missing	*74*	*33*	*57*	*164*
**24 weeks**				
Used benzos in last 30 days	20 (16)	9 (8)	16 (18)	45 (14)
Did not use benzos in last 30 days	103 (84)	100 (92)	73 (82)	276 (86)
Missing	*82*	*65*	*84*	*231*
Number of days taking benzodiazepines in last 30 days				
**Baseline**				
n	205	172	173	550
Number missing	0	2	0	2
Proportion missing	0	1.15	0	0.36
Median (25th, 75th percentile)	0.00 (0.00, 0.00)	0.00 (0.00, 0.00)	0.00 (0.00, 1.00)	0.00 (0.00, 0.00)
**12 weeks**				
n	131	139	116	386
Number missing	74	35	57	166
Proportion missing	36.10	20.11	32.95	30.07
Median (25th, 75th percentile)	0.00 (0.00, 0.00)	0.00 (0.00, 0.00)	0.00 (0.00, 0.00)	0.00 (0.00, 0.00)
**24 weeks**				
n	123	109	89	321
Number missing	82	65	84	231
Proportion missing	40.00	37.36	48.55	41.85
Median (25th, 75th percentile)	0.00 (0.00, 0.00)	0.00 (0.00, 0.00)	0.00 (0.00, 0.00)	0.00 (0.00, 0.00)
**ALCOHOL**				
**Baseline**				
Used alcohol in last 30 days	117 (57)	93 (53)	100 (58)	310 (56)
No alcohol use in last 30 days	88 (43)	81 (47)	73 (42)	242 (44)
Missing	*0*	*0*	*0*	*0*
**12 weeks**				
Used alcohol in last 30 days	77 (58)	83 (59)	76 (66)	236 (61)
No alcohol use in last 30 days	55 (42)	59 (42)	40 (35)	154 (40)
Missing	*73*	*32*	*57*	*162*
**24 weeks**				
Used alcohol in last 30 days	70 (57)	67 (62)	51 (57)	188 (59)
No alcohol use in last 30 days	53 (43)	42 (39)	38 (43)	133 (41)
Missing	*82*	*65*	*84*	*231*
Number of days taking alcohol in last 30 days				
**Baseline**				
n	204	172	172	548
Number missing	1	2	1	4
Proportion missing	0.49	1.15	0.58	0.72

For peer review only - http://bmjopen.bmj.com/site/about/guidelines.xhtml

Median (25th, 75th percentile)	1.00 (0.00, 10.00)	1.00 (0.00, 10.00)	1.00 (0.00, 8.00)	1.00 (0.00, 10.00)
**12 weeks**				
n	132	142	115	389
Number missing	73	32	58	163
Proportion missing	35.61	18.39	33.53	29.53
Median (25th, 75th percentile)	1.00 (0.00, 9.50)	2.00 (0.00, 10.00)	2.00 (0.00, 8.00)	2.00 (0.00, 10.00)
**24 weeks**				
n	123	107	89	319
Number missing	82	67	84	233
Proportion missing	40.00	38.51	48.55	42.21
Median (25th, 75th percentile)	1.00 (0.00, 9.00)	2.00 (0.00, 10.00)	2.00 (0.00, 12.00)	2.00 (0.00, 10.00)
**TOBACCO**				
**Baseline**				
Used tobacco in last 30 days	198 (97)	167 (96)	166 (96)	531 (96)
No tobacco use in last 30 days	7 (3)	7 (4)	7 (4)	21 (4)
Missing	*0*	*0*	*0*	*0*
**12 weeks**				
Used tobacco in last 30 days	129 (98)	134 (94)	107 (93)	370 (95)
No tobacco use in last 30 days	3 (2)	8 (6)	8 (7)	19 (5)
Missing	*73*	*32*	*58*	*163*
**24 weeks**				
Used tobacco in last 30 days	119 (97)	101 (93)	79 (89)	299 (93)
No tobacco use in last 30 days	4 (3)	8 (7)	10 (11)	22 (7)
Missing	*82*	*65*	*84*	*231*
**Number of days taking tobacco in last 30 days**				
**Baseline**				
n	198	171	171	540
Number missing	7	3	2	12
Proportion missing	3.41	1.72	1.16	2.17
Median (25th, 75th percentile)	30.00 (30.00, 30.00)	30.00 (30.00, 30.00)	30.00 (30.00, 30.00)	30.00 (30.00, 30.00)
**12 weeks**				
n	131	139	113	383
Number missing	74	35	60	169
Proportion missing	36.10	20.11	34.68	30.62
Median (25th, 75th percentile)	30.00 (30.00, 30.00)	30.00 (30.00, 30.00)	30.00 (30.00, 30.00)	30.00 (30.00, 30.00)
**24 weeks**				
n	123	109	87	319
Number missing	82	65	86	233

For peer review only - http://bmjopen.bmj.com/site/about/guidelines.xhtml

Proportion missing	40.00	37.36	49.71	42.21
Median (25th, 75th percentile)	30.00 (30.00, 30.00)	30.00 (30.00, 30.00)	30.00 (30.00, 30.00)	30.00 (30.00, 30.00)

For peer review only

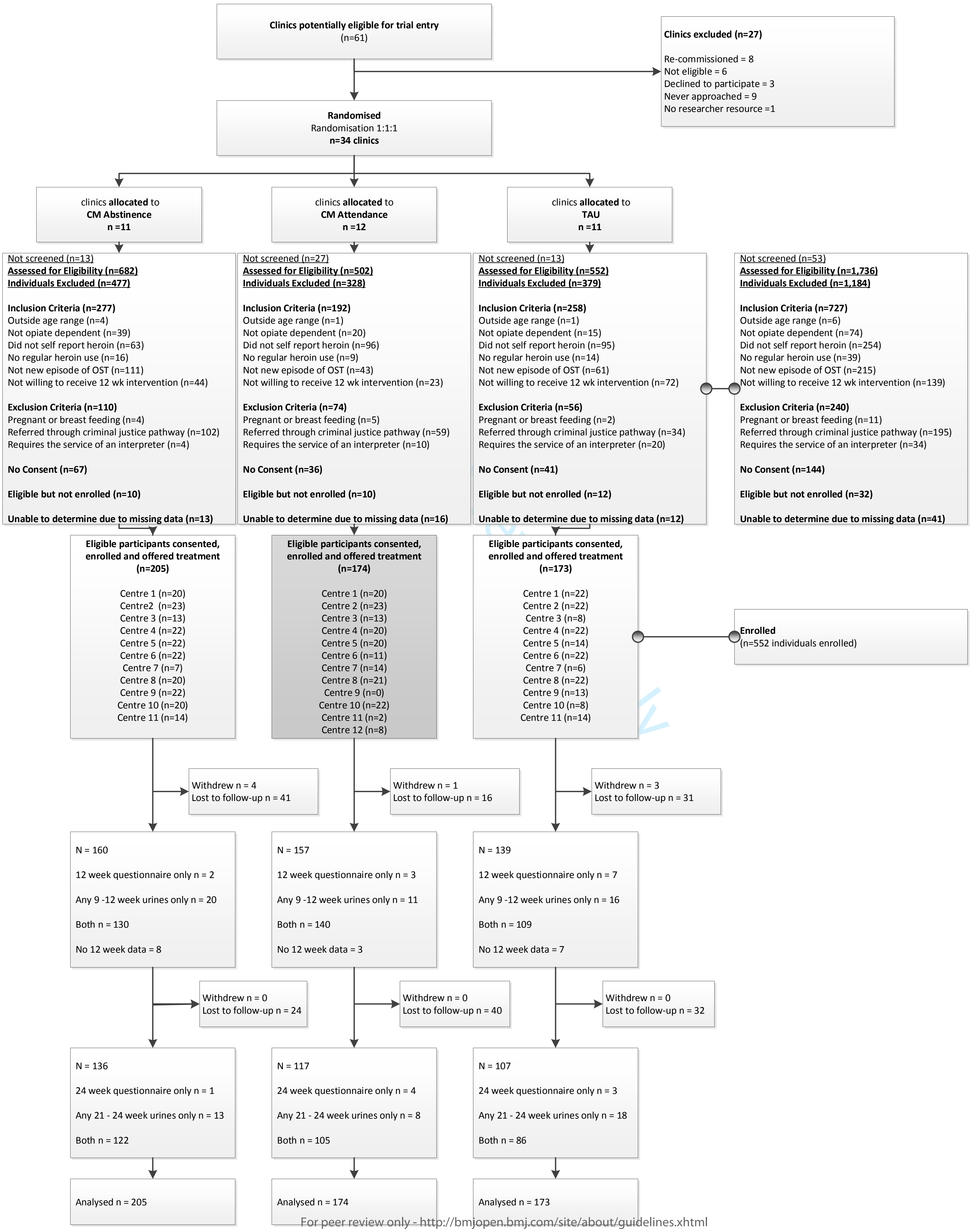
Clinics potentially eligible for trial entry
(n=61)
Clinics excluded (n=27)
Re-commissioned = 8
Not eligible = 6
Declined to participate = 3
Never approached = 9
No researcher resource =1
Randomised
Randomisation 1:1:1
n=34 clinics
clinics allocated to
CM Abstinence
n =11
clinics allocated to
CM Attendance
n =12
clinics allocated to
TAU
n =11
Not screened (n=13)
Assessed for Eligibility (n=682)
Individuals Excluded (n=477)
Inclusion Criteria (n=277)
Outside age range (n=4)
Not opiate dependent (n=39)
Did not self report heroin (n=63)
No regular heroin use (n=16)
Not new episode of OST (n=111)
Not willing to receive 12 wk intervention (n=44)
Exclusion Criteria (n=110)
Pregnant or breast feeding (n=4)
Referred through criminal justice pathway (n=102)
Requires the service of an interpreter (n=4)
No Consent (n=67)
Eligible but not enrolled (n=10)
Unable to determine due to missing data (n=13)
Not screened (n=27)
Assessed for Eligibility (n=502)
Individuals Excluded (n=328)
Inclusion Criteria (n=192)
Outside age range (n=1)
Not opiate dependent (n=20)
Did not self report heroin (n=96)
No regular heroin use (n=9)
Not new episode of OST (n=43)
Not willing to receive 12 wk intervention (n=23)
Exclusion Criteria (n=74)
Pregnant or breast feeding (n=5)
Referred through criminal justice pathway (n=59)
Requires the service of an interpreter (n=10)
No Consent (n=36)
Eligible but not enrolled (n=10)
Unable to determine due to missing data (n=16)
Not screened (n=13)
Assessed for Eligibility (n=552)
Individuals Excluded (n=379)
Inclusion Criteria (n=258)
Outside age range (n=1)
Not opiate dependent (n=15)
Did not self report heroin (n=95)
No regular heroin use (n=14)
Not new episode of OST (n=61)
Not willing to receive 12 wk intervention (n=72)
Exclusion Criteria (n=56)
Pregnant or breast feeding (n=2)
Referred through criminal justice pathway (n=34)
Requires the service of an interpreter (n=20)
No Consent (n=41)
Eligible but not enrolled (n=12)
Unable to determine due to missing data (n=12)
Not screened (n=53)
Assessed for Eligibility (n=1,736)
Individuals Excluded (n=1,184)
Inclusion Criteria (n=727)
Outside age range (n=6)
Not opiate dependent (n=74)
Did not self report heroin (n=254)
No regular heroin use (n=39)
Not new episode of OST (n=215)
Not willing to receive 12 wk intervention (n=139)
Exclusion Criteria (n=240)
Pregnant or breast feeding (n=11)
Referred through criminal justice pathway (n=195)
Requires the service of an interpreter (n=34)
No Consent (n=144)
Eligible but not enrolled (n=32)
Unable to determine due to missing data (n=41)
Eligible participants consented, enrolled and offered treatment
(n=205)
Centre 1 (n=20)
Centre2 (n=23)
Centre 3 (n=13)
Centre 4 (n=22)
Centre 5 (n=22)
Centre 6 (n=22)
Centre 7 (n=7)
Centre 8 (n=20)
Centre 9 (n=22)
Centre 10 (n=20)
Centre 11 (n=14)
Eligible participants consented, enrolled and offered treatment
(n=174)
Centre 1 (n=20)
Centre 2 (n=23)
Centre 3 (n=13)
Centre 4 (n=20)
Centre 5 (n=20)
Centre 6 (n=11)
Centre 7 (n=14)
Centre 8 (n=21)
Centre 9 (n=0)
Centre 10 (n=22)
Centre 11 (n=2)
Centre 12 (n=8)
Eligible participants consented, enrolled and offered treatment
(n=173)
Centre 1 (n=22)
Centre 2 (n=22)
Centre 3 (n=8)
Centre 4 (n=22)
Centre 5 (n=14)
Centre 6 (n=22)
Centre 7 (n=6)
Centre 8 (n=22)
Centre 9 (n=13)
Centre 10 (n=8)
Centre 11 (n=14)
Enrolled
(n=552 individuals enrolled)
Withdrew n = 4
Lost to follow-up n = 41
Withdrew n = 1
Lost to follow-up n = 16
Withdrew n = 3
Lost to follow-up n = 31
N = 160
12 week questionnaire only n = 2
Any 9 -12 week urines only n = 20
Both n = 130
No 12 week data = 8
N = 157
12 week questionnaire only n = 3
Any 9 -12 week urines only n = 11
Both n = 140
No 12 week data = 3
N = 139
12 week questionnaire only n = 7
Any 9 -12 week urines only n = 16
Both n = 109
No 12 week data = 7
Withdrew n = 0
Lost to follow-up n = 24
Withdrew n = 0
Lost to follow-up n = 40
Withdrew n = 0
Lost to follow-up n = 32
N = 136
24 week questionnaire only n = 1
Any 21 - 24 week urines only n = 13
Both n = 122
N = 117
24 week questionnaire only n = 4
Any 21 - 24 week urines only n = 8
Both n = 105
N = 107
24 week questionnaire only n = 3
Any 21 - 24 week urines only n = 18
Both n = 86
Analysed n = 205
Analysed n = 174
Analysed n = 173

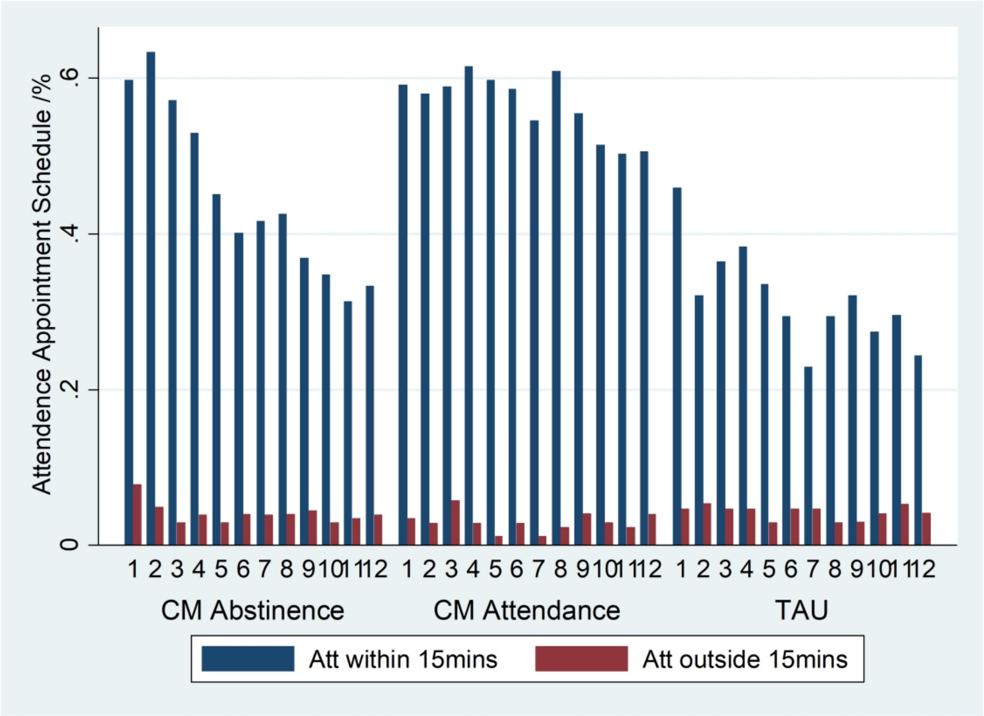

Figure 2. Attendance at weekly appointments by treatment group

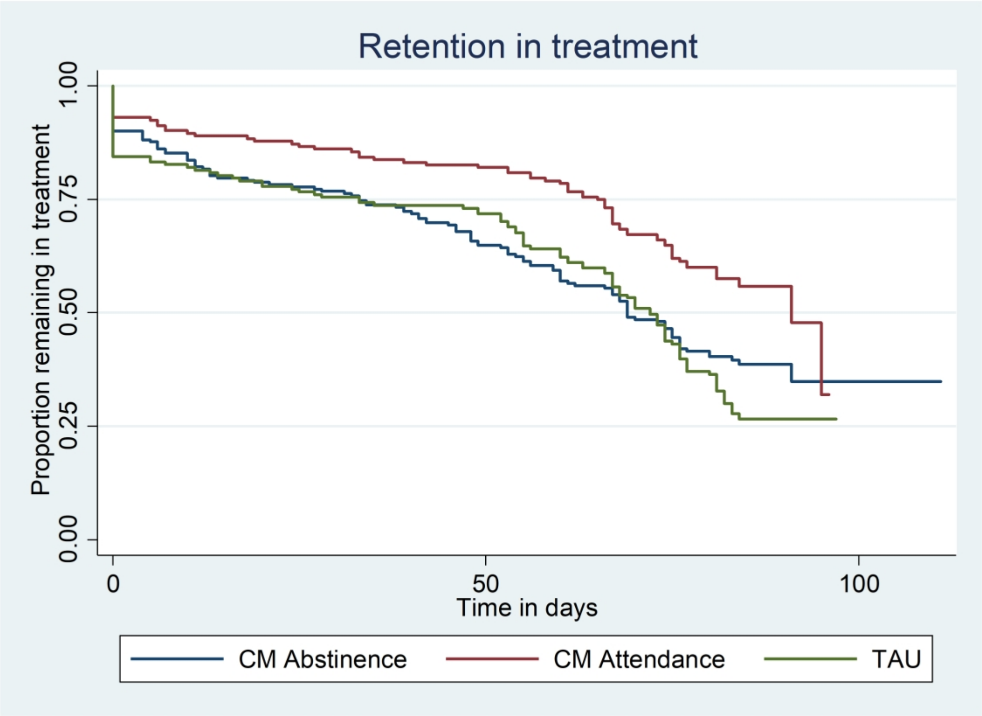

Figure 3. Time to continuous treatment non-attendance (Kaplan Meier plot) by treatment group.

593x433mm (144 x 144 DPI)

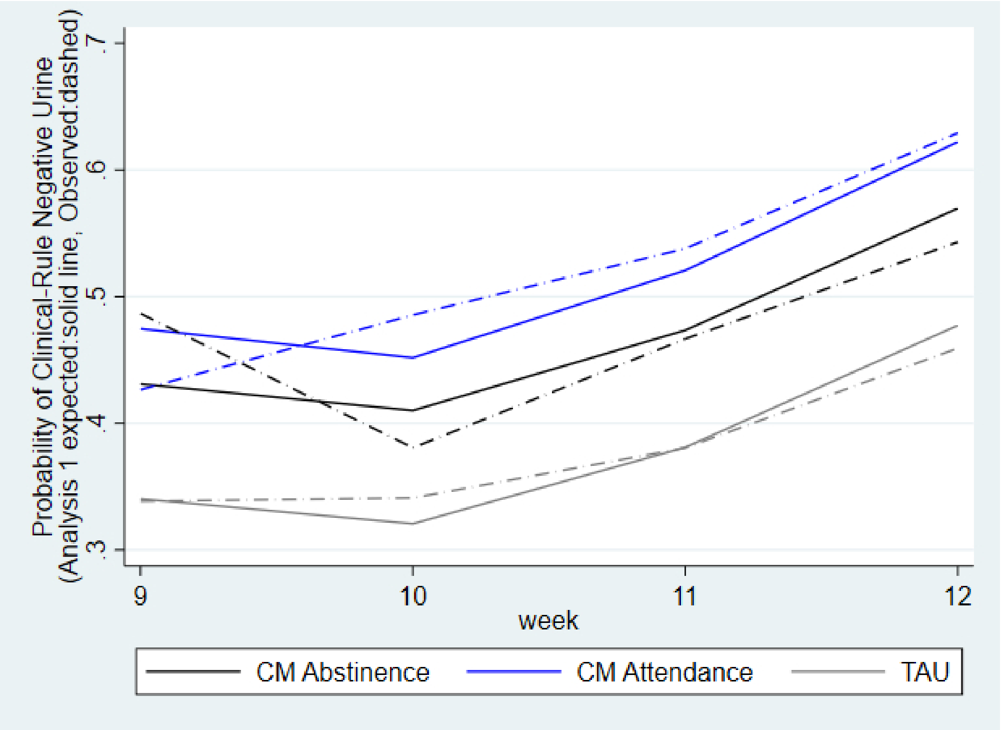

Figure 4a. Probability of negative urine applying positive assumption over weeks 9-12 and 21-24

218x158mm (72 x 72 DPI)

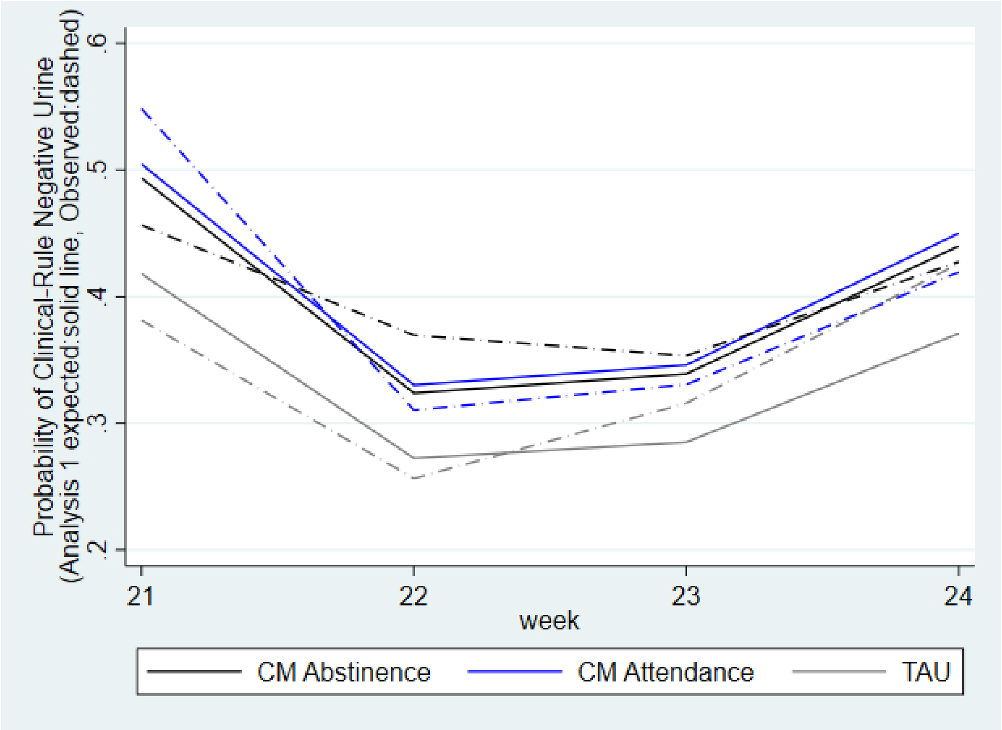

Figure 4b. Probability of negative urine applying positive assumption over weeks 9-12 and 21-24

218x158mm (72 x 72 DPI)

Metrebian, Weaver & Goldsmith et al. Using a pragmatically adapted, low-cost, contingency management intervention to promote heroin abstinence in individuals undergoing treatment for opioid substance use disorder in UK drug services (PRAISE): a cluster randomised trial

Supplementary material

For peer review only

Table 1. Clinic characteristics by Arm

		CM Abstinence			CM Attendance			TAU			TOTAL		
		N	Mean / or median %	SD or IQR	N	Mean / or median %	SD or IQR	N	Mean / or median %	SD or IQR	N	Mean / or median %	SD or IQR
**Clinic type**<br>**N (%) NHS**		6	55		7	58		8	73		21	62	
**Clinic location**<br>**N (%) London**		7	63		3	25		6	55		16	47	
**Birmingham**		2	18		2	17		1	9		5	15	
**Dudley & Walsall**		0	0		1	8		0	0		1	3	
**Herts**		1	9		4	33		1	9		6	18	
**South Essex**		1	9		1	8		0	0		2	6	
**Sussex**		0	0		1	8		2	18		3	9	
**Avon & Wiltshire**		0	0		0	0		1	9		1	3	
**Clinic total**		11	100		12	100		11	100		34	100	

For peer review only

For peer review only

Table 2 Opiate Treatment Index (OPI) measures of non-opioid drug use in last 30 days . Use in last 30 days (binary) and N of days used. (Missing shown but n% proportions calculated disregarding missing

	CM Abstinence n=205	CM Attendance n = 174	TAU n= 173	Total n = 552
**CRACK**				
**Baseline**				
Used crack in last 30 days	161 (79)	128 (74)	130 (75)	419 (76)
Did not use crack in last 30 days	44 (22)	46 (26)	43 (25)	133 (24)
Missing	*0*	*0*	*0*	*0*
**12 weeks**				
Used crack in last 30 days	75 (57)	73 (52)	62 (53)	210 (54)
Did not use crack in last 30 days	57 (43)	68 (48)	54 (47)	179 (46)
Missing	*73*	*33*	*57*	*163*
**24 weeks**				
Used crack in last 30 days	78 (63)	56 (51)	52 (58)	186 (58)
Did not use crack in last 30 days	45 (37)	53 (49)	37 (42)	135 (42)
Missing	*82*	*65*	*84*	*231*
Number of days taking crack in last 30 days				
**Baseline**				
n	203	174	172	549
Number missing	2	0	1	3
Proportion missing	0.98	0	0.58	0.54
Median (25th, 75th percentile)	10.00 (1.00, 26.00)	7.00 (0.00, 20.00)	5.00 (0.50, 24.50)	7.00 (1.00, 24.00)
**12 weeks**				
n	132	140	116	388
Number missing	73	34	57	164
Proportion missing	35.61	19.54	32.95	29.71

For peer review only - http://bmjopen.bmj.com/site/about/guidelines.xhtml

Median (25th, 75th percentile)	1.00 (0.00, 10.00)	1.00 (0.00, 4.00)	1.00 (0.00, 4.00)	1.00 (0.00, 5.00)
**24 weeks**				
n	123	109	89	321
Number missing	82	65	84	231
Proportion missing	40	37.36	48.55	41.85
Median (25th, 75th percentile)	2.00 (0.00, 6.00)	1.00 (0.00, 4.00)	1.00 (0.00, 4.00)	1.00 (0.00, 5.00)
**BENZODIAZEPENES**				
**Baseline**				
Used benzos in last 30 days	47 (23)	35 (20)	47 (27)	129 (23)
Did not use benzos in last 30 days	158 (77)	138 (80)	126 (73)	422 (77)
Missing	*0*	*1*	*0*	*1*
**12 weeks**				
Used benzos in last 30 days	20 (15)	16 (11)	26 (22)	62 (16)
Did not use benzos in last 30 days	111 (85)	125 (89)	90 (78)	326 (84)
Missing	*74*	*33*	*57*	*164*
**24 weeks**				
Used benzos in last 30 days	20 (16)	9 (8)	16 (18)	45 (14)
Did not use benzos in last 30 days	103 (84)	100 (92)	73 (82)	276 (86)
Missing	*82*	*65*	*84*	*231*
Number of days taking benzodiazepines in last 30 days				
**Baseline**				
n	205	172	173	550
Number missing	0	2	0	2
Proportion missing	0	1.15	0	0.36
Median (25th, 75th percentile)	0.00 (0.00, 0.00)	0.00 (0.00, 0.00)	0.00 (0.00, 1.00)	0.00 (0.00, 0.00)
**12 weeks**				
n	131	139	116	386
Number missing	74	35	57	166
Proportion missing	36.10	20.11	32.95	30.07

For peer review only

Median (25th, 75th percentile)	0.00 (0.00, 0.00)	0.00 (0.00, 0.00)	0.00 (0.00, 0.00)	0.00 (0.00, 0.00)
**24 weeks**				
n	123	109	89	321
Number missing	82	65	84	231
Proportion missing	40.00	37.36	48.55	41.85
Median (25th, 75th percentile)	0.00 (0.00, 0.00)	0.00 (0.00, 0.00)	0.00 (0.00, 0.00)	0.00 (0.00, 0.00)
**ALCOHOL**				
**Baseline**				
Used alcohol in last 30 days	117 (57)	93 (53)	100 (58)	310 (56)
No alcohol use in last 30 days	88 (43)	81 (47)	73 (42)	242 (44)
Missing	*0*	*0*	*0*	*0*
**12 weeks**				
Used alcohol in last 30 days	77 (58)	83 (59)	76 (66)	236 (61)
No alcohol use in last 30 days	55 (42)	59 (42)	40 (35)	154 (40)
Missing	*73*	*32*	*57*	*162*
**24 weeks**				
Used alcohol in last 30 days	70 (57)	67 (62)	51 (57)	188 (59)
No alcohol use in last 30 days	53 (43)	42 (39)	38 (43)	133 (41)
Missing	*82*	*65*	*84*	*231*
Number of days taking alcohol in last 30 days				
**Baseline**				
n	204	172	172	548
Number missing	1	2	1	4
Proportion missing	0.49	1.15	0.58	0.72
Median (25th, 75th percentile)	1.00 (0.00, 10.00)	1.00 (0.00, 10.00)	1.00 (0.00, 8.00)	1.00 (0.00, 10.00)
**12 weeks**				
n	132	142	115	389
Number missing	73	32	58	163
Proportion missing	35.61	18.39	33.53	29.53

For peer review only

Median (25th, 75th percentile)	1.00 (0.00, 9.50)	2.00 (0.00, 10.00)	2.00 (0.00, 8.00)	2.00 (0.00, 10.00)
**24 weeks**				
n	123	107	89	319
Number missing	82	67	84	233
Proportion missing	40.00	38.51	48.55	42.21
Median (25th, 75th percentile)	1.00 (0.00, 9.00)	2.00 (0.00, 10.00)	2.00 (0.00, 12.00)	2.00 (0.00, 10.00)
**TOBACCO**				
**Baseline**				
Used tobacco in last 30 days	198 (97)	167 (96)	166 (96)	531 (96)
No tobacco use in last 30 days	7 (3)	7 (4)	7 (4)	21 (4)
Missing	*0*	*0*	*0*	*0*
**12 weeks**				
Used tobacco in last 30 days	129 (98)	134 (94)	107 (93)	370 (95)
No tobacco use in last 30 days	3 (2)	8 (6)	8 (7)	19 (5)
Missing	*73*	*32*	*58*	*163*
**24 weeks**				
Used tobacco in last 30 days	119 (97)	101 (93)	79 (89)	299 (93)
No tobacco use in last 30 days	4 (3)	8 (7)	10 (11)	22 (7)
Missing	*82*	*65*	*84*	*231*
**Number of days taking tobacco in last 30 days**				
**Baseline**				
n	198	171	171	540
Number missing	7	3	2	12
Proportion missing	3.41	1.72	1.16	2.17
Median (25th, 75th percentile)	30.00 (30.00, 30.00)	30.00 (30.00, 30.00)	30.00 (30.00, 30.00)	30.00 (30.00, 30.00)
**12 weeks**				
n	131	139	113	383
Number missing	74	35	60	169
Proportion missing	36.10	20.11	34.68	30.62

For peer review only

Median (25th, 75th percentile)	30.00 (30.00, 30.00)	30.00 (30.00, 30.00)	30.00 (30.00, 30.00)	30.00 (30.00, 30.00)
**24 weeks**				
n	123	109	87	319
Number missing	82	65	86	233
Proportion missing	40.00	37.36	49.71	42.21
Median (25th, 75th percentile)	30.00 (30.00, 30.00)	30.00 (30.00, 30.00)	30.00 (30.00, 30.00)	30.00 (30.00, 30.00)

Table 3 **Odds ratios for Opiate Treatment Index (OPI) measures of drug use in the last 30 days assessed at 12 and 24 weeks: Use in last 30 days (binary) and N of days used.**

	Treatment group difference estimate	Lower confidence limit	Upper confidence limit	p-value
**HEROIN**<br>**Use in last 30 days Y/N across groups – GEE model results**				
12 weeks CM Attendance v CM Abstinence	1.28	0.77	2.14	0.336
12 weeks TAU v CM Abstinence	0.98	0.56	1.70	0.936
12 weeks TAU v CM Attendance	0.76	0.47	1.23	0.268
24 weeks CM Attendance v CM Abstinence	0.95	0.51	1.78	0.876
24 weeks TAU v CM Abstinence	0.80	0.40	1.63	0.545
24 weeks TAU v CM Attendance	0.85	0.42	1.69	0.633
**Mean differences N of days used in last 30 days across groups**				
12 weeks CM Attendance v CM Abstinence	-0.42	-3.11	2.27	0.760
12 weeks TAU v CM Abstinence	0.02	-2.77	2.80	0.990
12 weeks TAU v CM Attendance	0.44	-2.33	3.21	0.757
24 weeks CM Attendance v CM Abstinence	-0.25	-3.06	2.56	0.859
24 weeks TAU v CM Abstinence	-0.13	-3.06	2.80	0.931
24 weeks TAU v CM Attendance	0.13	-2.85	3.10	0.934
**CRACK**				

For peer review only

**Use in last 30 days Y/N across groups – GEE model results**				
12 weeks CM Attendance v CM Abstinence	0.83	0.37	1.85	0.654
12 weeks TAU v CM Abstinence	0.90	0.46	1.78	0.768
12 weeks TAU v CM Attendance	1.08	0.59	1.99	0.795
24 weeks CM Attendance v CM Abstinence	0.71	0.37	1.37	0.307
24 weeks TAU v CM Abstinence	0.75	0.35	1.64	0.477
24 weeks TAU v CM Attendance	1.06	0.50	2.27	0.878
**Mean differences N of days used in last 30 days across groups**				
12 weeks CM Attendance v CM Abstinence	-1.80	-3.90	0.30	0.093
12 weeks TAU v CM Abstinence	-1.67	-3.84	0.50	0.132
12 weeks TAU v CM Attendance	0.13	-2.02	2.29	0.903
24 weeks CM Attendance v CM Abstinence	0.06	-2.13	2.25	0.956
24 weeks TAU v CM Abstinence	-0.30	-2.58	1.99	0.800
24 weeks TAU v CM Attendance	-0.36	-2.67	1.96	0.762
**BENZODIAZEPENES**<br>**Use in last 30 days Y/N across groups – GEE model results**				
12 weeks CM Attendance v CM Abstinence	0.65	0.20	2.13	0.480
12 weeks TAU v CM Abstinence	1.31	0.51	3.41	0.573
12 weeks TAU v CM Attendance	2.01	0.57	7.09	0.276
24 weeks CM Attendance v CM Abstinence	0.46	0.15	1.39	0.167
24 weeks TAU v CM Abstinence	0.88	0.33	2.32	0.794
24 weeks TAU v CM Attendance	1.92	0.70	5.28	0.204
**Mean differences N of days used in last 30 days across groups**				
12 weeks CM Attendance v CM Abstinence	-0.16	-0.94	0.62	0.688
12 weeks TAU v CM Abstinence	0.32	-0.49	1.14	0.436
12 weeks TAU v CM Attendance	0.48	-0.32	1.29	0.239
24 weeks CM Attendance v CM Abstinence	0.23	-0.61	1.06	0.591
24 weeks TAU v CM Abstinence	0.01	-0.87	0.90	0.976
24 weeks TAU v CM Attendance	-0.22	-1.12	0.69	0.642

For peer review only

**ALCOHOL**<br>**Use in last 30 days Y/N across groups – GEE model results**				
12 weeks CM Attendance v CM Abstinence	1.34	0.81	2.21	0.255
12 weeks TAU v CM Abstinence	1.55	0.78	3.07	0.208
12 weeks TAU v CM Attendance	1.16	0.59	2.29	0.674
24 weeks CM Attendance v CM Abstinence	1.86	1.01	3.45	0.048
24 weeks TAU v CM Abstinence	1.08	0.66	1.77	0.762
24 weeks TAU v CM Attendance	0.58	0.29	1.17	0.126
**Mean differences N of days used in last 30 days across groups**				
12 weeks CM Attendance v CM Abstinence	1.07	-0.59	2.72	0.206
12 weeks TAU v CM Abstinence	1.00	-0.74	2.75	0.259
12 weeks TAU v CM Attendance	-0.06	-1.78	1.65	0.943
24 weeks CM Attendance v CM Abstinence	0.20	-1.56	1.96	0.825
24 weeks TAU v CM Abstinence	0.87	-0.98	2.72	0.357
24 weeks TAU v CM Attendance	0.67	-1.21	2.56	0.485
**TOBACCO**<br>**Mean differences N of days used in last 30 days across groups**				
12 weeks CM Attendance v CM Abstinence	-0.50	-2.10	1.09	0.536
12 weeks TAU v CM Abstinence	-0.25	-1.93	1.42	0.769
12 weeks TAU v CM Attendance	0.25	-1.40	1.90	0.765
24 weeks CM Attendance v CM Abstinence	-0.95	-2.64	0.75	0.273
24 weeks TAU v CM Abstinence	-2.07	-3.87	-0.27	0.024
24 weeks TAU v CM Attendance	-1.13	-2.96	0.71	0.228

For peer review only

Table 4. Secondary Outcome Measures Summary Statistics: Alcohol Use Disorders (AUDIT), Social Functioning (OTI), physical and mental wellbeing (SF-36), depression and anxiety (HADS), crime (AD-SUS).

	CM Abstinence n=205	CM Attendance n = 174	TAU n= 173	Total n = 552
**AUDIT (Alcohol Use)**				
**Baseline**				
n	205	174	173	552
Number missing	0	0	0	0
Proportion missing	0	0	0	0
Mean (SD)	9.69 (10.42)	9.37 (9.71)	9.18 (9.88)	9.43 (10.02)
**12 weeks**				
n	131	142	116	389
Number missing	74	32	57	163
Proportion missing	36.10	18.39	32.95	29.53
Mean (SD)	8.79 (9.47)	8.39 (9.72)	8.13 (8.22)	8.45 (9.19)
**24 weeks**				
n	123	108	89	320
Number missing	82	66	84	232
Proportion missing	40.00	37.93	48.55	42.03
Mean (SD)	9.67 (9.46)	7.94 (9.01)	8.67 (8.62)	8.81 (9.08)
**SOCIAL FUNCTIONING**				
**Baseline**				
n	205	174	173	552
Number missing	0	0	0	0
Proportion missing	0	0	0	0
Mean (SD)	19.59 (6.36)	17.80 (7.41)	18.29 (6.41)	18.62 (6.75)
**12 weeks**				

For peer review only

n	132	143	115	390
Number missing	73	31	58	162
Proportion missing	35.61	17.82	33.53	29.35
Mean (SD)	17.45 (7.36)	16.31 (6.25)	16.37 (6.39)	16.71 (6.69)
**24 weeks**				
n	123	109	89	321
Number missing	82	65	84	231
Proportion missing	40	37.36	48.55	41.85
Mean (SD)	16.46 (7.07)	15.63 (6.69)	15.91 (5.96)	16.02 (6.64)
**MENTAL WELLBEING (SF36)**				
**Baseline**				
n	204	173	173	550
Number missing	1	1	0	2
Proportion missing	0.49	0.57	0	0.36
Mean (SD)	30.29 (14.02)	31.88 (13.98)	30.15 (15.14)	30.75 (14.36)
**12 weeks**				
n	132	140	115	387
Number missing	73	33	58	164
Proportion missing	35.61	18.97	33.53	29.71
Mean (SD)	37.44 (14.95)	37.56 (14.02)	34.12 (15.65)	36.50 (14.88)
**24 weeks**				
n	121	109	88	318
Number missing	84	65	85	234
Proportion missing	40.98	37.36	49.13	42.39
Mean (SD)	35.97 (13.68)	36.38 (14.22)	38.43 (15.19)	36.79 (14.29)
**PHYSICAL WELLBEING (SF36)**				
**Baseline**				
n	204	173	173	550
Number missing	1	1	0	2

For peer review only

Proportion missing	0.49	0.57	0	0.36
Mean (SD)	47.06 (8.84)	46.69 (10.62)	48.02 (11.15)	47.25 (10.17)
**12 weeks**				
n	132	141	115	388
Number missing	73	33	58	164
Proportion missing	35.61	18.97	33.53	29.71
Mean (SD)	48.37 (10.02)	47.72 (11.08)	47.06 (11.14)	47.74 (10.73)
**24 weeks**				
n	121	109	88	318
Number missing	84	65	85	234
Proportion missing	40.98	37.36	49.13	42.39
Mean (SD)	49.20 (10.11)	48.09 (11.59)	48.72 (10.96)	48.69 (10.85)
**DEPRESSION (HADS)**				
**Baseline**				
n	205	173	173	551
Number missing	0	1	0	1
Proportion missing	0	0.57	0	0.18
Mean (SD)	9.51 (4.42)	9.42 (5.00)	9.46 (4.84)	9.47 (4.73)
**12 weeks**				
n	132	142	116	390
Number missing	73	32	57	162
Proportion missing	35.61	18.39	32.95	29.35
Mean (SD)	7.58 (5.07)	7.59 (4.68)	8.28 (4.75)	7.79 (4.83)
**24 weeks**				
n	123	109	89	321
Number missing	82	65	84	231
Proportion missing	40	37.36	48.55	41.85
Mean (SD)	7.72 (4.44)	7.46 (4.72)	7.02 (5.46)	7.44 (4.83)
**ANXIETY (HADS)**				

For peer review only

**Baseline**				
n	205	173	173	551
Number missing	0	1	0	1
Proportion missing	0	0.57	0	0.18
Mean (SD)	10.58 (5.12)	9.75 (5.20)	10.31 (5.44)	10.23 (5.25)
**12 weeks**				
n	132	142	116	390
Number missing	73	32	57	162
Proportion missing	35.61	18.39	32.95	29.35
Mean (SD)	8.79 (5.21)	8.77 (5.18)	9.59 (5.55)	9.02 (5.30)
**24 weeks**				
n	123	109	89	321
Number missing	82	65	84	231
Proportion missing	40	37.36	48.55	41.85
Mean (SD)	9.10 (5.13)	8.71 (5.11)	7.83 (5.78)	8.61 (5.32)
**CRIME**				
**Baseline**				
Yes	70 (34)	47 (27)	67 (39)	184 (33)
No	135 (66)	125 (72)	106 (61)	366 (66)
Missing	0	2 (1)	0	2 (1)
**12 weeks (n=133)**				
Yes	29 (14)	23 (13)	28 (16)	80 (14)
no	104 (51)	119 (69)	91 (53)	314 (57)
Missing	72 (35)	32 (18)	55 (32)	160 (29)
**24 weeks (n=125)**				
Yes	41(20)	28 (16)	25 (14)	94 (17)
No	84 (41)	81 (47)	65 (38)	230 (42)
Missing	80 (39)	65 (37)	83 (48)	228 (41)

For peer review only

**Number of crimes**				
**Baseline**				
n	129	97	204	430
Number missing	0	0	0	0
Proportion missing	0	0	0	0
Mean (SD)	5.16 (8.19)	3.88 (9.92)	8.16 (15.89)	5.73 (11.94)
**Week 12**				
n	71	44	118	233
Number missing	72	32	55	159
Proportion missing	35	18	32	29
Mean (SD)	2.84 (6.03)	1.76 (5.09)	4.72 (10.75)	3.11 (7.79)
**Week 24**				
n	86	120	122	323
Number missing	65	80	83	228
Proportion missing	37	39	48	41
Mean (SD)	3.44 (6.87)	4.8 (14.86)	4.88 (10.52)	4.37 (11.25

Most crimes committed: Baseline : theft from a shop (173) , handling stolen goods (60), and assault (20); week 12: theft from a shop (110), handling stolen goods (34), and begging (14); and week 24: theft from a shop (165), handling stolen goods (35) and drug trafficking (18).

For peer review only

Table 5. **Odds ratios for Secondary Outcome Measures: Social Functioning (OPI), Alcohol Use Disorders (AUDIT) physical and mental wellbeing (SF-36), quality of life (EQ-5D) depression and anxiety (HADS).**

	Treatment group difference estimate	Lower confidence limit	Upper confidence limit	p-value
**Mean differences in AUDIT across groups**				
12 weeks CM Attendance v CM Abstinence	0.42	-1.17	2.02	0.605
12 weeks TAU v CM Abstinence	0.45	-1.22	2.11	0.600
12 weeks TAU v CM Attendance	0.02	-1.62	1.67	0.977
24 weeks CM Attendance v CM Abstinence	-0.99	-2.68	0.69	0.247
24 weeks TAU v CM Abstinence	-0.17	-1.93	1.60	0.854
24 weeks TAU v CM Attendance	0.83	-0.97	2.63	0.366
**Mean differences in OPIATE TREATMENT INDEX Social Functioning across groups**				
12 weeks CM Attendance v CM Abstinence	0.24	-1.06	1.54	0.715
12 weeks TAU v CM Abstinence	-0.08	-1.44	1.28	0.912
12 weeks TAU v CM Attendance	-0.32	-1.65	1.02	0.640
24 weeks CM Attendance v CM Abstinence	0.32	-1.07	1.70	0.655
24 weeks TAU v CM Abstinence	0.10	-1.36	1.56	0.889
24 weeks TAU v CM Attendance	-0.21	-1.70	1.28	0.781
**Mean differences in SF36 MCS across groups**				
12 weeks CM Attendance v CM Abstinence	-1.08	-4.45	2.29	0.529
12 weeks TAU v CM Abstinence	-2.49	-5.98	1.00	0.162
12 weeks TAU v CM Attendance	-1.41	-4.89	2.07	0.427
24 weeks CM Attendance v CM Abstinence	-1.76	-5.29	1.77	0.330
24 weeks TAU v CM Abstinence	1.19	-2.49	4.87	0.526
24 weeks TAU v CM Attendance	2.95	-0.80	6.69	0.123
**Mean differences in SF36 PCS across groups**				
12 weeks CM Attendance v CM Abstinence	-0.35	-2.33	1.62	0.727

For peer review only

12 weeks TAU v CM Abstinence	-2.06	-4.13	0.02	0.052
12 weeks TAU v CM Attendance	-1.70	-3.75	0.35	0.103
24 weeks CM Attendance v CM Abstinence	-0.90	-3.01	1.21	0.402
24 weeks TAU v CM Abstinence	-0.75	-2.98	1.48	0.511
24 weeks TAU v CM Attendance	0.15	-2.11	2.42	0.895
**Mean differences in HADS depression across groups**				
12 weeks CM Attendance v CM Abstinence	0.08	-1.05	1.22	0.885
12 weeks TAU v CM Abstinence	0.69	-0.48	1.87	0.249
12 weeks TAU v CM Attendance	0.61	-0.56	1.78	0.309
24 weeks CM Attendance v CM Abstinence	-0.17	-1.36	1.03	0.784
24 weeks TAU v CM Abstinence	-0.62	-1.86	0.63	0.331
24 weeks TAU v CM Attendance	-0.45	-1.72	0.82	0.487
**Mean differences in HADS anxiety across groups**				
12 weeks CM Attendance v CM Abstinence	0.46	-0.84	1.76	0.485
12 weeks TAU v CM Abstinence	0.33	-1.00	1.67	0.624
12 weeks TAU v CM Attendance	-0.13	-1.46	1.21	0.851
24 weeks CM Attendance v CM Abstinence	0.48	-0.87	1.83	0.482
24 weeks TAU v CM Abstinence	-0.81	-2.21	0.58	0.252
24 weeks TAU v CM Attendance	-1.30	-2.72	0.12	0.073

For peer review only

Table 6. ARM-5 – Therapeutic Alliance

	CM Abstinence n=205	CM Attendance n = 174	TAU n= 173	Total n = 552
**ARM-5 mean score**				
**Week 4**				
n	91	84	36	211
Number missing	114	90	137	341
Proportion missing	55.61	51.72	79.19	61.78
Mean (SD)	6.54 (0.73)	6.71 (0.63)	6.26 (1.09)	6.56 (0.78)
**Week 8**				
n	63	69	28	160
Number missing	142	105	145	392
Proportion missing	69.27	60.34	83.82	71.01
Mean (SD)	6.60 (0.78)	6.59 (0.69)	6.16 (1.01)	6.52 (0.80)
**Week 12**				
n	123	126	91	340
Number missing	82	48	82	212
Proportion missing	40.00	27.59	47.40	38.41
Mean (SD)	6.43 (0.96)	6.27 (1.27)	5.88 (1.30)	6.23 (1.19)

For peer review only

For peer review only

Table 1: CONSORT 2010 checklist of information to include when reporting a cluster randomised trial

Section/Topic	Item No	Standard Checklist item	Extension for cluster designs	Page No *
**Title and abstract**				
	1a	Identification as a randomised trial in the title	Identification as a cluster randomised trial in the title	1
	1b	Structured summary of trial design, methods, results, and conclusions (for specific guidance see CONSORT for abstracts)[1,2]	See table 2	2
**Introduction**				
**Background and objectives**	2a	Scientific background and explanation of rationale	Rationale for using a cluster design	4
	2b	Specific objectives or hypotheses	Whether objectives pertain to the the cluster level, the individual participant level or both	4
**Methods**				
**Trial design**	3a	Description of trial design (such as parallel, factorial) including allocation ratio	Definition of cluster and description of how the design features apply to the clusters	3/4/5
	3b	Important changes to methods after trial commencement (such as eligibility criteria), with reasons		**7**
**Participants**	4a	Eligibility criteria for participants	Eligibility criteria for clusters	4
	4b	Settings and locations where the data were collected		**3**
**Interventions**	5	The interventions for each group with sufficient details to allow replication, including how and when they were actually administered	Whether interventions pertain to the cluster level, the individual participant level or both	5

**Outcomes**	6a	Completely defined pre-specified primary and secondary outcome measures, including how and when they were assessed	Whether outcome measures pertain to the cluster level, the individual participant level or both	6
	6b	Any changes to trial outcomes after the trial commenced, with reasons		**N/A**
**Sample size**	7a	How sample size was determined	Method of calculation, number of clusters(s) (and whether equal or unequal cluster sizes are assumed), cluster size, a coefficient of intracluster correlation (ICC or *k*), and an indication of its uncertainty	6/7
	7b	When applicable, explanation of any interim analyses and stopping guidelines		
**Randomisation:**				
**Sequence generation**	8a	Method used to generate the random allocation sequence		**4/5**
	8b	Type of randomisation; details of any restriction (such as blocking and block size)	Details of stratification or matching if used	4/5
**Allocation concealment mechanism**	9	Mechanism used to implement the random allocation sequence (such as sequentially numbered containers), describing any steps taken to conceal the sequence until interventions were assigned	Specification that allocation was based on clusters rather than individuals and whether allocation concealment (if any) was at the cluster level, the individual participant level or both	5
**Implementation**	10	Who generated the random allocation sequence, who enrolled participants, and who assigned participants to interventions	Replace by 10a, 10b and 10c	4/5
	10a		Who generated the random allocation sequence, who enrolled clusters, and who	4/5

			assigned clusters to interventions	
	10b		Mechanism by which individual participants were included in clusters for the purposes of the trial (such as complete enumeration, random sampling)	4/5
	10c		From whom consent was sought (representatives of the cluster, or individual cluster members, or both), and whether consent was sought before or after randomisation	6
**Blinding**	11a	If done, who was blinded after assignment to interventions (for example, participants, care providers, those assessing outcomes) and how		**5**
	11b	If relevant, description of the similarity of interventions		**5**
**Statistical methods**	12a	Statistical methods used to compare groups for primary and secondary outcomes	How clustering was taken into account	7
	12b	Methods for additional analyses, such as subgroup analyses and adjusted analyses		**7**
**Results**				
**Participant flow (a diagram is strongly recommended)**	13a	For each group, the numbers of participants who were randomly assigned, received intended treatment, and were analysed for the primary outcome	For each group, the numbers of clusters that were randomly assigned, received intended treatment, and were analysed for the primary outcome	Figure 1 (page 8)

	13b	For each group, losses and exclusions after randomisation, together with reasons	For each group, losses and exclusions for both clusters and individual cluster members	Figure 1 and supplementary materials table 1
**Recruitment**	14a	Dates defining the periods of recruitment and follow-up		**Page 8**
	14b	Why the trial ended or was stopped		**N/A**
**Baseline data**	15	A table showing baseline demographic and clinical characteristics for each group	Baseline characteristics for the individual and cluster levels as applicable for each group	Table 1, 4 & 5 and supplementary materials Tables 2-4
**Numbers analysed**	16	For each group, number of participants (denominator) included in each analysis and whether the analysis was by original assigned groups	For each group, number of clusters included in each analysis	Figure 1, Table 2,4,5.
**Outcomes and estimation**	17a	For each primary and secondary outcome, results for each group, and the estimated effect size and its precision (such as 95% confidence interval)	Results at the individual or cluster level as applicable and a coefficient of intracluster correlation (ICC or k) for each primary outcome	Pages 10/11 . tables 3.Supplementary Table 3 & 5
	17b	For binary outcomes, presentation of both absolute and relative effect sizes is recommended		**Pages 10/11**
**Ancillary analyses**	18	Results of any other analyses performed, including subgroup analyses and adjusted analyses, distinguishing pre-specified from exploratory		**Pages 10/11**
**Harms**	19	All important harms or unintended effects in each group (for specific guidance see CONSORT for harms[3])		**Page 11**
**Discussion**				
**Limitations**	20	Trial limitations, addressing sources of potential bias,		**Page 12 & 13**

		imprecision, and, if relevant, multiplicity of analyses		
**Generalisability**	21	Generalisability (external validity, applicability) of the trial findings	Generalisability to clusters and/or individual participants (as relevant)	Page 13
**Interpretation**	22	Interpretation consistent with results, balancing benefits and harms, and considering other relevant evidence		**Page 12 & 13**
**Other information**				
**Registration**	23	Registration number and name of trial registry		**Page 16**
**Protocol**	24	Where the full trial protocol can be accessed, if available		**Attached document. Published Protocol.**
**Funding**	25	Sources of funding and other support (such as supply of drugs), role of funders		**Page 16**

** Note: page numbers optional depending on journal requirements*

For peer review only

Table 2: Extension of CONSORT for abstracts[1,2] to reports of cluster randomised trials

Item	Standard Checklist item	Extension for cluster trials
**Title**	Identification of study as randomised	**Identification of study as cluster randomised**
**Trial design**	Description of the trial design (e.g. parallel, cluster, non-inferiority)	
**Methods**		
**Participants**	Eligibility criteria for participants and the settings where the data were collected	**Eligibility criteria for clusters**
**Interventions**	Interventions intended for each group	
**Objective**	Specific objective or hypothesis	**Whether objective or hypothesis pertains to the cluster level, the individual participant level or both**
**Outcome**	Clearly defined primary outcome for this report	**Whether the primary outcome pertains to the cluster level, the individual participant level or both**
**Randomization**	How participants were allocated to interventions	**How clusters were allocated to interventions**
**Blinding (masking)**	Whether or not participants, care givers, and those assessing the outcomes were blinded to group assignment	
**Results**		
**Numbers randomized**	Number of participants randomized to each group	**Number of clusters randomized to each group**
**Recruitment**	Trial status[1]	
**Numbers analysed**	Number of participants analysed in each group	**Number of clusters analysed in each group**
**Outcome**	For the primary outcome, a result for each group and the estimated effect size and its precision	**Results at the cluster or individual participant level as applicable for each primary outcome**
**Harms**	Important adverse events or side effects	
**Conclusions**	General interpretation of the results	
**Trial registration**	Registration number and name of trial register	
**Funding**	Source of funding	

[1] Relevant to Conference Abstracts

REFERENCES

[1] Hopewell S, Clarke M, Moher D, Wager E, Middleton P, Altman DG, et al. CONSORT for reporting randomised trials in journal and conference abstracts. *Lancet* 2008, 371:281-283

[2] Hopewell S, Clarke M, Moher D, Wager E, Middleton P, Altman DG at al (2008) CONSORT for reporting randomized controlled trials in journal and conference abstracts: explanation and elaboration. *PLoS Med* 5(1): e20

[3] Ioannidis JP, Evans SJ, Gotzsche PC, O'Neill RT, Altman DG, Schulz K, Moher D. Better reporting of harms in randomized trials: an extension of the CONSORT statement. *Ann Intern Med* 2004; 141(10):781-788.

For peer review only

BMJ Open

Using a pragmatically adapted, low-cost, contingency management intervention to promote heroin abstinence in individuals undergoing treatment for heroin use disorder in UK drug services (PRAISE): a cluster randomised trial

Journal:	*BMJ Open*
Manuscript ID	bmjopen-2020-046371.R1
Article Type:	Original research
Date Submitted by the Author:	01-Mar-2021
Complete List of Authors:	Metrebian, Nicola; King's College London Institute of Psychiatry Psychology and Neuroscience, Addiction<br>Weaver, Tim ; Middlesex University<br>Goldsmith, Kimberley; King's College London, Biostatistics; Institute of Psychiatry, King's College London<br>Pilling, Stephen; University College London, Centre for Outcomes, Research & Effectiveness<br>Hellier, Jennifer; King's College London Institute of Psychiatry Psychology and Neuroscience, Biostatistics and Health Informatics<br>Pickles, Andrew; King's College London Institute of Psychiatry Psychology and Neuroscience, Biostatistics and Health Informatics<br>Shearer, James; King's College London Institute of Psychiatry Psychology and Neuroscience, Health Services and Population Research<br>Byford, Sarah; King's College London Institute of Psychiatry Psychology and Neuroscience, Health Service & Population Research Department.<br>Mitcheson, Luke; South London and Maudsley NHS Foundation Trust<br>Bijral, Prun; Change, Grow, Live, Management Offices<br>Bogdan, Nadine; Essex Partnership University NHS Foundation Trust<br>Bowden-Jones, Owen; Central and North West London NHS Foundation Trust<br>Day, Edward; Birmingham and Solihull Mental Health NHS Foundation Trust<br>Dunn, John; Camden and Islington NHS Foundation Trust<br>Glasper, Anthony; Sussex Partnership NHS Foundation Trust, Hastings Substance Misuse Service<br>Finch, Emily; South London and Maudsley NHS Foundation Trust<br>Forshall, Sam; Avon and Wiltshire Mental Health Partnership NHS Trust<br>Akhtar, Shabana; Birmingham and Solihull Mental Health NHS Foundation Trust<br>Bajaria, Jalpa; Essex Partnership University NHS Foundation Trust, R&D; National Institute for Health Research, CRN North West London<br>Bennett, Carmel; Birmingham and Solihull Mental Health NHS Foundation Trust<br>Bishop, Elizabeth; University College London<br>Charles, Vikki; King's College London Institute of Psychiatry Psychology and Neuroscience, Addiction<br>Davey, Clare; Avon and Wiltshire Mental Health Partnership NHS Trust<br>Desai, Roopal; King's College London Institute of Psychiatry Psychology

	and Neuroscience, Addiction<br>Goodfellow, Claire; University of Glasgow MRC/CSO Social and Public Health Sciences Unit, ; University of Glasgow, MRC / CSO Social and Public Health Sciences Unit<br>Haque, Farjana; King's College London Institute of Psychiatry Psychology and Neuroscience, Addiction<br>Little, Nicholas; University College London, Outcomes, Effectiveness<br>McKechnie, Hortencia; King's College London Institute of Psychiatry Psychology and Neuroscience, Addiction; Imperial College London Faculty of Medicine, Mental Health<br>Mosler, Franziska ; King's College London Institute of Psychiatry Psychology and Neuroscience, Addictions<br>Morris, Jo; Avon and Wiltshire Mental Health Partnership NHS Trust, Research & Development<br>Mutz, J; Imperial College London, Epidemiology and Biostatistics<br>Pauli, Ruth; University of Birmingham<br>Poovendran, Dilkushi; Imperial College London Faculty of Medicine<br>Phillips, Elizabeth; Essex Partnership University NHS Foundation Trust<br>Strang, John; King's College London Institute of Psychiatry Psychology and Neuroscience, Addictions
**Primary Subject Heading**:	Addiction
Secondary Subject Heading:	Addiction, Health services research, Evidence based practice
Keywords:	Substance misuse < PSYCHIATRY, Clinical trials < THERAPEUTICS, Adult psychiatry < PSYCHIATRY

SCHOLARONE™
Manuscripts

I, the Submitting Author has the right to grant and does grant on behalf of all authors of the Work (as defined in the below author licence), an exclusive licence and/or a non-exclusive licence for contributions from authors who are: i) UK Crown employees; ii) where BMJ has agreed a CC-BY licence shall apply, and/or iii) in accordance with the terms applicable for US Federal Government officers or employees acting as part of their official duties; on a worldwide, perpetual, irrevocable, royalty-free basis to BMJ Publishing Group Ltd ("BMJ") its licensees and where the relevant Journal is co-owned by BMJ to the co-owners of the Journal, to publish the Work in this journal and any other BMJ products and to exploit all rights, as set out in our licence.

The Submitting Author accepts and understands that any supply made under these terms is made by BMJ to the Submitting Author unless you are acting as an employee on behalf of your employer or a postgraduate student of an affiliated institution which is paying any applicable article publishing charge ("APC") for Open Access articles. Where the Submitting Author wishes to make the Work available on an Open Access basis (and intends to pay the relevant APC), the terms of reuse of such Open Access shall be governed by a Creative Commons licence – details of these licences and which Creative Commons licence will apply to this Work are set out in our licence referred to above.

Other than as permitted in any relevant BMJ Author's Self Archiving Policies, I confirm this Work has not been accepted for publication elsewhere, is not being considered for publication elsewhere and does not duplicate material already published. I confirm all authors consent to publication of this Work and authorise the granting of this licence.

For peer review only

Using a pragmatically adapted, low-cost, contingency management intervention to promote heroin abstinence in individuals undergoing treatment for heroin use disorder in UK drug services (PRAISE): a cluster randomised trial

Nicola Metrebian [a*], Tim Weaver [b,c*], Kimberley Goldsmith [d], Prof Stephen Pilling [e], Jennifer Hellier [d], Prof Andrew Pickles [d], James Shearer [f], Prof Sarah Byford [f], Luke Mitcheson [g] Prun H. Bijral [h], Nadine Bogdan [i], Owen Bowden-Jones [j], Edward Day [k], John Dunn [l], Anthony Glasper [n], Emily Finch[g], Sam Forshall[m], Shabana Akhtar[k], Jalpa Bajaria[j], Carmel Bennett[k], Elizabeth Bishop[e], Vikki Charles[a], Clare Davey[m], Roopal Desai[a], Claire Goodfellow[e], Farjana Haque[a], Nicholas Little[e], Hortencia McKechnie[b,a], Franziska Mosler[a], Jo Morris[m], Julian Mutz[a], Ruth Pauli[k], Dilkushi Poovendran [b], Elizabeth Phillips [l] and Prof Sir John Strang [a,g]

* Joint first authors.

on behalf of the Contingency Management Programme Team

a King's College London, National Addiction Centre, Institute of Psychiatry, Psychology & Neuroscience, London, UK
b Imperial College London, London, UK
c Middlesex University, London, UK

dKing's College London, Department of Biostatistics and Health Informatics, Institute of Psychiatry, Psychology & Neuroscience, London, UK
e University College London, London, UK
f King's College London, Health Service and Population Research Department, Institute of Psychiatry, Psychology & Neuroscience, London, UK
g South London and Maudsley NHS Foundation Trust, London, UK
h Change, Grow, Live Charity, Management Offices, London, UK
i South Essex Partnership NHS Foundation Trust, Essex, UK
j Central and North West London NHS Foundation Trust, London, UK
k Birmingham & Solihull Mental Health NHS Foundation Trust, Birmingham, UK
l Camden & Islington NHS Foundation Trust, London, UK
m Avon & Wiltshire Mental Health Partnership NHS Trust, Bristol, UK
n Sussex Partnership NHS Foundation Trust, Brighton, UK

Corresponding author: Dr Nicola Metrebian, Addictions Department, Institute of Psychiatry, Psychology and Neuroscience, King's College London, 4 Windsor Walk, Denmark Hill, London SE5 8AA. Nicola.metrebian@kcl.ac.uk

Trial registration ISRCTN 01591254

For peer review only

For peer review only - http://bmjopen.bmj.com/site/about/guidelines.xhtml

Abstract

Introduction: Most individuals treated for heroin use disorder receive opioid agonist treatment (OAT)(methadone or buprenorphine). However, OAT is associated with high attrition and persistent, occasional heroin use. There is some evidence for the effectiveness of contingency management (CM), a behavioural intervention involving modest financial incentives, in encouraging drug-abstinence when applied adjunctively with OAT. UK drug services have a minimal track record of applying CM and limited resources to implement it. We assessed a CM intervention pragmatically adapted for ease of implementation in UK drug services to promote heroin-abstinence among individuals receiving OAT.

Design: Cluster randomised controlled trial

Setting and participants: 552 adults with heroin use disorder (target 660) enrolled from 34 clusters (drug treatment clinics) in England between November 2012 and October 2015.

Interventions: Clusters were randomly allocated 1:1:1 to OAT plus 12x weekly appointments with: i)CM targeted at opiate-abstinence at appointments (CM Abstinence); ii)CM targeted at on-time attendance at appointments (CM Attendance); or, iii)no CM (Treatment As Usual;TAU). Modifications included monitoring behaviour weekly and fixed-incentives schedule.

Measurements: Primary outcome: heroin-abstinence measured by heroin-free urines (weeks 9-12). Secondary outcomes: heroin-abstinence 12 weeks after discontinuation of CM (weeks 21-24); attendance; self-reported drug use, physical and mental health.

Results: CM Attendance was superior to TAU in encouraging heroin-abstinence. Odds of a heroin-negative urine in weeks 9-12 was statistically significantly greater in CM Attendance compared to TAU (OR=2.1;95%CI:1.1 to 3.9,p=0.030). CM Abstinence was not superior to TAU (OR=1.6;95%CI:0.9 to 3.0,p=0.146) or CM Attendance (OR=1.3;95%CI:0.7 to 2.4,p=0.438) (not statistically significant differences). Reductions in heroin use were not sustained at 21-24 weeks. No differences between groups in self-reported heroin use.

Conclusions: A pragmatically adapted CM intervention for routine use in UK drug services was moderately effective in encouraging heroin-abstinence compared with no CM only when targeted at attendance. CM targeted at abstinence was not effective.

The trial is prospectively registered, number ISRCTN 01591254.

Funding: NIHR (RP-PG-0707-10149)

Strengths and Limitations

- To our knowledge, this is the first clinical trial to examine the effectiveness of contingency management in promoting heroin-abstinence among those in opioid agonist treatment in the UK.

For peer review only - http://bmjopen.bmj.com/site/about/guidelines.xhtml

- This study is a large, cluster randomised trial conducted at 34 drug treatment services in England
- Contingency management, as developed in the US, was pragmatically adapted for ease of implementation in resource poor UK substance use treatment settings
- Adaptions included using drug service staff to deliver all aspects of the CM intervention (as opposed to CM specialists); staff were trained, supported by a CM Handbook and regular supervision
- Illicit drug use was monitored through urine drug screens (UDS) once a week rather than more frequently using a fixed rather than escalating schedule,

Introduction

Opioid agonist treatment (OAT) (methadone and buprenorphine) is recognised globally as a clinically[1,2] and cost-effective[2,3] treatment for opiate use disorder. However, its effectiveness is often undermined by high attrition associated with relapse into illicit drug use.[4] In the United Kingdom (UK), the National Institute for Health and Care Excellence (NICE) recommends psychological therapies (including contingency management (CM)) be offered alongside OAT[2] to support behaviour change.

CM is a behavioural intervention based on the principles of operant conditioning[5], delivered as a time-limited adjunct to existing evidence-based treatments (such as OAT) to amplify patient benefit. It involves providing positive reinforcement (usually monetary vouchers or prizes) contingent on achieving pre-specified behaviour consistent with treatment goals.[6,7]

Evidence, primarily from US trials, shows that CM is an effective adjunct to substance use treatment in encouraging abstinence from drug use, including in treating drug use regardless of treatment setting, and for treating drug use (including cocaine, opiates and cocaine and poly-substance use) in opiate addiction treatment.[8,9,10,11,12] However, there is weaker evidence for the effectiveness of CM in encouraging abstinence from illicit opiates among those receiving OAT.[9,12, 13,14]

UK guidance based on the evidence at the time,[8,9] recommended CM should be utilised in UK drug treatment to target the reduction of illicit drug use and encourage attendance at appointments.[2,15] However, UK drug treatment services had little experience of applying CM, reduced treatment budgets and limited staff capacity; conditions likely to impede implementation of CM.[6] We aimed to assess a CM intervention pragmatically adapted for ease of implementation in routine UK practice. We have previously demonstrated that low-cost CM can affect short-term behaviour change of improved adherence to a hepatitis B vaccination schedule[16] achieving clear long-term health economic benefit.[17] The trial reported here focused on more complex and longer-term behaviour change. Specifically, we assessed the effectiveness of two different 12-week CM schedules targeting (i) opiate-abstinence or (ii) attendance at clinical appointments, providing immediate positive reinforcement through vouchers delivered by trained staff, in achieving heroin-abstinence among individuals receiving OAT in community drug-treatment settings.

For peer review only - http://bmjopen.bmj.com/site/about/guidelines.xhtml

Methods

Study design and setting

We employed a pragmatic cluster randomised controlled trial design described elsewhere.[18] The unit of randomization was the drug clinic (cluster) rather than individual participant. This was for three reasons; 1) to reduce the likelihood of contamination if staff were delivering and patients receiving different interventions at the same drug clinic; 2) to reduce inter-patient contamination as patients themselves constitute a local social network; and 3) to reduce the risk of low recruitment, poor compliance and high drop-out within TAU arm if participants receiving TAU were denied an incentive offered to others in the same clinic. Sites were recruited in stages and then randomized. Thirty-four drug treatment clinics (clusters) in England (NHS: London, Birmingham, Sussex, Essex, Bath & Bristol, Dudley & Walsall; Non-NHS London, Hertfordshire, and Birmingham) were randomly allocated to one of three conditions (described below) and tasked with recruiting a cluster-sample of 22 participants. Clinics were eligible if they provided OAT (methadone or buprenorphine), weekly clinical appointments and received enough OAT referrals to meet recruitment targets.

Within each cluster, participants received the same allocated condition thus minimising the risk of contamination between intervention and control arms.

The trial tested the following research hypotheses:

- CM (positive reinforcement targeted at treatment attendance) will increase abstinence from street-heroin when compared to treatment as usual (TAU) in which no positive reinforcement is offered.
- CM (positive reinforcement targeted at the provision of opiate-negative urine samples at treatment appointments) will increase abstinence from street-heroin when compared to TAU in which no positive reinforcement is offered.
- Differences in the type of CM schedules will be associated with differences in heroin-abstinence.

Having both CM interventions allowed us to assess whether any benefit (abstinence) derives from a direct effect of CM on abstinence as the behavioural target, or is a general benefit resulting from CM-stimulated improved attendance at clinical appointments (and possibly consequent improvements in treatment retention).

Findings from parallel economic and process evaluations are to be reported separately.

Participants

Eligible patients were aged 18 and above; seeking a new episode of OAT; regular users of street heroin (ie. self-reported use 15/preceding 30 days [at least 3 days a week], and all [minimum 1] urine drug screens (UDS) in previous month positive for opiates); meeting ICD-10 criteria for opiate dependence; willing to receive 12-week CM intervention; at liberty to participate in the study for 24 weeks; and willing and able to provide informed consent. We excluded patients if they: could not read English and required an interpreter to understand a brief oral description of the study to ensure

For peer review only - http://bmjopen.bmj.com/site/about/guidelines.xhtml

they would be able to understand the contingency management intervention provided; were pregnant or breastfeeding (due to being seen as a special population receiving special treatment provision); and/or were referred through the criminal justice pathway and were receiving a community sentence on condition of attending drug treatment as they would be subject to additional contingencies which might influence their behaviour.

Randomisation and masking

Each cluster (drug clinic) was randomly assigned to OAT plus 12 x weekly clinical appointments with either:

i) CM Abstinence: positive reinforcement contingent upon opiate-abstinence monitored through UDS undertaken at each weekly appointment.

ii) CM Attendance: positive reinforcement contingent upon attendance on time; or,

iii) no CM (TAU; control condition).

Randomisation was undertaken independently by the King's Clinical Trials Unit. Clusters were assigned to treatments using random permuted blocks within type of service provider strata (NHS or non-NHS) using a block length of 3 in a 1:1:1 allocation ratio. Laboratory personnel who completed the urinalysis and the statistician analysing primary outcome data were all blinded to treatment allocation.

Interventions

OAT was delivered in line with existing service protocols at all clinics and included weekly clinical appointments of between 15 and 50 minutes with a named drug worker. Attending the service to obtain a prescription did not constitute attendance. The CM interventions (described below) were delivered adjunctively.

In our trial, CM, as developed in the US, was pragmatically adapted for use in resource poor UK drug treatment services to ease future implementation. Key CM principles were retained; targeting a clearly defined behaviour, regular monitoring of that behaviour and providing an immediate reinforcer ensured that a clear contingent relationship was made between the positive behaviour and the reinforcer. The reinforcer was withheld if the target behaviour was not achieved. Adaptions included: (a) training drug workers to deliver CM (as opposed to CM specialists); b) monitoring illicit drug use through weekly UDS (rather than more frequently) to fit routine UK practice of taking UDS once a week at the start of treatment and less frequently thereafter; and c) using a fixed rather than escalating schedule, as drug service staff had previously reported the use of escalating schedules to be too difficult.[19] CM consisted of positive reinforcement - verbal praise and fixed value £10 supermarket voucher at each appointment. Reasoning that anything lower would be unlikely to encourage participants to attend while escalation of the value would increase the costs of the intervention beyond most services budget.

'*CM Abstinence*': Reinforcement was conditional on attendance at weekly appointments and (a) during weeks 1-4 (priming weeks) providing a UDS whatever the result and (b) during weeks 5-12 providing an opiate-negative UDS. UDS were undertaken using a drug integrated cup test to detect opioids. This test is able to detect opioids up to 1-3 days after use. Whilst, unable to confirm opioid abstinence over the week period, the test nevertheless provided clinically significant evidence of the

For peer review only - http://bmjopen.bmj.com/site/about/guidelines.xhtml

participants ability to abstain from using opioids over this briefer period. Instant UDS tests were completed at the start of each appointment. Staff provided the reinforcer immediately the target behaviour was achieved. Priming ensured participants gained experience of the reinforcer, while also addressing practitioner concerns about the difficulty in achieving abstinence during contemporaneous titration up to a stable dose.

'*CM Attendance'*: Eligibility to receive the reinforcement was conditional solely on on-time attendance at the appointment (within 15 minutes of the scheduled appointment time).
Control: Participants received treatment as usual (TAU) (OAT plus 12 x weekly appointments) with no CM.

The CM interventions ceased after week 12, at which point the frequency of appointments reverted to usual care for each service (ie. Varied depending on drug service and patient needs).

Training & supervision

All staff providing clinical appointments received training on trial procedures. At CM sites, staff received bespoke one-day training on the principles and practice of CM (including simulation, role play). Training was supported by a CM Handbook designed for UK services (Pilling S. et al, Developing an Evidence base for Contingency Management in UK Addiction Services, 2012) but which drew on international evidence and practice models.[20]

CM was delivered in the first 5-10 minutes of the appointment and audio recorded. A CM adherence measure was developed from measures available at the time[21] but adapted to assess key CM competencies in UK services settings. Audio recordings were used to rate the scale which comprised ten 4-point Likert scale items. Adherence was scored as poor (<33%), adequate (33-66%), or good (>66%). The scale achieved good interrater reliability (k=0.63;p<0.001).

Evidence suggests training alone is unlikely to change health professional behaviour unless supported by effective supervision.[22] Group supervision sessions were provided to staff at intervention sites throughout the trial. The drug service psychologist/senior practitioner provided local supervision after receiving training in CM supervision. Audio recordings of CM delivery were available to local supervisors to provide feedback to staff. In turn, research team psychologists used recordings of supervision sessions to support their supervision of local supervisors.

Research Assessments

Consenting participants completed a research interview (and were reimbursed £20 for time and travel) before enrolment into the trial and again at 12-and 24-weeks post enrolment. All participants were asked to provide (research) UDS weekly between weeks 9-12 and 21-24. Weekly "urine collection clinics" were established by the research team at each clinic (participants were reimbursed for travel) to enable participants to provide UDS outside of appointments. Participants' attendance at appointments, receipt of CM vouchers and provision of urines for CM and research purposes were recorded.

Outcomes

For peer review only - http://bmjopen.bmj.com/site/about/guidelines.xhtml

The primary outcome measure of heroin-abstinence was a once-weekly binary positive/negative heroin-UDS collected at weeks 9-12 for laboratory drug testing.

Secondary outcome measures included retention in treatment, self-reported illicit-drug use in the last 30 days,[23] alcohol use (Alcohol Use Disorders (AUDIT)),[24] social functioning (Opiate Treatment Index (OTI)),[25] physical and mental health status (Short Form-36 (SF-36)),[26] and depression and anxiety (Hospital Anxiety and Depression Scale (HADS)),[27] obtained by researchers in face to face interviews with patients at baseline, 12 and 24 weeks. Confidential patient ratings of therapeutic alliance with their health-professional were recorded at 4, 8 and 12-weeks post-enrolment (ARM5).[28] A single item measure of delay-discounting[29] and a motivation measure for drug abstinence[30] were ancillary outcome measures and reported elsewhere.

The Adult Service Use Schedule (AD-SUS) adapted for drug users,[31] health-related quality of life (EQ-5D-3L),[32] and the WHO Health and Work Performance Questionnaire[33] were measured for health economic analysis. Findings from the health economic analysis and parallel process evaluation are to be reported separately.

Sample size calculation

More detail is provided in the protocol.[18] Lussier et al [8] estimated a mean weighted effect size of 0.39 from three studies with an opiate-use outcome measure (CM v control). For a two-sided test, alpha = 0.05, 5% attrition, 80% power to detect an effect size of 0.39, 111 participants per group were required. Accounting for clustering by clinic, this was inflated to 220 participants per arm (assuming an intra-class correlation of 0.05, 20 participants per cluster and 11 clusters per intervention leading to a design effect of 1.95.) Clinics were initially advised to recruit >20 participants each (33 x 20 = 660 in total). After recruiting 13 clusters, with an attrition rate of 10%, larger than the expected 5%, clusters still recruiting were asked to increase recruitment from 20 to 22 participants.

Statistical analysis

Statistical analysis was carried out using Stata v14/15 according to the intention-to-treat principle. Variables were summarised using mean/standard deviation, median/quartiles, or frequencies/proportions as appropriate.

Refusal and non-attendance UDS were coded as positive (positive assumption) but results missing for other reasons (e.g. discharged from treatment, in hospital or in prison) were initially left missing. This positive assumption is commonly used in clinical practice and in studies of CM and was used in our previous trial.[34]

These responses were the dependent variables in a mixed effects logistic regression model (9-12 weeks and 21-24 weeks in separate models), with trial arm, the clinic type stratification factor and week dummy variables as fixed-effects, and random intercepts for participants nested within clinic. Results are presented both from fitting a model fitted to data where 1) the positive assumption has been applied, but no further imputation has taken place, 2) further missing data were imputed (100 datasets) and estimates obtained using Rubin's rules. The imputation model included clinic, trial arm, NHS stratification factor, gender, heroin use at 12 and 24 weeks, and baseline measures

For peer review only - http://bmjopen.bmj.com/site/about/guidelines.xhtml

including frequency of heroin use, AUDIT score, OTI social functioning score anxiety and depression, SF36 physical and mental component scores, and delay discounting, in addition to the four binary weekly urine sample measures. Moderation by type of opioid replacement (methadone or buprenorphine), NHS clinic versus non-NHS and the Charlson index of multiple drug use was tested using the non-imputed data by adding treatment by moderator interaction terms each separately to the model described.

Differences in secondary continuous outcomes between the groups were estimated using linear mixed effects models with the 12 and 24 week outcomes as dependent variables, the same fixed and random effects as for the primary outcome, plus time point, treatment by time interaction, and baseline values of the outcome where applicable as predictors. For the self-report binary drug use variables, marginal logistic generalised estimating equation (GEE) regression models were fitted (to estimate marginal instead of potentially inflated conditional effects from mixed models, which were also fitted to compare),[35] with the same variables as the linear mixed models and with clinic as a clustering variable. Missing data were not imputed.

Retention in the 12-week treatment programme was coded as weeks attended up to the point of continuous future non-attendance. Retention was compared between the groups using: 1) Fisher's exact test for retention in the trial overall, 2) a logistic GEE model for binary retention in opioid treatment at 12 weeks (with exchangeable correlation structure and 1000 repetition bootstrap standard errors), and 3) Kaplan-Meier and Cox regression analysis for time until continuous treatment non-attendance, with trial group and NHS stratification factor as predictors and clinic as a clustering variable.

Patient and Public Involvement.

A Service User Research Advisory Group (SURAG) was established before the trial commenced, comprising 14 service users with direct experience of receiving OAT. The group met once every six months to provide advice on the design, conduct and progress of the research. The SURAG helped significantly to make the trial procedures acceptable and aided implementation.

Results

Sample

Between September 2012 and September 2015, we randomly allocated 34 clinics (clusters) (of 61 considered) to three treatments (Figure 1; and see Supplementary material). Of the 34 clinics, all provided OAT, the majority were NHS (62%) and just under one half were within London (47%). Figure 1 shows that across 34 clusters, 789 patients were screened for eligibility, of whom 552 (31.8%) were consented and enrolled (between November 2012 and October 2015), a figure that fell short of our target of 660. One cluster did not recruit any participants. Although we recruited the target number of sites, we were unable to recruit 20 (and latterly 22) participants at all sites. Recruiting sites and participants at each site was challenging due to retendering of service contracts which affected many sites during the trial period. Two services were decommissioned during the trial.

Retention in the trial was high and comparable across trial arms. Eight participants actively withdrew from the trial (CM Abstinence = 4(2%); CM Attendance = 1(1%); and TAU = 3(2%);p=0.50).

For peer review only - http://bmjopen.bmj.com/site/about/guidelines.xhtml

Figure 1 - CONSORT diagram

Characteristics

Study participants were broadly representative of patients entering OAT in England (Table 1). They were mostly male (404;73%), white (435;79%) with a mean age of 38.2 years [SD 8.8]. They had first used opiates at the mean age of 23 years [SD 8], first injected at a mean age of 26 years [SD 8] and first received treatment at a mean age of 30 years [SD 8]. They had previously been in opiate treatment a median of 2 times [25th, 75th percentile 1, 4]. Over half had been in prison (291;53%). Fifty-seven per cent were prescribed methadone (315), 43% buprenorphine (237).

Table 1 - Baseline characteristics

All participants (552) self-reported using heroin in the month prior to interview (including two reporting using non-prescribed pharmaceutical heroin). Three quarters reported using crack (419;76%) while 23% used benzodiazepines (n=129). Half drank alcohol (53%;n=310) and nearly all used tobacco (531;96%) (Table 2 and see supplementary material).

Table 2 - Self-reported heroin use

There was reasonable balance across the three trial arms in terms of baseline socio-demographic, drug use, health, and treatment experience variables. However, there were more women in the CM Attendance group, fewer white individuals in the CM Abstinence group and a smaller proportion with insecure housing status in the CM Attendance group.

Abstinence from heroin at 12 weeks

Of the 2208 scheduled UDS (552 participants x 4 urines for weeks 9-12), 601 (27%) were not obtained due to non-compliance (refusal or DNA) and were imputed as positive (positive assumption), with the greatest number of these being in the TAU group. Additionally, 454 (21%) were treated as missing, including: 260 (12%) not obtained due to unable to contact, 77 (3%) not obtained due to clinic error, 15 (1%) unable to provide a sample, 55 (3%) in prison (47) in hospital (8), and for 10 (0.5%) the reason was missing. The percentage of UDS results missing by week and trial arm are shown in Table 3.

Table 3 Heroin – negative & positive UDS results

For peer review only - http://bmjopen.bmj.com/site/about/guidelines.xhtml

Results from the analyses with and without imputation were similar (Table 4), so results from imputed data are discussed. Participants in the CM Attendance group had statistically significantly greater odds of a heroin-negative urine at 9-12 weeks compared to those in the TAU group (OR=2.1; 95% CI 1.1 to 3.9;p=0.030). There were no statistically significant differences between the CM Abstinence and TAU group (OR=1.6; CI 0.9 to 3.0;p=0.146) or CM Attendance group (OR =1.3; CI:0.7 to 2.4;p=0.438).

Figure 2 shows the observed (dashed) and model predicted (solid) expected probabilities of a heroin-negative urine over the four weeks of the primary outcome period. Based on estimated proportions from model (1) in Table 4, a higher proportion of those in CM Attendance (62%) or CM Abstinence (57%) provided a negative-heroin urine sample at week 12 compared to TAU (48%). The intraclass correlations from the non-imputed data were 0.03 (95% CI 0.01 to 0.11) at the clinic level.

Table 4 - Primary outcome: odds of heroin-negative urine over weeks 9 to 12 & 21-24

Figure 2 - Probability of heroin-negative urine applying positive assumption over weeks 9-12

There was no moderation of trial intervention outcome by medication or treatment setting (Buprenorphine v Methadone ($\chi^2(2)$=1.89;p=.389); NHS or non NHS service (χ^2 (2)=4.96;p=0.084)) or by other or multiple drug use (Charlson Index of multiple drug use (χ^2 (2)=4.93;p=0.085)).

Abstinence from heroin at 24 weeks

There were no statistically significant differences among groups in heroin-abstinence for weeks 21-24 (Table 4).

Figure 3 shows the observed (dashed) and model (1) expected probabilities (solid) of a heroin-negative UDS over the four weeks of the follow-up period (weeks 21-24). The estimated proportions of those providing a negative-heroin UDS at 24 weeks from Model 1 in Figure 3 were similar across groups: CM Abstinence (44%); CM Attendance (45%) and TAU (37%).

Figure 3- Probability of heroin-negative urine applying positive assumption over weeks 21-24

Attendance

Figure 4 shows that 60% of those receiving CM Abstinence and 59% receiving CM Attendance attended their first appointment on time, compared with 46% of the TAU group. Eleven per cent of participants did not attend any weekly appointments (CM Abstinence = 11%; CM Attendance=7% and TAU=14%).

For peer review only - http://bmjopen.bmj.com/site/about/guidelines.xhtml

Figure 4 - Attendance at appointments

Attrition at each week was highest in the TAU group, with the proportion of participants attending appointments falling from 46% to 24% between weeks 1-12. In the CM Abstinence group there was a decline in attendance from 53% (week 4) down to 45% (week 5) with a steady decline thereafter to 33% attending at week 12. Attendance in the CM Attendance group remained consistent with a slight decline from 59% (week 1) to 51% (week 12). Most attendance was on-time (within 15 minutes) (Figure 4).

Retention in 12 weekly appointments

The proportions attending all of the 12-week appointments differed across the groups, with a higher proportion attending in CM Attendance as compared to CM Abstinence and TAU groups (56% vs 39% and 30%). Participants in the CM Attendance group had statistically significantly greater odds of full attendance compared with those in TAU (OR=3.1; 95%CI 2.0 to 4.6;p<0.001). There were no statistically significant differences between CM Abstinence and TAU (OR=1.5; 95%CI 0.9 to 2.4;p=0.099).

Participants in the CM Attendance group attended for longer than those in the CM Abstinence group or TAU group. Participants in the CM Abstinence group had statistically significantly greater risk of dropping out of the appointments before week 12 compared with those in TAU (Hazard Ratio (HR)=1.9;95% CI 1.5 to 2.5;p<0.001) and in CM Attendance groups (HR= 1.7; 95% CI 1.2 to 2.3;p=0.002) (Figure 5).

Figure 5 - Time to continuous appointment non-attendance

Clinical outcomes – Self-reported drug use

Self-reported heroin use reduced in all groups from nearly 100% (99.6%; 550) to 74% (291) at week 12, while the median number of days used reduced from 28 (21,30) to 4 (0,15). Self-reported use of crack and benzodiazepines reduced in the trial population from 76% to 54% and 23% to 16% respectively at 12 weeks. Self-reported alcohol and tobacco use remained similar (56% to 61% and 96% to 95% respectively). There were no significant differences in these outcomes between groups at 12 weeks (Tables 2 & 5; and see Supplementary material).

Table 5 - Self-reported heroin use

At 24-week follow-up, we found no difference in self-reported illicit drug use between groups. However, there was weak evidence of increased odds of alcohol use in the last 30 days in CM Attendance as compared to CM Abstinence (OR=1.9; 95%CI 1.0 to 3.5, p = 0.048), and evidence of

For peer review only - http://bmjopen.bmj.com/site/about/guidelines.xhtml

fewer days using tobacco in the last 30 days in TAU as compared to CM Abstinence (OR=2.1, 95%CI -0.3 to -3.9;p=0.024)(see Supplementary material). Mixed-effects models gave similar results.

Clinical outcomes – health and social

There were no differences between the groups in clinical outcomes at weeks 12 or 24 (for statistical tests see supplementary material). A similar therapeutic alliance score was reported by all groups at 4, 8 and 12 weeks (see Supplementary material).

Adherence to CM

Both CM Abstinence and CM Attendance achieved a score of "adequate" adherence to the CM manual. It is important to note these averages were on the lower end of the range of scores in this category (CM Abstinence=38.33%, SD=9.49 (range 24.6% to 56.3%); CM Attendance=44.15%, SD=17.07 (range 16.7% to 68.2%)).

Serious adverse events

There were 25 serious adverse events in 22 participants: CM Abstinence=10 in 9 participants; CM Attendance=8 in 7 participants; TAU=7 in 6 participants. There were 3 deaths (CM Abstinence=1; CM Attendance=2). None were related to the trial intervention.

Discussion

We found the adapted CM intervention targeted at attendance was moderately effective in encouraging heroin-abstinence compared with TAU. CM Abstinence was not more effective than TAU or CM Attendance

The UK's clinical guidance[2,15] which promotes the use of CM, was influenced by systematic reviews and meta-analysis conducted at least ten years ago which showed CM was effective in encouraging abstinence from a range of drugs.[9] Previous reviews examining the effectiveness of CM in promoting opioid-abstinence have been mixed [9,10,11,12,14] with the most recent systematic review and meta-analysis concluding there was no evidence of CM working better than control in encouraging abstinence from non-prescribed opiates during OAT.[14] Our findings concur with the most recent meta-analysis. Although adapting CM for ease of implementation in resource-poor settings may have caused it to lose some of its potential effectiveness.

When our adapted CM intervention was targeted at attendance only, it was superior to TAU in encouraging heroin-abstinence. It has been argued that clinical benefit arises from regular attendance at clinical appointments and overall retention in treatment.[36] Our findings suggest CM can improve attendance (when targeted at it) amongst populations who often prove challenging to engage with and retain in treatment and that the clinical benefit we observed supports such arguments.

The CM interventions we evaluated retained the main principles of CM, but modifications were made to ensure ease of implementation in UK settings with limited treatment budgets.

First, in the UK, resources are limited. Patients receiving OAT attend their service and receive UDS once a week at the start of treatment and less frequently thereafter. Previous studies exploring

For peer review only - http://bmjopen.bmj.com/site/about/guidelines.xhtml

views of CM among UK drug treatment staff and patients found that staff viewed the US frequency of urine testing to be too resource heavy for the UK settings and service users felt strongly that such a regime would act as a disincentive.[37] Hence, opiate-abstinence was monitored through once-weekly UDS rather than multiple tests per week. Opioids can only be detected up to 1-3 days after use so the UDS cannot confirm opioid abstinence over the week period, only provide evidence of abstinence over this briefer period, potentially reducing the impact of the reinforcer. As CM Abstinence had no effect on either UDS or self-reported heroin use, it is possible that CM Abstinence only encouraged abstinence in the 1–3 days before an appointment and a UDS test. While it may be argued that such behaviour change can be clinically significant to the individual patient, the absence of change in secondary outcomes suggests we should be cautious about inferring any significant clinical benefit.

Second, while the CM evidence base demonstrates the efficacy of escalating-value incentives,[8,9,10,11,12,13] we used a fixed-value incentive. In a previous UK study of CM targeted at hepatitis B vaccination, undertaken by the authors, we found escalating schedule was more difficult to implement and for staff to adhere to.[16] Employing escalating schedules might have risked unacceptable levels of protocol violations while also increasing the costs of the intervention, and limiting potential for translation into routine clinical practice. We used £10 vouchers – a figure our SURAG regarded as sufficient to encourage participants to attend and provide a UDS.

Third, it was critical that the CM schedule was acceptable to participants and supported by staff. Our SURAG argued that having to provide opiate-negative UDS would be challenging during titration and staff felt monitoring at this point may be undermining. In response, we introduced an initial 4-week priming phase in the CM Abstinence arm, during which participants received reinforcement for on-time provision of a UDS, irrespective of the result. The use of priming in CM ensures that patients experience receipt of the reinforcer early in treatment.[6] As participants were entering OAT and undergoing dose titration for their methadone/buprenorphine medication, priming provided an opportunity for participants to receive exposure to the incentive without the expectation that they needed to be heroin-abstinent during this period.

Fourth, vouchers were chosen as reinforcers as there is good evidence for their effectiveness[11,12] and costs were relatively low given the once-a-week frequency of reinforcement.

Adherence to CM as set out in the training manual was somewhat better in the CM Attendance group than CM Abstinence group but not significantly so. Movement of trained staff and supervisors away from participating services may well have impacted on adherence. Further exploration of the relationship between adherence and outcomes and the factors which may affect this (such as individual competence or service organisational factors) will help to clarify this.

Although CM has a strong evidence-base in achieving behaviour change, there is weaker evidence for behaviours sustained once the incentives are removed. Previous studies have found differing results with only a small treatment effect following discontinuation of incentives.[11] In this study, the reduction in heroin use was not sustained after discontinuation of CM. We are unable to conclude whether this was due to the cessation of CM or weekly appointments. These appointments were not routine practice in most drug services after 12 weeks but considered good clinical practice in the first 12 weeks of treatment.[38] Further research is needed to better understand how to maintain positive behaviour change, including whether the reinforcer needs to be maintained, given intermittently, or

For peer review only - http://bmjopen.bmj.com/site/about/guidelines.xhtml

tapered. We also need to explore the impact of the cessation, or reduced frequency of appointments after 12 weeks.

While there is a growing body of evidence for CM, it has not been widely implemented in UK drug treatment services. Similarly, the actual adoption and implementation of CM programmes in US community-based treatment programs (including those where most research on CM has been undertaken) is rare,[39] possibly due to limited staff capacity, resources and treatment budgets[6] though there are a some reports of successful implementation.[40]

Adapting CM for ease of implementation in resource-poor settings may have led to a loss of some of its potential effectiveness. Therefore, future research should consider how best to implement CM in routine practice both in UK and international contexts and should consider different models of delivery (including the use of mobile telephone technology).

References

[1] World Health Organization. Guidelines for the Psychosocially Assisted Pharmacological Treatment of Opioid Dependence. 2009 WHO Library

[2] National Institute for Care Excellence (NICE) Drug Misuse 2006

[3] Godfrey C, Stewart D, Gossop M. Economic analysis of costs and consequences of the treatment of drug misuse: 2-year outcome data from the National Treatment Outcome Research Study (NTORS). Addiction 2004; 99;6:697-707NTA 2012

[4] National Treatment Agency for Substance Misuse (NTA) From Access to Recovery: analysing six years of drug treatment data. NTA 2012.

[5] Skinner B.F. The Behavior of Organisms: An Experimental Analysis 1966, Skinner Foundation. Skinner, B. F. (1938). The behavior of organisms: an experimental analysis. Oxford, England: Appleton-Century.

[6] Nancy Petry's Contingency Management for substance abuse treatment: A Guide to Implementing This Evidence- Based practice (2012)

[7] Petry N.M., Alessi S,M., Marx J., Austin M., and Tardif M. Vouchers versus prizes: contingency management treatment of substance abuse in community settings. Journal of Consulting Clinical Psychology, 2005; 73: 1005-14.

[8] Lussier J.P, Heil S.H, Mongeon J.A, Badger G.J, Higgins S.T. A meta-analysis of voucher-based reinforcement therapy for substance use disorders. Addiction 2006; 101: 192-203.

[9] Prendergast M, Podus D, Finney J, Greenwall L, Roll J. Contingency management for treatment of substance use disorders: a meta-analysis. Addiction, 2006; 101: 1546-60.

[10] Higgins S.T, Sigmon S.C. Heil S.H, 2011 Contingency management in the treatment of substance use disorders: trends in the literature. In Ruiz P, Strain E (Eds.) Lowinson and Ruiz's Substance Abuse: A comprehensive Textbook. Lippincott Williams and Wilkins, Philadelphia PA, pp 603-621.

For peer review only - http://bmjopen.bmj.com/site/about/guidelines.xhtml

[11 Davis D.R, Kurti A.N, Skelly J.M. Redner R, White T.J. and Higgins S.T. A review of the literature on contingency management in the treatment of substance misuse disorders 2009-2014. Preventive Medicine. 2016.

[12] European Monitoring Centre for Drugs and Drug Addiction (2016), How can contingency management support treatment for substance use disorders? A systematic review, EMCDDA Papers, Publications Office of the European Union, Luxembourg.

[13] Amato L, Minozzi S, Davoli M, Vecchiet S (2011) Cochrane Database of Systematic Reviews: Psychosocial combined with agonist maintenance treatments versus agonist maintenance treatments alone for treatment of opioid dependence

[14] Ainscough TS, McNeill A, Strang J, Calder R, Brose L... Contingency Management interventions for non-prescribed drug use during treatment for opiate addiction: A systematic review and meta-analysis. 2017. Drug Alcohol Depend. 2017 Sep 1;178:318-339.

[15] National Collaborating Centre for Mental Health. Drug Misuse: psychosocial Interventions. London: British Psychological Society and The Royal College of psychiatrists: (2008).

[16] Weaver T, Metrebian N, Hellier J, Pilling S, Charles V, Little N, Poovendran D, Mitcheson L, Ryan F, Bowden-Jones O, Dunn J, Glasper A, Finch E and Strang J. Use of contingency management incentives to improve completion of hepatitis B vaccination in people undergoing treatment for heroin dependence : a cluster randomised trial. The Lancet 2014; 384: 153-63.

[17] Rafia R, Dodd P.J, Brennan A, Meier P.S, Hope V.D, Ncube F, Byford S, Tie H, Metrebian N, Hellier J, Weaver T, and Strang J. An economic evaluation of contingency management for completion of hepatitis B vaccination in those on treatment for opiate dependence Addiction.2016:111(9):1616-1627.

[18] Metrebian N, Weaver T, Pilling S, Goldmith K, Hellier J, Pickles A, Byford S, Shearer J et al, Positive Reinforcement targeting Abstinence In Substance misuse (PRAISe): Cluster RCT & Process Evaluation of Contingency Management. NIHR Library. Contemporary Clinical Trials 2018; 71 :124-132

[19] Metrebian N, Weaver T, Pilling S, Goldsmith K, Hellier J, Pickles A, Shearer J, Byford S, Rafia R, Dodd P, and Strang J. Developing a UK Evidence Base for Contingency Management in Addiction Treatment: Incentive-based interventions to improve treatments to reduce drug use and associated harms NIHR Programme Grants for Applied Research Programme. Final Report.

[20] NTA (2006) Models of Care for the treatment of drug misusers: Update 2006. London: NTA.

[21] Carroll KM, Nich C, Sifry RL, Nuro KF, Frankforter TL, Ball SA, Fenton L & Rounsaville BJ. A General system for evaluating therapist adherence and competence in psychotherapy research in the addictions, Drug and Alcohol Dependence 2000; 1; 57(3):225-38.)

[22] Herscell et al The role of the therapist training in the implementation of psychosocial treatments: a review and critique with recommendi+ons . Clinical psychology Review 2010; 30(4), 448-466

For peer review only - http://bmjopen.bmj.com/site/about/guidelines.xhtml

[23]. Darke, S., Heather, N., Hall, W., Ward, J. & Wodak, A. Estimating drug consumption in opioid users: Reliability and validity of a "recent use" episodes method. British Journal of Addiction, 1991; 86, 1311-1316.

[24] Saunders, J.B., Aasland, O.G., Babor, T.F., de la Fuente, J.R. and Grant, M. Development of the Alcohol Use Disorders Identification Test (AUDIT): WHO collaborative project on early detection of persons with harmful alcohol consumption. II. Addiction, 1993; 88:791-804.

[25]. Darke S, Hall W, Wodak A, Heather N, Ward J. Development and validation of a multidimensional instrument for assessing outcome of treatment among opiate users: the Opiate Treatment Index Addiction; 1992; 87;5:733–74217.

[26]. SF-36 version II. QualityMetric Incorporated

[27]. Zigmond AS, Snaith P. The hospital anxiety and depression scale. Acta Psychiatr Scand. 1983; 67:361–370.

[28] Cahill, T. J, & Stiles, W, Barkham, M, Hardy, G Stone, G & Agnew-Davies, R & Unsworth, G. Two short forms of the Agnew Relationship Measure: the ARM-5 and ARM-12. Psychotherapy Research : Journal of the Society for Psychotherapy Research; 2011; 22: 241-55.

[29] Madden, G. J., Petry, N. M., Badger, G. J., & Bickel, W. K. (1997). Impulsive and self-control choices in opioid-dependent patients and non-drug-using control participants: Drug and monetary rewards. Experimental and Clinical Psychopharmacology, 1997; 5, 256-262.

[30] Levesque C.S., Williams G.C., Elliot D, Pickering M.A., Bodenhamer B. and Finley P.J. Validating the theoretical structure of the Treatment Self-Regulation Questionnaire (TSRQ) across three different health behaviours Health Education Research 2007; 22(5): 691–702

[31] Byford, S., et al., Cost-effectiveness of injectable opioid treatment v. oral methadone for chronic heroin addiction. British Journal of Psychiatry, 2013. 203: p. 341-349.

[32] EuroQol Group. EuroQol—a new facility for the measurement of health-related quality of life. Health Policy. 1990; 16: 199–208

[33] Kessler R, et al The World Health Organisation Health and Work Performance Questionnaire. (HPQ), J. Occupat. Environ. Med. 2003, 45 (2): 156-174.

[34] Strang J, Metrebian N, Lintzeris N, Potts L, Carnwath T, Mayet S, et al. Supervised injectable heroin or injectable methadone versus optimised oral methadone as treatment for chronic heroin addicts in England after persistent failure in orthodox treatment (RIOTT) : a randomised trial. Lancet 2010, 375: 1885-95

[35] F B Hu, J Goldberg, D Hedeker, B R Flay, M A Pentz. Comparison of Population-Averaged and Subject-Specific Approaches for Analyzing Repeated Binary Outcomes, Am J Epidemiol. 1998 Apr 1;147(7):694-703. doi: 10.1093/oxfordjournals.aje.a009511

[36] Department of Health and devolved administrations: Drug misuse and dependence: UK Guidelines on clinical management Drug misuse and dependence 2007. Department of Health.

For peer review only

[37] Sinclair, J. M. A., Burton, A., Ashcroft, R. & Priebe, S. 2011. Clinician and service user perceptions of implementing contingency management: A focus group study. Drug and Alcohol Dependence., 119, 56-63.

[38] Hartzler B, Lash SJ, Roll JM. Contingency management in substance abuse treatment: A structured review of the evidence for its transportability. Drug and Alcohol Dependence. 2012;122(1):1-10.

[39] Kropp F., Lewis D., Winhusen T. The effectiveness of ultra-low magnitude reinforcers: Findings from a "real-world" application of contingency management. Journal of Substance Abuse Treatment, 2017;72: 111-116.

[40] DePhilippis D, Petry N, Bonn-Miller M, Rosenbach S, Mckay J.R. University of PennsylThe national implementation of Contingency Management (CM) in the Department of Veterans Affairs: Attendance at CM sessions and substance use outcomes, Drug and Alcohol Dependence 2018.185:367-373

Acknowledgements

We would like to thank all the drug service staff and service users who participated in this trial. We also gratefully acknowledge the support and guidance of members of our Trial Steering Committee (Simon Coulton [Chair], Christopher Whiteley, and Soraya Mayet); our Data Management and Ethics Committee (Louise Sell [Chair], Anne R Lingford-Hughes, and Zoe Hoare) and our Service User Research Advisory Group and Anthea Martin for facilitating this group. Also, we would like to acknowledge the guidance and support we received from the late Prof Nancy Petry who collaborated on this trial.

Funding statement

This paper presents independent research funded by the National Institute for Health Research (NIHR) under its Programme Grants for Applied Research Programme (grant reference number RP-PG-0707-10149). The views expressed are those of the authors and not necessarily those of the National Health Service, the NIHR, or the Department of Health. The research was also part funded by the National Institute for Health Research (NIHR) Biomedical Research Centre at South London and Maudsley NHS Foundation Trust and King's College London.

Ethical Review

The PRAISE (Positive Reinforcement targeting Abstinence In Substance misusE) trial was reviewed at the NRES Committee South East Coast-Surrey 12/LO/0910 and received a favourable opinion on the 25/07/2012.

The trial is prospectively registered, ISRCTN 01591254.

Competing interests' statement

JS, SP and LM have contributed to UK guidelines on the potential role of contingency management in the management of opioid addiction (NICE, 2007; convened by SP, chaired by JS), SP receives

For peer review only - http://bmjopen.bmj.com/site/about/guidelines.xhtml

funding from NICE for the production of clinical guidelines. JS has chaired the broader-scope pan-UK working group preparing the 2017 and 2007 Orange Guidelines for the UK Departments of Health and Social Care, providing guidance on management and treatment of drug dependence and misuse, including guidance on possible inclusion of contingency management. LM and ED contributed to these guidelines.

JS is a researcher and clinician who, through his university, has worked with various pharmaceutical companies to identify new or improved treatments and his employer (King's College London) has received grants, travel costs and/or consultancy payments from companies including, past 3 years, Indivior, Mundipharma, Camurus, Molteni Farma, Accord. JS has also worked with various drug policy organisations and advisory bodies including the Society for the Study of Addiction (SSA), and the European Monitoring Centre for Drugs and Drug Addiction (EMCDDA).

JS and KG are supported by the National Institute for Health Research (NIHR) Biomedical Research Centre for Mental Health at South London and Maudsley NHS Foundation Trust and King's College London. JS is an NIHR Senior Investigator.

For a fuller account, see JS's web-page at http://www.kcl.ac.uk/ioppn/depts/addictions/people/hod.aspx

NM is involved in research project funded by Pharmaceutical company MundiPharma.

LM has been in receipt of an untied educational grant from Indivior for the ARC study which incorporated the use of CM in the intervention arm. LM is currently involved in Pharmaceutical company (Indivior) funded study – the EXPO trial which will incorporate the use of CM as part of the psychosocial intervention. LM has a paid secondment to PHE to advise on best practice psychological interventions in drug and alcohol treatment. ED has recently been appointed as the government's Drug Recovery Champion.

Authors contribution

Contributors: As the corresponding author – I have had full access to all the data in the study and had final responsibility for the decision to submit for publication.

Concept, original design (JSt, TW, SP, SB); trial administration and management (NM, TW); treatment development, training and supervision (SP, LM); Statistical analysis (KG, JH, AP, JS, SB);, implementation lead medical clinicians (PB, NB, OBJ, ED, JD, AG, EF, SF,); data management (NM & FH), data handling and entry (SA, JB, CB, EB, VC, CD, RD, CG, FH, NL, HMK, FM, JM, JM, RP, DP, EP); data interpretation(NM, TW, KG, JSt); First draft manuscript (NM, KG, TW); further work on manuscript (NM, TW, KG, SP, JSt, SB, JS). All authors read and approved the final manuscript.

Data sharing statement

Data collected for the study, including individual participant data and a data dictionary defining each field in the set, will be made available to others. These data will include: deidentified participant data, data dictionary, study protocol, statistical analysis plan, and informed consent form. These data will be available with publication and on application to Dr Nicola Metrebian. Access criteria for obtaining data will include approval by Chief Investigator (Prof Sir John Strang), approval of study proposal, and with a signed data access agreement.

For peer review only

For peer review only

For peer review only - http://bmjopen.bmj.com/site/about/guidelines.xhtml

TABLES

For peer review only

For peer review only - http://bmjopen.bmj.com/site/about/guidelines.xhtml

Table 1: Baseline characteristics by treatment group.

		CM Abstinence			CM Attendance			TAU			TOTAL		
		N	Mean / or median %	SD or IQR	N	Mean / or median %	SD or IQR	N	Mean / or median %	SD or IQR	N	Mean / or median %	SD or IQR
**DEMOGRAPHICS**													
Age at Baseline	(years) (mean)	205	38.39	8.76	174	38.02	8.48	173	38.08	9.30	552	38.18	8.83
Gender	male	160	78.05		118	67.82		126	72.83		404	73.19	
	female	45	21.95		56	32.18		47	27.17		148	26.81	
Ethnicity	White	145	70.73		145	83.33		145	83.82		435	78.80	
	Black	15	7.32		5	2.87		13	7.51		33	5.98	
	Asian	23	11.22		11	6.32		8	4.62		42	7.61	
	mixed	18	8.78		13	7.47		6	3.47		37	6.70	
	not done	4	1.95		0	0		1	0.58		5	0.91	
Employment	Employed	13	6.34		27	15.52		22	12.72		62	11.23	
	Unemployed/sickness	190	92.68		146	83.91		145	83.82		481	87.14	
	Student	0	0		0	0		1	0.58		1	0.18	
	House wife/husband	2	0.98		0	0		1	0.58		3	0.54	
	Retired	0	0		0	0		2	1.16		2	0.36	
	Other	0	0		1	0.57		2	1.16		3	0.54	
Normally live with	Partner/spouse	33	16.10		43	24.71		27	15.61		103	18.66	
	Friends	16	7.80		11	6.32		14	8.09		41	7.43	

For peer review only - http://bmjopen.bmj.com/site/about/guidelines.xhtml

For peer review only

	Alone	89	43.41		75	43.10		82	47.40		246	44.57	
	Family	45	21.95		31	17.82		31	17.92		107	19.38	
	Other	21	10.24		14	8.05		19	10.98		54	9.78	
	Unknown	1	0.49		0	0.00		0	0.00		1	0.18	
Accommodation	Owner occupier	22	10.73		13	7.47		8	4.62		43	7.79	
	Rented private	33	16.10		44	25.29		34	19.65		111	20.11	
	Rented (LA, HA)	75	36.59		75	43.10		73	42.20		223	40.40	
	Living with parent	23	11.22		15	8.62		14	8.09		52	9.42	
	B&B/hotel	2	0.98		3	1.72		2	1.16		7	1.27	
	Hostel	28	13.66		12	6.90		10	5.78		50	9.06	
	NFA (living on the	21	10.24		12	6.90		31	17.92		64	11.59	
	Other	0	0		0	0		1	0.58		1	0.18	
	Unknown	1	0.49		0	0		0	0		1	0.18	
Prison	No	95	47.03		80	46.24		81	47.09		256	46.80	
	Yes	107	52.97		93	53.76		91	52.91		291	53.20	
Times on remand		105	6.28	8.69	92	5.27	5.59	88	5.10	7.59	285	5.59	7.45
**DRUG USE HISTORY**													
Age first used opiates	(years)	200	23.39	8.01	169	23.67	7.65	173	23.35	7.48	542	23.46	7.72
Age first regular use	(years)	200	25.30	8.18	173	26.02	7.90	173	26.28	7.92	546	25.84	8.01
Age first injected	(years)	121	26.10	7.85	92	26.51	8.48	108	26.32	7.46	321	26.29	7.88
Age first received help	(years)	193	30.23	8.36	170	29.45	7.56	167	30.00	8.54	530	29.91	8.16
**DRUG TREATMENT**													
Treatment	Methadone	119	58.05		106	60.92		90	52.02		315	57.07	
	Buprenorphine (Sub)	86	41.95		68	39.08		83	47.98		237	42.93	

For peer review only - http://bmjopen.bmj.com/site/about/guidelines.xhtml

Treatment dose	Methadone	119	34.45	13.05	103	31.41	7.49	90	34.32	11.90	312	33.41	11.20
(mean mg)	Buprenorphine (Sub)	85	6.87	4.67	68	5.90	3.15	81	6.56	3.20	234	6.48	3.80
Median times in opiate treatment		198	2	1 to 4	172	2	1 to 3	171	2	1 to 5	541	2	1 to 4

Table 2 **Opiate Treatment Index (OPI) measures of Heroin use in last 30 days measured at Baseline, 12 and 24 weeks.**

	**CM Abstinence n=205**	**CM Attendance n = 174**	**TAU n= 173**	**Total n = 552**
**Summary statistics for binary Yes/No to 'use in the past 30 days'?** (Missing shown but n% proportions calculated disregarding missing.)	N (%)	N (%)	N (%)	N (%)
**Baseline**				
Used heroin in last 30 days	205 (100)	173 (99)	172 (99)	550 (99.6)
Did not use heroin in last 30 days	0 (0)	1 (1)	1 (1)	2 (0.4)
Missing	*0*	*0*	*0*	*0*
**12 weeks**				
Used heroin in last 30 days	96 (73)	111 (78)	84 (72)	291 (74)
Did not use heroin in last 30 days	36 (27)	32 (22)	32 (28)	100 (26)
Missing	*73*	*31*	*57*	*161*
**24 weeks**				
Used heroin in last 30 days	97 (79)	85 (78)	66 (75)	248 (78)
Did not use heroin in last 30 days	26 (21)	24 (22)	22 (25)	72 (23)
Missing	*82*	*65*	*85*	*232*
**Number of days taking heroin in last 30 days**				
**Baseline**				
n	204	173	173	550

For peer review only

For peer review only - http://bmjopen.bmj.com/site/about/guidelines.xhtml

Number missing	1	1	0	2
Proportion missing	0.49	0.57	0	0.36
Median (25th, 75th percentile)	28.00 (22.00, 30.00)	27.00 (21.00, 30.00)	28.00 (21.00, 30.00)	28.00 (21.00, 30.00)
**12 weeks**				
n	132	143	116	391
Number missing	73	31	57	161
Proportion missing	35.61	17.82	32.95	29.17
Median (25th, 75th percentile)	4.00 (0.00, 15.00)	5.00 (1.00, 13.00)	4.00 (0.00, 15.00)	4.00 (0.00, 15.00)
**24 weeks**				
n	123	108	88	319
Number missing	82	66	85	233
Proportion missing	40.00	37.93	49.13	42.21
Median (25th, 75th percentile)	5.00 (1.00, 15.00)	4.50 (1.00, 15.00)	3.50 (0.50, 20.00)	4.00 (1.00, 15.00)

Table 3 Urine Results - Heroin

(using opiates and 6MAM variables in combination + recoding missing urines to positive if participant DNA or refused to provide = positive assumption)

	Week 9				Week 10				Week 11				Week 12			
	CM Abstinence	CM Attendanc	TAU	TOTAL	CM Abstinence	CM Attendanc	TAU	TOTAL	CM Abstinence	CM Attendanc	TAU	TOTAL	CM Abstinence	CM Attendanc	TAU	TOTAL
Neg	73	58	44	175	59	67	44	170	70	78	51	199	82	95	62	239
	35.61	33.33	25.43	31.70	28.78	38.51	25.43	30.80	34.15	44.83	29.48	36.05	40.00	54.60	35.84	43.30
Pos	77	78	86	241	96	71	85	252	80	67	83	230	69	56	73	198
	37.56	44.83	49.71	43.66	46.83	40.80	49.13	45.65	39.02	38.51	47.98	41.67	33.66	32.18	42.20	35.87

For peer review only - http://bmjopen.bmj.com/site/about/guidelines.xhtml

MISSING	55	38	43	136	50	36	44	130	55	29	39	123	54	23	38	115
	26.83	21.84	24.86	24.64	24.39	20.69	25.43	23.55	26.83	16.67	22.54	22.28	26.34	13.22	21.97	20.83
Total	205	174	173	552	205	174	173	552	205	174	173	552	205	174	173	552

	Week 21				Week 22				Week 23				Week 24			
	CM Abstinence	CM Attendanc	TAU	TOTAL	CM Abstinence	CM Attendanc	TAU	TOTAL	CM Abstinence	CM Attendanc	TAU	TOTAL	CM Abstinence	CM Attendanc	TAU	TOTAL
Neg	63	68	45	176	51	36	30	117	47	40	36	123	56	52	49	157
	30.73	39.08	26.01	31.88	24.88	20.69	17.34	21.20	22.93	22.99	20.81	22.28	27.32	29.89	28.32	28.44
Pos	75	56	73	204	87	80	87	254	86	81	78	245	75	72	66	213
	36.59	32.18	42.20	36.96	42.44	45.98	50.29	46.01	41.95	46.55	45.09	44.38	36.59	41.38	38.15	38.59
MISSING	67	50	55	172	67	58	56	181	72	53	59	184	74	50	58	182
	32.68	28.74	31.79	31.16	32.68	33.33	32.37	32.79	35.12	30.46	34.10	33.33	36.10	28.74	33.53	32.97
Total	205	174	173	552	205	174	173	552	205	174	173	552	205	174	173	552

For peer review only

Table 4: Primary outcome analysis: Odds of subjects providing a heroin-negative urine over weeks 9 to 12 and 21 to 24

	Contrast	Odds Ratio	95% CI		P-Value
**Weeks 9 to 12**					
(1) Outcome: repeated binary heroin result with refused & DNA=Positive sample (N= 483, no multiple imputation)	CM Abstinence vs TAU	1.67	0.89	3.14	.114
	CM Attendance vs TAU*	2.19	1.15	4.15	.017
	CM Attendance vs CM Abstinence	1.31	0.71	2.44	.391
(2) Outcome: repeated binary heroin result with refused & DNA=positive sample (i.e. Analysis 1) and remaining missing multiply imputed (N=552, using multiple imputation)	CM Abstinence vs TAU	1.59	0.85	3.01	.146
	CM Attendance vs TAU*	2.05	1.07	3.91	.030
	CM Attendance vs CM Abstinence	1.29	0.68	2.41	.438
**Weeks 21 to 24**					
(1) Repeated binary analysis for weeks 21, 22, 23, 24 with refused & DNA=0 (N= 409, no multiple imputation)	CM Abstinence vs TAU	1.71	0.72	4.09	.225
	CM Attendance vs TAU	1.66	0.69	4.00	.261
	CM Attendance vs CM Abstinence	0.97	0.41	2.28	.939
(2) Analysis (1) with remaining missing multiply imputed (N=552, using multiple imputation)	CM Abstinence vs TAU	1.46	0.66	3.26	.352
	CM Attendance vs TAU	1.29	0.57	2.92	.543
	CM Attendance vs CM Abstinence	0.89	0.40	1.93	.751

**significant at 0.05*

For peer review only

Table 5 **Odds ratios for Opiate Treatment Index (OPI) measures of heroin use in the last 30 days assessed at 12 and 24 weeks: Use in last 30 days (binary) and N of days used.**

	Treatment group difference estimate	Lower confidence limit	Upper confidence limit	p-value
**HEROIN Use in last 30 days Y/N across groups – GEE model results**				
12 weeks CM Attendance v CM Abstinence	1.28	0.77	2.14	0.336
12 weeks TAU v CM Abstinence	0.98	0.56	1.70	0.936
12 weeks TAU v CM Attendance	0.76	0.47	1.23	0.268
24 weeks CM Attendance v CM Abstinence	0.95	0.51	1.78	0.876
24 weeks TAU v CM Abstinence	0.80	0.40	1.63	0.545
24 weeks TAU v CM Attendance	0.85	0.42	1.69	0.633
**Mean differences N of days used in last 30 days across groups**				
12 weeks CM Attendance v CM Abstinence	-0.42	-3.11	2.27	0.760
12 weeks TAU v CM Abstinence	0.02	-2.77	2.80	0.990
12 weeks TAU v CM Attendance	0.44	-2.33	3.21	0.757
24 weeks CM Attendance v CM Abstinence	-0.25	-3.06	2.56	0.859
24 weeks TAU v CM Abstinence	-0.13	-3.06	2.80	0.931
24 weeks TAU v CM Attendance	0.13	-2.85	3.10	0.934

For peer review only

For peer review only - http://bmjopen.bmj.com/site/about/guidelines.xhtml

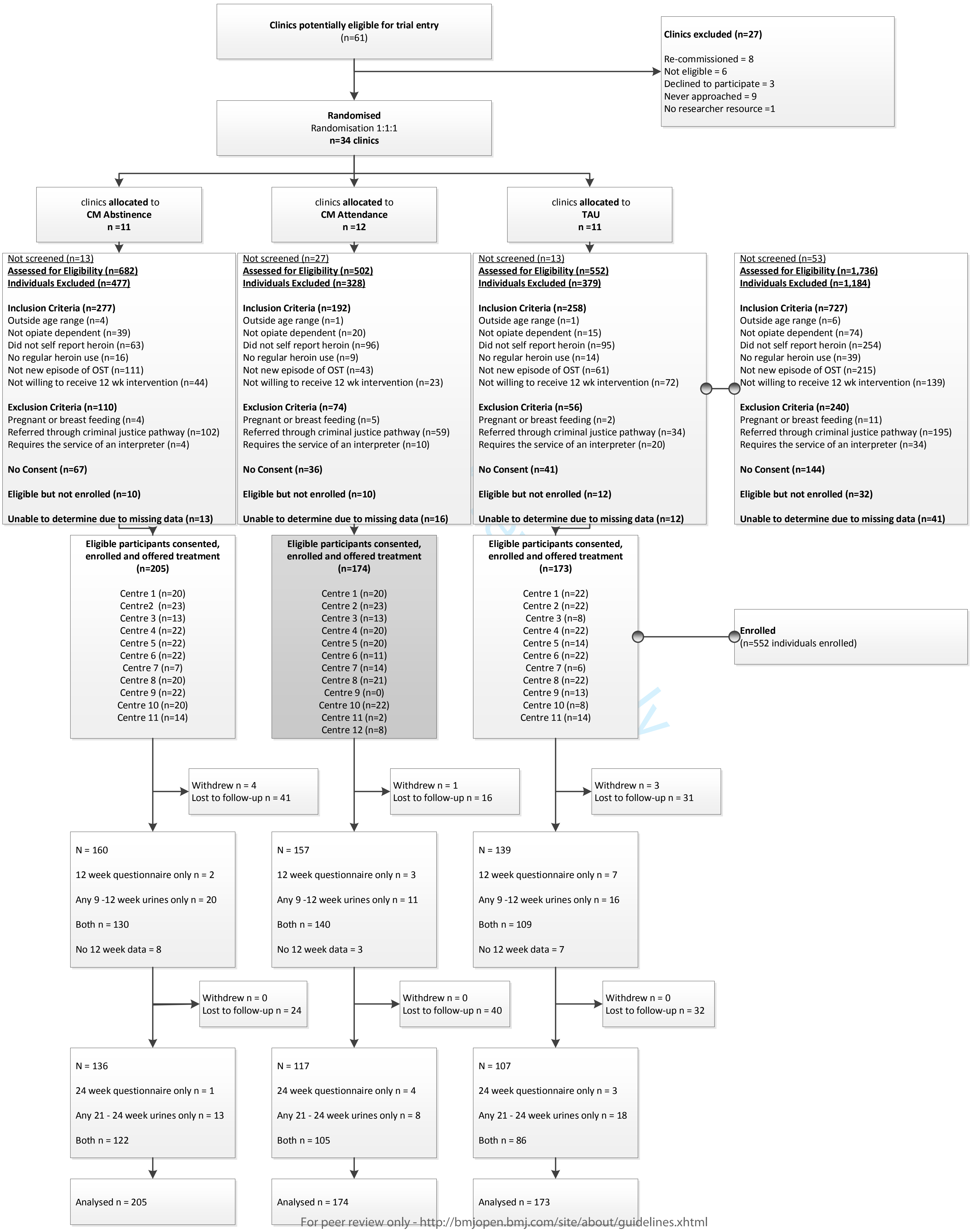

For peer review only - http://bmjopen.bmj.com/site/about/guidelines.xhtml

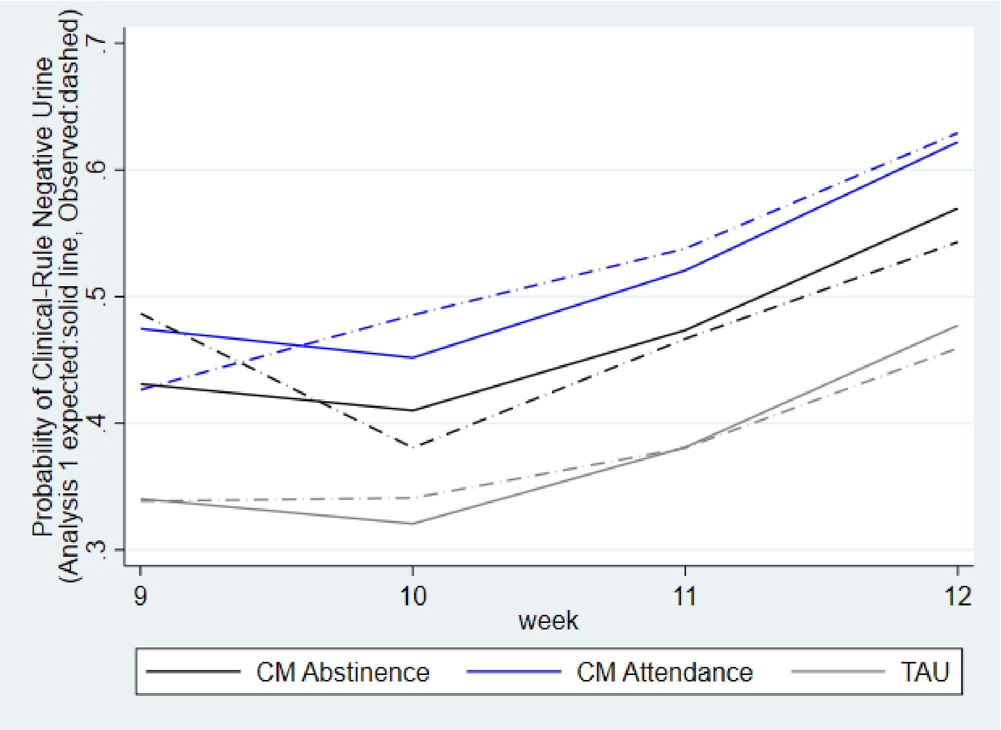

Figure 2

218x158mm (72 x 72 DPI)

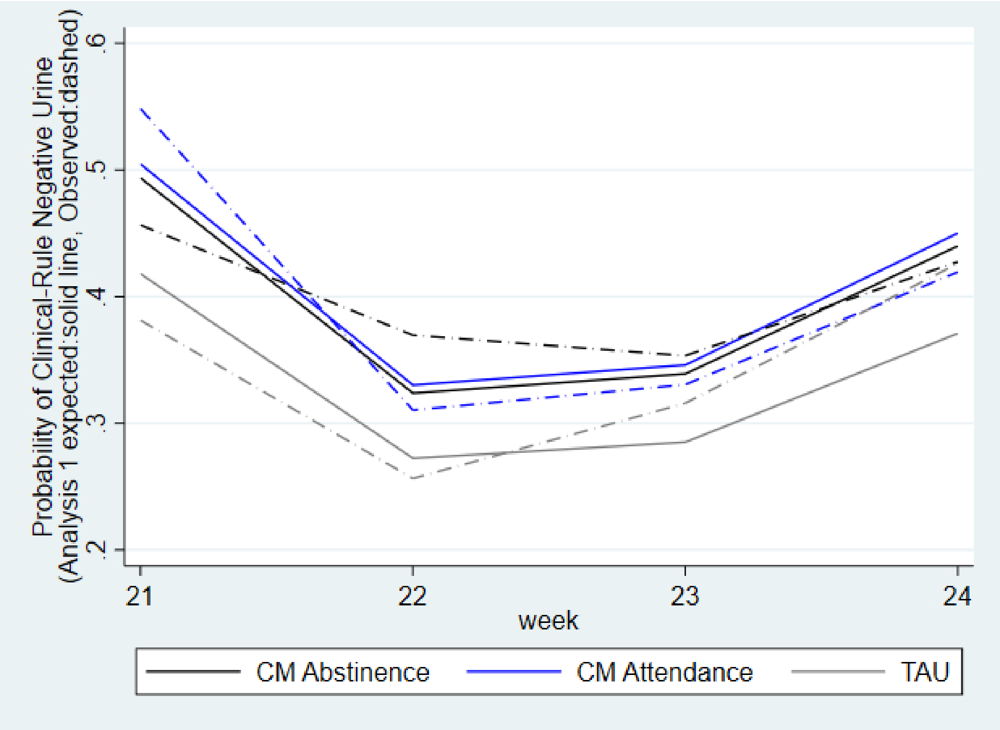

Figure 3

218x158mm (72 x 72 DPI)

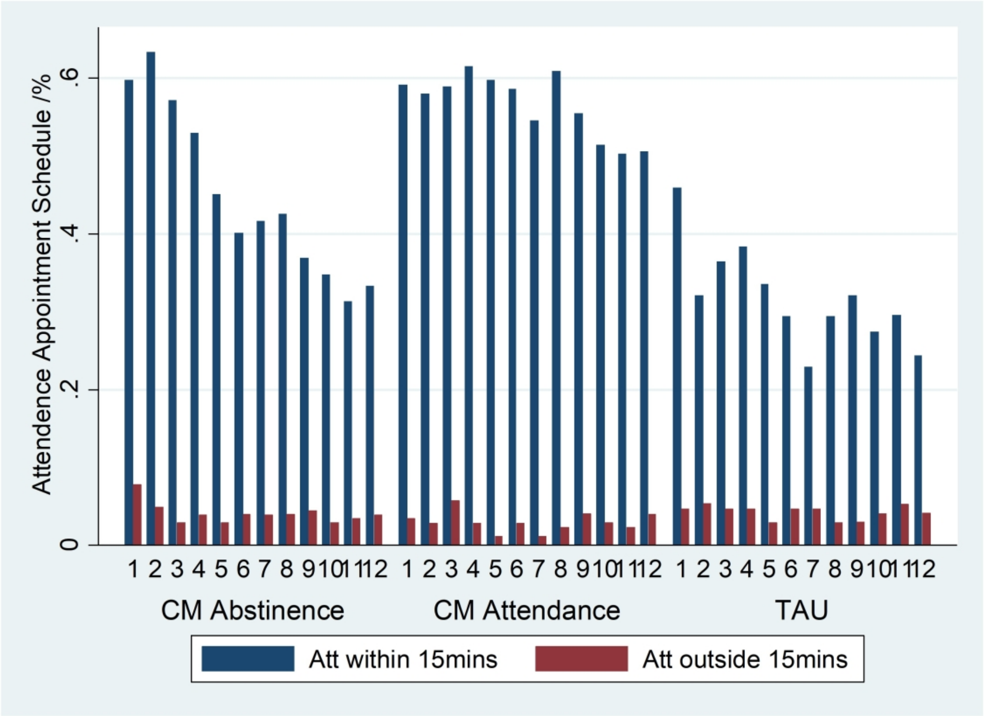
Attendence Appointment Schedule /%
0
.2
.4
.6
1 2 3 4 5 6 7 8 9 10 11 12
1 2 3 4 5 6 7 8 9 10 11 12
1 2 3 4 5 6 7 8 9 10 11 12
CM Abstinence
CM Attendance
TAU
Att within 15mins
Att outside 15mins

Figure 4

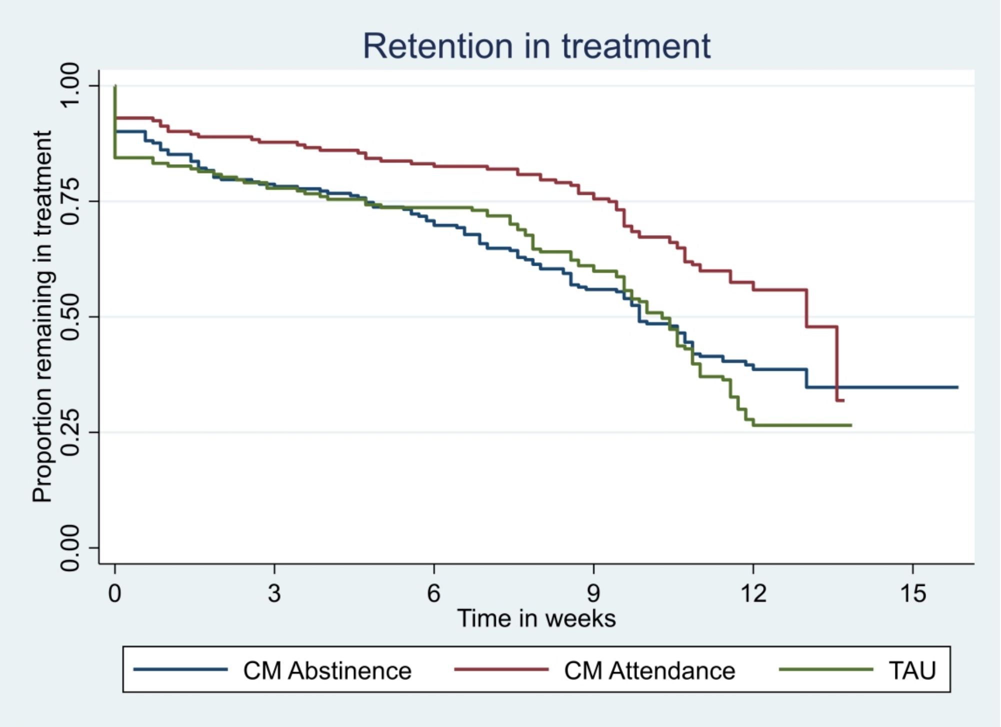

Figure 5

873x635mm (96 x 96 DPI)

Metrebian, Weaver & Goldsmith et al. Using a pragmatically adapted, low-cost, contingency management intervention to promote heroin abstinence in individuals undergoing treatment for heroin-use disorder in UK drug services (PRAISE): a cluster randomised trial

Supplementary material

For peer review only

Table 1. Clinic characteristics by Arm

		CM Abstinence			CM Attendance			TAU			TOTAL		
		N	Mean / or median %	SD or IQR	N	Mean / or median %	SD or IQR	N	Mean / or median %	SD or IQR	N	Mean / or median %	SD or IQR
Clinic type<br>N (%) NHS		6	55		7	58		8	73		21	62	
Clinic location<br>N (%) London		7	63		3	25		6	55		16	47	
Birmingham		2	18		2	17		1	9		5	15	
Dudley & Walsall		0	0		1	8		0	0		1	3	
Herts		1	9		4	33		1	9		6	18	
South Essex		1	9		1	8		0	0		2	6	
Sussex		0	0		1	8		2	18		3	9	
Avon & Wiltshire		0	0		0	0		1	9		1	3	
Clinic total		11	100		12	100		11	100		34	100	

For peer review only - http://bmjopen.bmj.com/site/about/guidelines.xhtml

For peer review only

Table 2 **Opiate Treatment Index (OPI) measures of substance use in last 30 days. Use in last 30 days (binary) and N of days used.** (Missing shown but n% proportions calculated disregarding missing

	CM Abstinence n=205	CM Attendance n = 174	TAU n= 173	Total n = 552
**CRACK**				
**Baseline**				
Used crack in last 30 days	161 (79)	128 (74)	130 (75)	419 (76)
Did not use crack in last 30 days	44 (22)	46 (26)	43 (25)	133 (24)
Missing	*0*	*0*	*0*	*0*
**12 weeks**				
Used crack in last 30 days	75 (57)	73 (52)	62 (53)	210 (54)
Did not use crack in last 30 days	57 (43)	68 (48)	54 (47)	179 (46)
Missing	*73*	*33*	*57*	*163*
**24 weeks**				
Used crack in last 30 days	78 (63)	56 (51)	52 (58)	186 (58)
Did not use crack in last 30 days	45 (37)	53 (49)	37 (42)	135 (42)
Missing	*82*	*65*	*84*	*231*
Number of days taking crack in last 30 days				
**Baseline**				
n	203	174	172	549
Number missing	2	0	1	3
Proportion missing	0.98	0	0.58	0.54
Median (25th, 75th percentile)	10.00 (1.00, 26.00)	7.00 (0.00, 20.00)	5.00 (0.50, 24.50)	7.00 (1.00, 24.00)
**12 weeks**				
n	132	140	116	388
Number missing	73	34	57	164
Proportion missing	35.61	19.54	32.95	29.71

For peer review only

Median (25th, 75th percentile)	1.00 (0.00, 10.00)	1.00 (0.00, 4.00)	1.00 (0.00, 4.00)	1.00 (0.00, 5.00)
**24 weeks**				
n	123	109	89	321
Number missing	82	65	84	231
Proportion missing	40	37.36	48.55	41.85
Median (25th, 75th percentile)	2.00 (0.00, 6.00)	1.00 (0.00, 4.00)	1.00 (0.00, 4.00)	1.00 (0.00, 5.00)
**BENZODIAZEPENES**				
**Baseline**				
Used benzos in last 30 days	47 (23)	35 (20)	47 (27)	129 (23)
Did not use benzos in last 30 days	158 (77)	138 (80)	126 (73)	422 (77)
Missing	*0*	*1*	*0*	*1*
**12 weeks**				
Used benzos in last 30 days	20 (15)	16 (11)	26 (22)	62 (16)
Did not use benzos in last 30 days	111 (85)	125 (89)	90 (78)	326 (84)
Missing	*74*	*33*	*57*	*164*
**24 weeks**				
Used benzos in last 30 days	20 (16)	9 (8)	16 (18)	45 (14)
Did not use benzos in last 30 days	103 (84)	100 (92)	73 (82)	276 (86)
Missing	*82*	*65*	*84*	*231*
Number of days taking benzodiazepines in last 30 days				
**Baseline**				
n	205	172	173	550
Number missing	0	2	0	2
Proportion missing	0	1.15	0	0.36
Median (25th, 75th percentile)	0.00 (0.00, 0.00)	0.00 (0.00, 0.00)	0.00 (0.00, 1.00)	0.00 (0.00, 0.00)
**12 weeks**				
n	131	139	116	386
Number missing	74	35	57	166
Proportion missing	36.10	20.11	32.95	30.07

For peer review only

Median (25th, 75th percentile)	0.00 (0.00, 0.00)	0.00 (0.00, 0.00)	0.00 (0.00, 0.00)	0.00 (0.00, 0.00)
**24 weeks**				
n	123	109	89	321
Number missing	82	65	84	231
Proportion missing	40.00	37.36	48.55	41.85
Median (25th, 75th percentile)	0.00 (0.00, 0.00)	0.00 (0.00, 0.00)	0.00 (0.00, 0.00)	0.00 (0.00, 0.00)
**ALCOHOL**				
**Baseline**				
Used alcohol in last 30 days	117 (57)	93 (53)	100 (58)	310 (56)
No alcohol use in last 30 days	88 (43)	81 (47)	73 (42)	242 (44)
Missing	*0*	*0*	*0*	*0*
**12 weeks**				
Used alcohol in last 30 days	77 (58)	83 (59)	76 (66)	236 (61)
No alcohol use in last 30 days	55 (42)	59 (42)	40 (35)	154 (40)
Missing	*73*	*32*	*57*	*162*
**24 weeks**				
Used alcohol in last 30 days	70 (57)	67 (62)	51 (57)	188 (59)
No alcohol use in last 30 days	53 (43)	42 (39)	38 (43)	133 (41)
Missing	*82*	*65*	*84*	*231*
Number of days taking alcohol in last 30 days				
**Baseline**				
n	204	172	172	548
Number missing	1	2	1	4
Proportion missing	0.49	1.15	0.58	0.72
Median (25th, 75th percentile)	1.00 (0.00, 10.00)	1.00 (0.00, 10.00)	1.00 (0.00, 8.00)	1.00 (0.00, 10.00)
**12 weeks**				
n	132	142	115	389
Number missing	73	32	58	163
Proportion missing	35.61	18.39	33.53	29.53

For peer review only

Median (25th, 75th percentile)	1.00 (0.00, 9.50)	2.00 (0.00, 10.00)	2.00 (0.00, 8.00)	2.00 (0.00, 10.00)
**24 weeks**				
n	123	107	89	319
Number missing	82	67	84	233
Proportion missing	40.00	38.51	48.55	42.21
Median (25th, 75th percentile)	1.00 (0.00, 9.00)	2.00 (0.00, 10.00)	2.00 (0.00, 12.00)	2.00 (0.00, 10.00)
**TOBACCO**				
**Baseline**				
Used tobacco in last 30 days	198 (97)	167 (96)	166 (96)	531 (96)
No tobacco use in last 30 days	7 (3)	7 (4)	7 (4)	21 (4)
Missing	*0*	*0*	*0*	*0*
**12 weeks**				
Used tobacco in last 30 days	129 (98)	134 (94)	107 (93)	370 (95)
No tobacco use in last 30 days	3 (2)	8 (6)	8 (7)	19 (5)
Missing	*73*	*32*	*58*	*163*
**24 weeks**				
Used tobacco in last 30 days	119 (97)	101 (93)	79 (89)	299 (93)
No tobacco use in last 30 days	4 (3)	8 (7)	10 (11)	22 (7)
Missing	*82*	*65*	*84*	*231*
**Number of days taking tobacco in last 30 days**				
**Baseline**				
n	198	171	171	540
Number missing	7	3	2	12
Proportion missing	3.41	1.72	1.16	2.17
Median (25th, 75th percentile)	30.00 (30.00, 30.00)	30.00 (30.00, 30.00)	30.00 (30.00, 30.00)	30.00 (30.00, 30.00)
**12 weeks**				
n	131	139	113	383
Number missing	74	35	60	169
Proportion missing	36.10	20.11	34.68	30.62

For peer review only

Median (25th, 75th percentile)	30.00 (30.00, 30.00)	30.00 (30.00, 30.00)	30.00 (30.00, 30.00)	30.00 (30.00, 30.00)
**24 weeks**				
n	123	109	87	319
Number missing	82	65	86	233
Proportion missing	40.00	37.36	49.71	42.21
Median (25th, 75th percentile)	30.00 (30.00, 30.00)	30.00 (30.00, 30.00)	30.00 (30.00, 30.00)	30.00 (30.00, 30.00)

Table 3 Odds ratios for Opiate Treatment Index (OPI) measures of substance use in the last 30 days assessed at 12 and 24 weeks: Use in last 30 days (binary) and N of days used.

	Treatment group difference estimate	Lower confidence limit	Upper confidence limit	p-value
**CRACK**<br>**Use in last 30 days Y/N across groups – GEE model results**				
12 weeks CM Attendance v CM Abstinence	0.83	0.37	1.85	0.654
12 weeks TAU v CM Abstinence	0.90	0.46	1.78	0.768
12 weeks TAU v CM Attendance	1.08	0.59	1.99	0.795
24 weeks CM Attendance v CM Abstinence	0.71	0.37	1.37	0.307
24 weeks TAU v CM Abstinence	0.75	0.35	1.64	0.477
24 weeks TAU v CM Attendance	1.06	0.50	2.27	0.878
**Mean differences N of days used in last 30 days across groups**				
12 weeks CM Attendance v CM Abstinence	-1.80	-3.90	0.30	0.093
12 weeks TAU v CM Abstinence	-1.67	-3.84	0.50	0.132
12 weeks TAU v CM Attendance	0.13	-2.02	2.29	0.903
24 weeks CM Attendance v CM Abstinence	0.06	-2.13	2.25	0.956
24 weeks TAU v CM Abstinence	-0.30	-2.58	1.99	0.800
24 weeks TAU v CM Attendance	-0.36	-2.67	1.96	0.762

For peer review only

**BENZODIAZEPENES**<br>**Use in last 30 days Y/N across groups – GEE model results**				
12 weeks CM Attendance v CM Abstinence	0.65	0.20	2.13	0.480
12 weeks TAU v CM Abstinence	1.31	0.51	3.41	0.573
12 weeks TAU v CM Attendance	2.01	0.57	7.09	0.276
24 weeks CM Attendance v CM Abstinence	0.46	0.15	1.39	0.167
24 weeks TAU v CM Abstinence	0.88	0.33	2.32	0.794
24 weeks TAU v CM Attendance	1.92	0.70	5.28	0.204
**Mean differences N of days used in last 30 days across groups**				
12 weeks CM Attendance v CM Abstinence	-0.16	-0.94	0.62	0.688
12 weeks TAU v CM Abstinence	0.32	-0.49	1.14	0.436
12 weeks TAU v CM Attendance	0.48	-0.32	1.29	0.239
24 weeks CM Attendance v CM Abstinence	0.23	-0.61	1.06	0.591
24 weeks TAU v CM Abstinence	0.01	-0.87	0.90	0.976
24 weeks TAU v CM Attendance	-0.22	-1.12	0.69	0.642
**ALCOHOL**<br>**Use in last 30 days Y/N across groups – GEE model results**				
12 weeks CM Attendance v CM Abstinence	1.34	0.81	2.21	0.255
12 weeks TAU v CM Abstinence	1.55	0.78	3.07	0.208
12 weeks TAU v CM Attendance	1.16	0.59	2.29	0.674
24 weeks CM Attendance v CM Abstinence	1.86	1.01	3.45	0.048
24 weeks TAU v CM Abstinence	1.08	0.66	1.77	0.762
24 weeks TAU v CM Attendance	0.58	0.29	1.17	0.126
**Mean differences N of days used in last 30 days across groups**				
12 weeks CM Attendance v CM Abstinence	1.07	-0.59	2.72	0.206
12 weeks TAU v CM Abstinence	1.00	-0.74	2.75	0.259
12 weeks TAU v CM Attendance	-0.06	-1.78	1.65	0.943
24 weeks CM Attendance v CM Abstinence	0.20	-1.56	1.96	0.825
24 weeks TAU v CM Abstinence	0.87	-0.98	2.72	0.357

For peer review only

24 weeks TAU v CM Attendance	0.67	-1.21	2.56	0.485
**TOBACCO**<br>**Mean differences N of days used in last 30 days across groups**				
12 weeks CM Attendance v CM Abstinence	-0.50	-2.10	1.09	0.536
12 weeks TAU v CM Abstinence	-0.25	-1.93	1.42	0.769
12 weeks TAU v CM Attendance	0.25	-1.40	1.90	0.765
24 weeks CM Attendance v CM Abstinence	-0.95	-2.64	0.75	0.273
24 weeks TAU v CM Abstinence	-2.07	-3.87	-0.27	0.024
24 weeks TAU v CM Attendance	-1.13	-2.96	0.71	0.228

For peer review only

Table 4. **Secondary Outcome Measures Summary Statistics: Alcohol Use Disorders (AUDIT), Social Functioning (OTI), physical and mental wellbeing (SF-36), depression and anxiety (HADS), crime (AD-SUS).**

	CM Abstinence n=205	CM Attendance n = 174	TAU n= 173	Total n = 552
**AUDIT (Alcohol Use)**				
**Baseline**				
n	205	174	173	552
Number missing	0	0	0	0
Proportion missing	0	0	0	0
Mean (SD)	9.69 (10.42)	9.37 (9.71)	9.18 (9.88)	9.43 (10.02)
**12 weeks**				
n	131	142	116	389
Number missing	74	32	57	163
Proportion missing	36.10	18.39	32.95	29.53
Mean (SD)	8.79 (9.47)	8.39 (9.72)	8.13 (8.22)	8.45 (9.19)
**24 weeks**				
n	123	108	89	320
Number missing	82	66	84	232
Proportion missing	40.00	37.93	48.55	42.03
Mean (SD)	9.67 (9.46)	7.94 (9.01)	8.67 (8.62)	8.81 (9.08)
**SOCIAL FUNCTIONING**				
**Baseline**				
n	205	174	173	552
Number missing	0	0	0	0
Proportion missing	0	0	0	0
Mean (SD)	19.59 (6.36)	17.80 (7.41)	18.29 (6.41)	18.62 (6.75)
**12 weeks**				

For peer review only

n	132	143	115	390
Number missing	73	31	58	162
Proportion missing	35.61	17.82	33.53	29.35
Mean (SD)	17.45 (7.36)	16.31 (6.25)	16.37 (6.39)	16.71 (6.69)
**24 weeks**				
n	123	109	89	321
Number missing	82	65	84	231
Proportion missing	40	37.36	48.55	41.85
Mean (SD)	16.46 (7.07)	15.63 (6.69)	15.91 (5.96)	16.02 (6.64)
**MENTAL WELLBEING (SF36)**				
**Baseline**				
n	204	173	173	550
Number missing	1	1	0	2
Proportion missing	0.49	0.57	0	0.36
Mean (SD)	30.29 (14.02)	31.88 (13.98)	30.15 (15.14)	30.75 (14.36)
**12 weeks**				
n	132	140	115	387
Number missing	73	33	58	164
Proportion missing	35.61	18.97	33.53	29.71
Mean (SD)	37.44 (14.95)	37.56 (14.02)	34.12 (15.65)	36.50 (14.88)
**24 weeks**				
n	121	109	88	318
Number missing	84	65	85	234
Proportion missing	40.98	37.36	49.13	42.39
Mean (SD)	35.97 (13.68)	36.38 (14.22)	38.43 (15.19)	36.79 (14.29)
**PHYSICAL WELLBEING (SF36)**				
**Baseline**				
n	204	173	173	550
Number missing	1	1	0	2

For peer review only

Proportion missing	0.49	0.57	0	0.36
Mean (SD)	47.06 (8.84)	46.69 (10.62)	48.02 (11.15)	47.25 (10.17)
**12 weeks**				
n	132	141	115	388
Number missing	73	33	58	164
Proportion missing	35.61	18.97	33.53	29.71
Mean (SD)	48.37 (10.02)	47.72 (11.08)	47.06 (11.14)	47.74 (10.73)
**24 weeks**				
n	121	109	88	318
Number missing	84	65	85	234
Proportion missing	40.98	37.36	49.13	42.39
Mean (SD)	49.20 (10.11)	48.09 (11.59)	48.72 (10.96)	48.69 (10.85)
**DEPRESSION (HADS)**				
**Baseline**				
n	205	173	173	551
Number missing	0	1	0	1
Proportion missing	0	0.57	0	0.18
Mean (SD)	9.51 (4.42)	9.42 (5.00)	9.46 (4.84)	9.47 (4.73)
**12 weeks**				
n	132	142	116	390
Number missing	73	32	57	162
Proportion missing	35.61	18.39	32.95	29.35
Mean (SD)	7.58 (5.07)	7.59 (4.68)	8.28 (4.75)	7.79 (4.83)
**24 weeks**				
n	123	109	89	321
Number missing	82	65	84	231
Proportion missing	40	37.36	48.55	41.85
Mean (SD)	7.72 (4.44)	7.46 (4.72)	7.02 (5.46)	7.44 (4.83)
**ANXIETY (HADS)**				

For peer review only

**Baseline**				
n	205	173	173	551
Number missing	0	1	0	1
Proportion missing	0	0.57	0	0.18
Mean (SD)	10.58 (5.12)	9.75 (5.20)	10.31 (5.44)	10.23 (5.25)
**12 weeks**				
n	132	142	116	390
Number missing	73	32	57	162
Proportion missing	35.61	18.39	32.95	29.35
Mean (SD)	8.79 (5.21)	8.77 (5.18)	9.59 (5.55)	9.02 (5.30)
**24 weeks**				
n	123	109	89	321
Number missing	82	65	84	231
Proportion missing	40	37.36	48.55	41.85
Mean (SD)	9.10 (5.13)	8.71 (5.11)	7.83 (5.78)	8.61 (5.32)

For peer review only

Table 5. **Odds ratios for Secondary Outcome Measures: Social Functioning (OPI), Alcohol Use Disorders (AUDIT) physical and mental wellbeing (SF-36), quality of life (EQ-5D) depression and anxiety (HADS).**

	Treatment group difference estimate	Lower confidence limit	Upper confidence limit	p-value
**Mean differences in AUDIT across groups**				
12 weeks CM Attendance v CM Abstinence	0.42	-1.17	2.02	0.605
12 weeks TAU v CM Abstinence	0.45	-1.22	2.11	0.600
12 weeks TAU v CM Attendance	0.02	-1.62	1.67	0.977
24 weeks CM Attendance v CM Abstinence	-0.99	-2.68	0.69	0.247
24 weeks TAU v CM Abstinence	-0.17	-1.93	1.60	0.854
24 weeks TAU v CM Attendance	0.83	-0.97	2.63	0.366
**Mean differences in OPIATE TREATMENT INDEX Social Functioning across groups**				
12 weeks CM Attendance v CM Abstinence	0.24	-1.06	1.54	0.715
12 weeks TAU v CM Abstinence	-0.08	-1.44	1.28	0.912
12 weeks TAU v CM Attendance	-0.32	-1.65	1.02	0.640
24 weeks CM Attendance v CM Abstinence	0.32	-1.07	1.70	0.655
24 weeks TAU v CM Abstinence	0.10	-1.36	1.56	0.889
24 weeks TAU v CM Attendance	-0.21	-1.70	1.28	0.781
**Mean differences in SF36 MCS across groups**				
12 weeks CM Attendance v CM Abstinence	-1.08	-4.45	2.29	0.529
12 weeks TAU v CM Abstinence	-2.49	-5.98	1.00	0.162
12 weeks TAU v CM Attendance	-1.41	-4.89	2.07	0.427
24 weeks CM Attendance v CM Abstinence	-1.76	-5.29	1.77	0.330
24 weeks TAU v CM Abstinence	1.19	-2.49	4.87	0.526
24 weeks TAU v CM Attendance	2.95	-0.80	6.69	0.123
**Mean differences in SF36 PCS across groups**				
12 weeks CM Attendance v CM Abstinence	-0.35	-2.33	1.62	0.727

For peer review only

12 weeks TAU v CM Abstinence	-2.06	-4.13	0.02	0.052
12 weeks TAU v CM Attendance	-1.70	-3.75	0.35	0.103
24 weeks CM Attendance v CM Abstinence	-0.90	-3.01	1.21	0.402
24 weeks TAU v CM Abstinence	-0.75	-2.98	1.48	0.511
24 weeks TAU v CM Attendance	0.15	-2.11	2.42	0.895
**Mean differences in HADS depression across groups**				
12 weeks CM Attendance v CM Abstinence	0.08	-1.05	1.22	0.885
12 weeks TAU v CM Abstinence	0.69	-0.48	1.87	0.249
12 weeks TAU v CM Attendance	0.61	-0.56	1.78	0.309
24 weeks CM Attendance v CM Abstinence	-0.17	-1.36	1.03	0.784
24 weeks TAU v CM Abstinence	-0.62	-1.86	0.63	0.331
24 weeks TAU v CM Attendance	-0.45	-1.72	0.82	0.487
**Mean differences in HADS anxiety across groups**				
12 weeks CM Attendance v CM Abstinence	0.46	-0.84	1.76	0.485
12 weeks TAU v CM Abstinence	0.33	-1.00	1.67	0.624
12 weeks TAU v CM Attendance	-0.13	-1.46	1.21	0.851
24 weeks CM Attendance v CM Abstinence	0.48	-0.87	1.83	0.482
24 weeks TAU v CM Abstinence	-0.81	-2.21	0.58	0.252
24 weeks TAU v CM Attendance	-1.30	-2.72	0.12	0.073

For peer review only

Table 6. ARM-5 – Therapeutic Alliance

	CM Abstinence n=205	CM Attendance n = 174	TAU n= 173	Total n = 552
**ARM-5 mean score**				
**Week 4**				
n	91	84	36	211
Number missing	114	90	137	341
Proportion missing	55.61	51.72	79.19	61.78
Mean (SD)	6.54 (0.73)	6.71 (0.63)	6.26 (1.09)	6.56 (0.78)
**Week 8**				
n	63	69	28	160
Number missing	142	105	145	392
Proportion missing	69.27	60.34	83.82	71.01
Mean (SD)	6.60 (0.78)	6.59 (0.69)	6.16 (1.01)	6.52 (0.80)
**Week 12**				
n	123	126	91	340
Number missing	82	48	82	212
Proportion missing	40.00	27.59	47.40	38.41
Mean (SD)	6.43 (0.96)	6.27 (1.27)	5.88 (1.30)	6.23 (1.19)

For peer review only

Table 1: CONSORT 2010 checklist of information to include when reporting a cluster randomised trial

Section/Topic	Item No	Standard Checklist item	Extension for cluster designs	Page No *
**Title and abstract**				
	1a	Identification as a randomised trial in the title	Identification as a cluster randomised trial in the title	1
	1b	Structured summary of trial design, methods, results, and conclusions (for specific guidance see CONSORT for abstracts)[1,2]	See table 2	2
**Introduction**				
**Background and objectives**	2a	Scientific background and explanation of rationale	Rationale for using a cluster design	4
	2b	Specific objectives or hypotheses	Whether objectives pertain to the the cluster level, the individual participant level or both	4
**Methods**				
**Trial design**	3a	Description of trial design (such as parallel, factorial) including allocation ratio	Definition of cluster and description of how the design features apply to the clusters	3/4/5
	3b	Important changes to methods after trial commencement (such as eligibility criteria), with reasons		**7**
**Participants**	4a	Eligibility criteria for participants	Eligibility criteria for clusters	4
	4b	Settings and locations where the data were collected		**3**
**Interventions**	5	The interventions for each group with sufficient details to allow replication, including how and when they were actually administered	Whether interventions pertain to the cluster level, the individual participant level or both	5

**Outcomes**	6a	Completely defined pre-specified primary and secondary outcome measures, including how and when they were assessed	Whether outcome measures pertain to the cluster level, the individual participant level or both	6
	6b	Any changes to trial outcomes after the trial commenced, with reasons		**N/A**
**Sample size**	7a	How sample size was determined	Method of calculation, number of clusters(s) (and whether equal or unequal cluster sizes are assumed), cluster size, a coefficient of intracluster correlation (ICC or *k*), and an indication of its uncertainty	6/7
	7b	When applicable, explanation of any interim analyses and stopping guidelines		
**Randomisation:**				
**Sequence generation**	8a	Method used to generate the random allocation sequence		**4/5**
	8b	Type of randomisation; details of any restriction (such as blocking and block size)	Details of stratification or matching if used	4/5
**Allocation concealment mechanism**	9	Mechanism used to implement the random allocation sequence (such as sequentially numbered containers), describing any steps taken to conceal the sequence until interventions were assigned	Specification that allocation was based on clusters rather than individuals and whether allocation concealment (if any) was at the cluster level, the individual participant level or both	5
**Implementation**	10	Who generated the random allocation sequence, who enrolled participants, and who assigned participants to interventions	Replace by 10a, 10b and 10c	4/5
	10a		Who generated the random allocation sequence, who enrolled clusters, and who	4/5

			assigned clusters to interventions	
	10b		Mechanism by which individual participants were included in clusters for the purposes of the trial (such as complete enumeration, random sampling)	4/5
	10c		From whom consent was sought (representatives of the cluster, or individual cluster members, or both), and whether consent was sought before or after randomisation	6
**Blinding**	11a	If done, who was blinded after assignment to interventions (for example, participants, care providers, those assessing outcomes) and how		**5**
	11b	If relevant, description of the similarity of interventions		**5**
**Statistical methods**	12a	Statistical methods used to compare groups for primary and secondary outcomes	How clustering was taken into account	7
	12b	Methods for additional analyses, such as subgroup analyses and adjusted analyses		**7**
**Results**				
**Participant flow (a diagram is strongly recommended)**	13a	For each group, the numbers of participants who were randomly assigned, received intended treatment, and were analysed for the primary outcome	For each group, the numbers of clusters that were randomly assigned, received intended treatment, and were analysed for the primary outcome	Figure 1 (page 8)

	13b	For each group, losses and exclusions after randomisation, together with reasons	For each group, losses and exclusions for both clusters and individual cluster members	Figure 1 and supplementary materials table 1
**Recruitment**	14a	Dates defining the periods of recruitment and follow-up		**Page 8**
	14b	Why the trial ended or was stopped		**N/A**
**Baseline data**	15	A table showing baseline demographic and clinical characteristics for each group	Baseline characteristics for the individual and cluster levels as applicable for each group	Table 1, 4 & 5 and supplementary materials Tables 2-4
**Numbers analysed**	16	For each group, number of participants (denominator) included in each analysis and whether the analysis was by original assigned groups	For each group, number of clusters included in each analysis	Figure 1, Table 2,4,5.
**Outcomes and estimation**	17a	For each primary and secondary outcome, results for each group, and the estimated effect size and its precision (such as 95% confidence interval)	Results at the individual or cluster level as applicable and a coefficient of intracluster correlation (ICC or k) for each primary outcome	Pages 10/11 . tables 3.Supplementary Table 3 & 5
	17b	For binary outcomes, presentation of both absolute and relative effect sizes is recommended		**Pages 10/11**
**Ancillary analyses**	18	Results of any other analyses performed, including subgroup analyses and adjusted analyses, distinguishing pre-specified from exploratory		**Pages 10/11**
**Harms**	19	All important harms or unintended effects in each group (for specific guidance see CONSORT for harms[3])		**Page 11**
**Discussion**				
**Limitations**	20	Trial limitations, addressing sources of potential bias,		**Page 12 & 13**

		imprecision, and, if relevant, multiplicity of analyses		
**Generalisability**	21	Generalisability (external validity, applicability) of the trial findings	Generalisability to clusters and/or individual participants (as relevant)	Page 13
**Interpretation**	22	Interpretation consistent with results, balancing benefits and harms, and considering other relevant evidence		**Page 12 & 13**
**Other information**				
**Registration**	23	Registration number and name of trial registry		**Page 16**
**Protocol**	24	Where the full trial protocol can be accessed, if available		**Attached document. Published Protocol.**
**Funding**	25	Sources of funding and other support (such as supply of drugs), role of funders		**Page 16**

** Note: page numbers optional depending on journal requirements*

For peer review only

Table 2: Extension of CONSORT for abstracts[1,2] to reports of cluster randomised trials

Item	Standard Checklist item	Extension for cluster trials
**Title**	Identification of study as randomised	**Identification of study as cluster randomised**
**Trial design**	Description of the trial design (e.g. parallel, cluster, non-inferiority)	
**Methods**		
**Participants**	Eligibility criteria for participants and the settings where the data were collected	**Eligibility criteria for clusters**
**Interventions**	Interventions intended for each group	
**Objective**	Specific objective or hypothesis	**Whether objective or hypothesis pertains to the cluster level, the individual participant level or both**
**Outcome**	Clearly defined primary outcome for this report	**Whether the primary outcome pertains to the cluster level, the individual participant level or both**
**Randomization**	How participants were allocated to interventions	**How clusters were allocated to interventions**
**Blinding (masking)**	Whether or not participants, care givers, and those assessing the outcomes were blinded to group assignment	
**Results**		
**Numbers randomized**	Number of participants randomized to each group	**Number of clusters randomized to each group**
**Recruitment**	Trial status[1]	
**Numbers analysed**	Number of participants analysed in each group	**Number of clusters analysed in each group**
**Outcome**	For the primary outcome, a result for each group and the estimated effect size and its precision	**Results at the cluster or individual participant level as applicable for each primary outcome**
**Harms**	Important adverse events or side effects	
**Conclusions**	General interpretation of the results	
**Trial registration**	Registration number and name of trial register	
**Funding**	Source of funding	

[1] Relevant to Conference Abstracts

REFERENCES

[1] Hopewell S, Clarke M, Moher D, Wager E, Middleton P, Altman DG, et al. CONSORT for reporting randomised trials in journal and conference abstracts. *Lancet* 2008, 371:281-283

[2] Hopewell S, Clarke M, Moher D, Wager E, Middleton P, Altman DG at al (2008) CONSORT for reporting randomized controlled trials in journal and conference abstracts: explanation and elaboration. *PLoS Med* 5(1): e20

[3] Ioannidis JP, Evans SJ, Gotzsche PC, O'Neill RT, Altman DG, Schulz K, Moher D. Better reporting of harms in randomized trials: an extension of the CONSORT statement. *Ann Intern Med* 2004; 141(10):781-788.

For peer review only
